# Genome-wide assessment of population structure and association mapping for agronomic and grain nutritional traits in proso millet (*Panicum miliaceum* L.)

**DOI:** 10.1038/s41598-024-72319-w

**Published:** 2024-09-19

**Authors:** Mani Vetriventhan, Hari D. Upadhyaya, Santosh Deshpande, Matthew S. Johnson, Jason G. Wallace, Allan Victor, D. Naresh, Laavanya Rayaprolu, Kuldeep Singh, Sean Mayes

**Affiliations:** 1 International Crops Research Institute for the Semi-Arid Tropics (ICRISAT), Patancheru, Hyderabad, Telangana 502324 India; 2 Institute of Plant Breeding, Genetics, and Genomics, University of Georgia, Athens, GA USA; 3 Department of Crop and Soil Science, University of Georgia, Athens, GA USA; 4 Tamil Nadu Agricultural University, Coimbatore, Tamil Nadu India; 5 Wageningen University and Research, Wageningen, The Netherlands; 6 Present Address: University of Georgia, Athens, GA 30605 USA; 7 Present Address: Hytech Seed India Private Limited, Hyderabad, India

**Keywords:** Proso millet, Genome-wide association mapping, Agronomic traits, Grain nutrients, Marker-traits associations, Diversity, Population structure, Genetics, Agricultural genetics

## Abstract

Proso millet is an important but under-researched and underutilized crop with the potential to become a future smart crop because of its climate-resilient features and high nutrient content. Assessing diversity and marker-trait associations are essential to support the genomics-assisted improvement of proso millet. This study aimed to assess the population structure and diversity of a proso millet diversity panel and identify marker-trait associations for agronomic and grain nutrient traits. In this study, genome-wide single nucleotide polymorphisms (SNPs) were identified by mapping raw genotyping-by-sequencing (GBS) data onto the proso millet genome, resulting in 5621 quality-filtered SNPs in 160 diverse accessions. The modified Roger's Distance assessment indicated an average distance of 0.268 among accessions, with the race *miliaceum* exhibiting the highest diversity and *ovatum* the lowest. Proso millet germplasm diversity was structured according to geographic centers of origin and domestication. Genome-wide association mapping identified 40 marker-trait associations (MTAs), including 34 MTAs for agronomic traits and 6 for grain nutrients; 20 of these MTAs were located within genes. Favourable alleles and phenotypic values were estimated for all MTAs. This study provides valuable insights into the population structure and diversity of proso millet, identified marker-trait associations, and reported favourable alleles and their phenotypic values for supporting genomics-assisted improvement efforts in proso millet.

## Introduction

Traditionally important, climate-resilient and nutrient-rich crops have a significant role to play in the near future to achieve food security and nutrition despite global climate change. Proso millet (*Panicum miliaceum* L.) is one of the oldest domesticated cereal crops in the world. The earliest records of proso millet occurrence were from China between 10,300 and 8700 cal BP^1^ and Eastern Europe at 7000 cal BP^2^. This pattern suggests independent domestication in Central Asia and Eastern Europe, or that they may have originated from domestication within China and then spread westward across the Eurasian Steppe^3^. Proso millet belongs to a group of small-seeded cereal crops known as small millets. It is also popularly known as broomcorn millet, common millet, panic millet, and hog millet in different parts of the world. Proso millet is grown in Asia, Australia, North America, Europe, and Africa^4^; however, it is a minor crop globally in terms of its contribution to global production^5^. Proso millet remains a locally important staple source of food security in semi-arid regions, where other cereals fail, whereas in developed countries it is used for feeding birds and livestock. County-wise, proso millet is cultivated on about 0.82 m ha in Russia, 0.32 m ha in China^6^, 0.20 m ha in the USA^7^, 0.03 m ha in India^8^ and 0.002 m ha in Korea^9^. The USA is one of the top producers of proso millet and exports 15–20% of its annual production to over 70 countries^7^.

Proso millet is a C_4_ allotetraploid crop. Its important characteristic features include short duration (matures in 40–80 days), low water requirements, high drought tolerance, and good adaptability to different environmental conditions. Its grains are highly nutritious and gluten-free, and they contain higher contents of protein, dietary fiber, several minerals, vitamins, and antioxidants than most other cereals^10,11^. The protein content of proso millet (12.5%) is higher than that of rice (7.9%), maize (9.2%), wheat (11.6%), and other millets, and it is also significantly richer in essential amino acids (leucine, isoleucine, and methionine) than wheat^10,12^. These climate-resilient and nutrient-rich features of proso millet (and other minor but regionally important crops) have the potential to ensure food security and nutrition, and crop diversification.

Proso millet is an under-researched and underutilized crop compared with other major cereals. Globally, approximately 29,000 germplasm accessions have been conserved in genebanks, and high variability exists^13^. Based on panicle morphology and shape, the cultivated germplasm of proso millet can be grouped into five races: *miliaceum*, *patentissimum*, *contractum*, *compactum* and *ovatum*^14^. Germplasm diversity could potentially contribute to proso millet improvement, provided that these are subject to systematic evaluation, identification of trait-specific sources, and genomic investigation. Evaluation of germplasm for important traits such as productivity, biotic and abiotic stress tolerance, and grain nutrient traits resulted in the identification of promising accessions for crop improvement^15^. Genomics-assisted improvement in proso millet is very limited. However, the availability of draft genome sequences of proso millet^16,17^ provides an opportunity to investigate the diversity, structure, and identification of QTLs using next-generation sequencing approaches. Genome-wide association studies (GWAS) are an important approach for the genetic dissection of complex traits and for identifying marker-trait associations and have been applied in several cereal crops, including rice, wheat, sorghum, and foxtail millet, for many traits, including agronomic, quality, and adaptation traits^18–23^. In proso millet, only two GWAS reports are available for agronomic and seed traits^24,25^. The present study aimed to (1) assess the diversity and population structure of the global proso millet germplasm collection conserved at the International Crops Research Institute for the Semi-Arid Tropics (ICRISAT) genebank and (2) identify genomic regions associated with productivity and grain nutrients.

## Results

### Phenotypic variation

**Fig. 1:**
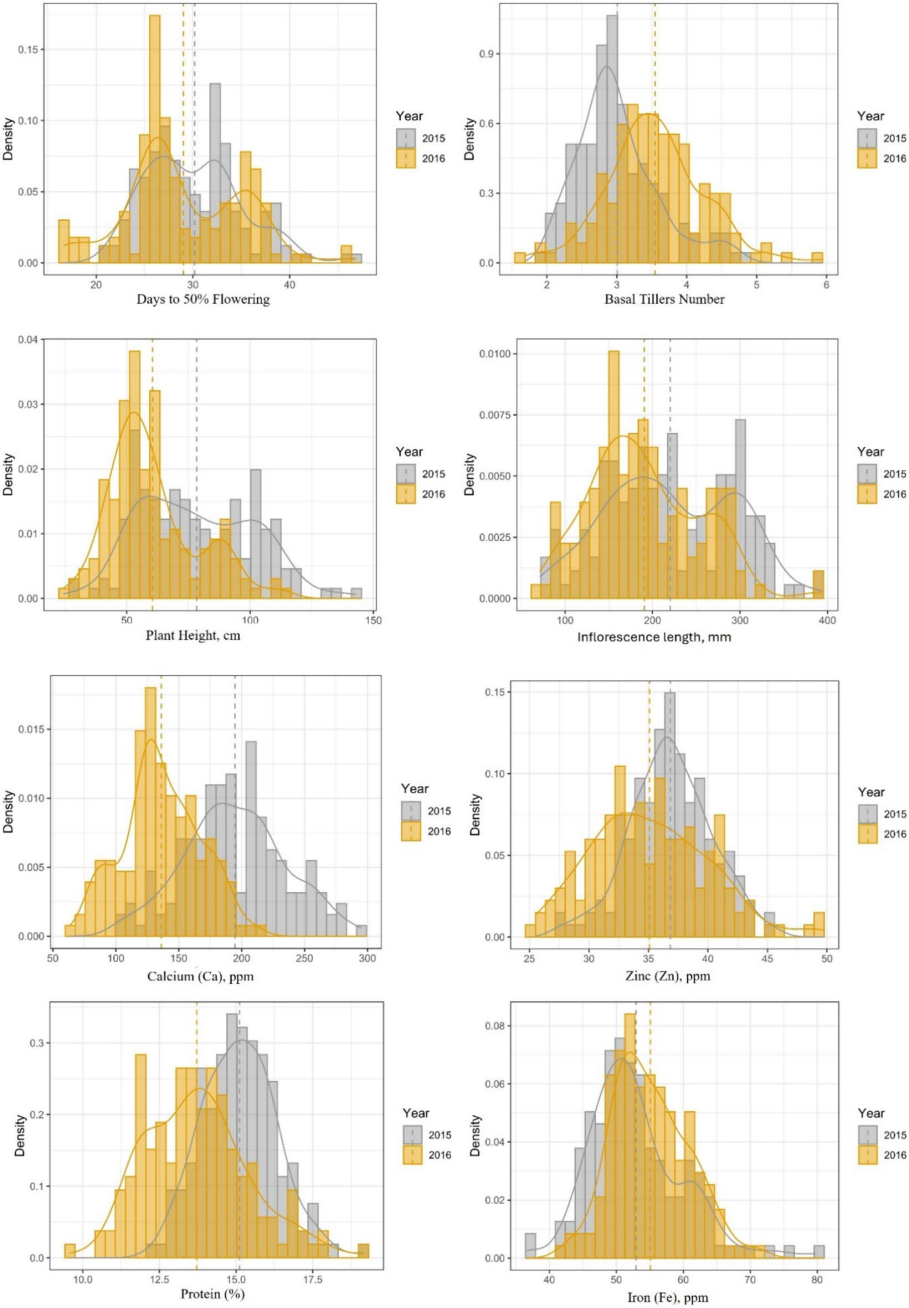
Frequency distribution of key agronomic and grain nutrient traits of proso millet evaluated in 2015 and 2016 at ICRISAT Patancheru, India.

A proso millet diversity panel, consisting of 200 lines, was used in this study. These lines originated in 30 countries and represent all five races of proso millet (60.5% *miliaceum*, 12.5% *compactum*, 12.0% *contractum*, 8.5% *patentissimum*, and 6.5% *ovatum*). The phenotypic variability of this diversity panel (200 accessions) for agronomic and grain nutrient traits has been described in detail in our previous study^26^. In brief, the phenotypic evaluation indicated a significant genotypic variance and genotype × year variance for all traits except for basal tiller number, indicating the significant influence of genotype and environment and their interaction on the expression of traits. All but two traits showed high broad-sense heritability (> 0.60) in both years and when combined across years. The exceptions were basal tiller number and grain Fe content, which showed moderate heritability of 0.30–0.60^26^. In this study, 160 out of 200 accessions were included after filtering for high-quality SNPs. The frequency distribution of key agronomic and grain nutrient traits is presented in Fig. 1, and a complete list of traits investigated is presented in Supplementary Fig. 1.

### Genome-wide SNP variation

#### SNP diversity and population structure

**Fig. 2:**
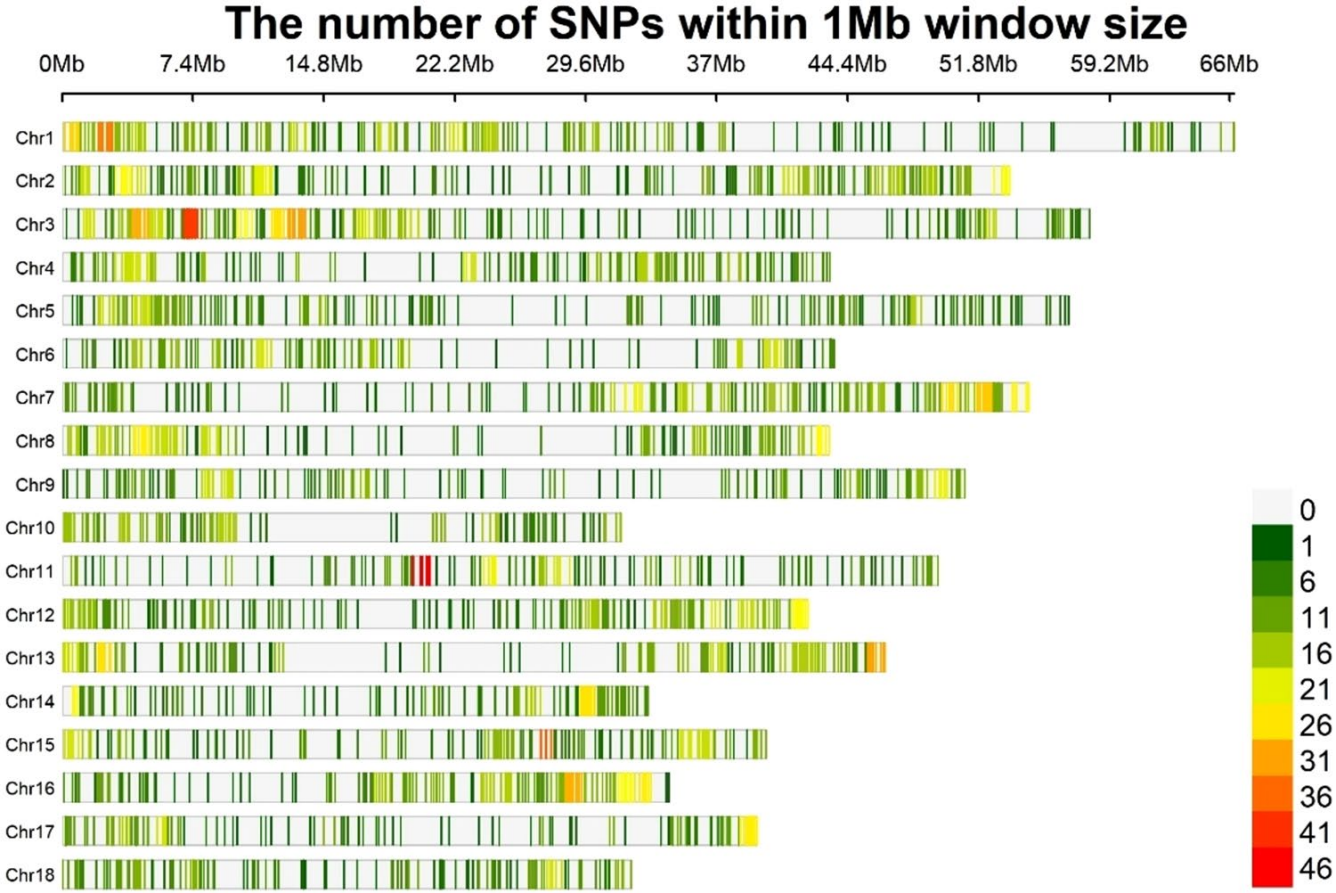
Chromosome-wise SNPs distribution, based on proso millet germplasm characterized using genotyping-by-sequencing (GBS) approach.

For the final dataset, after filtering, we retained 160 accessions with 5621 SNPs. The SNP counts varied from 204 on chromosome 10 to 476 on chromosome 3 (Table 1). The SNP distribution across the 18 chromosomes is shown in Fig. 2. Analysis of the position and distribution of each SNP locus at the whole-genome level showed that 68% of the SNPs were within 100 kbp of adjacent SNPs. Further, using SnpEff^27^, each SNP locus was annotated based on its genomic location to predict coding effects. It was found that 4.5% of the SNP loci were in the exon regions and 15.6% in intergenic regions, while 43.7% and 36.18% of SNPs were in the downstream and upstream regions of the gene, respectively.

**Table 1:** Chromosome-wise single nucleotide polymorphisms (SNPs) count of proso millet germplasm characterized using genotyping-by-sequencing (GBS) approach.

Chromosome	Start SNP position	End SNP position	SNP count
1	1,49,644	6,62,37,261	464
2	1,69,688	5,35,48,972	404
3	2,54,522	5,80,57,832	476
4	5,27,761	4,34,12,958	332
5	5,79,531	5,68,93,650	293
6	2,37,200	4,36,35,417	271
7	2,04,937	5,46,31,338	421
8	3,54,997	4,33,51,971	291
9	77,639	5,10,19,631	275
10	1,14,798	3,15,98,280	204
11	5,35,585	4,94,93,772	296
12	1,29,082	4,21,47,397	319
13	76,684	4,64,93,488	327
14	6,17,426	3,30,97,616	212
15	1,57,109	3,97,83,505	301
16	1,08,570	3,42,95,688	294
17	1,84,648	3,92,95,615	227
18	39,397	3,21,51,038	214
Total SNPs			5621

### AMOVA (analysis of molecular variance)

The AMOVA indicated the highest contribution of variation within a race (71%) and within the region (66.9%). However, a low but significant contribution was observed between races and regions (4% and 8.4%, respectively) (Table 2). This implies that traditional race classifications based on morphology are weakly correlated with the underlying population genetics.

**Table 2:** Analysis of molecular variance (AMOVA) and Monte Carlo significance tests for 160 accessions.

	Df	Sum Sq	Mean Sq	Variance %	Sigma	Phi	P-value
Variation between and within race							
Between race	4	8788.5	2197.1	4.0	24.2	0.75	0.01
Between samples within race	155	155,159.3	1001.0	71.0	425.7	0.73	0.01
Within samples (= residual error)	160	23,943.9	149.7	25.0	149.7	0.04	0.01
Total	319	187,891.8	589.0	100	599.6		
Variation between and within regions							
Between region	5	15,900.0	3180.1	8.4	51.0	0.75	0.01
Between samples within region	154	148,047.7	961.3	66.9	405.8	0.73	0.01
Within samples (= residual error)	160	23,944.00	149.7	24.7	149.7	0.08	0.01
Total	319	187,891.8	589.0	100	606.6		

### Genetic distance

**Fig. 3:**
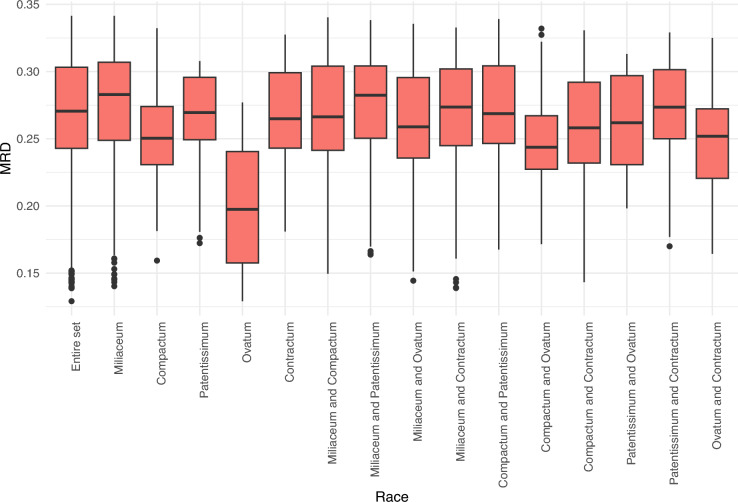
Modified Roger's distance of accessions within and among races of proso millet based on GBS-based SNP characterization of proso millet germplasm.

The average Modified Roger’s Distance (MRD) of the entire set was 0.268 and ranged from 0.126 to 0.341. Among the races, accessions belonging to the *miliaceum* race had the highest average distance (0.274), whereas the lowest distance was observed among accessions within the *ovatum* race (0.201) (Supplementary Table 1). Figure 3 shows the minimum, maximum and median MRD of the entire set within and between the races. Among races, the lowest distance was observed between *ovatum* and *contractum* (0.248), followed by *ovatum* and *compactum* (0.250), whereas race *patentissimum* showed a higher distance from other races (0.260–0.272) (Supplementary Table 1).

### Population structure

**Fig. 4:**
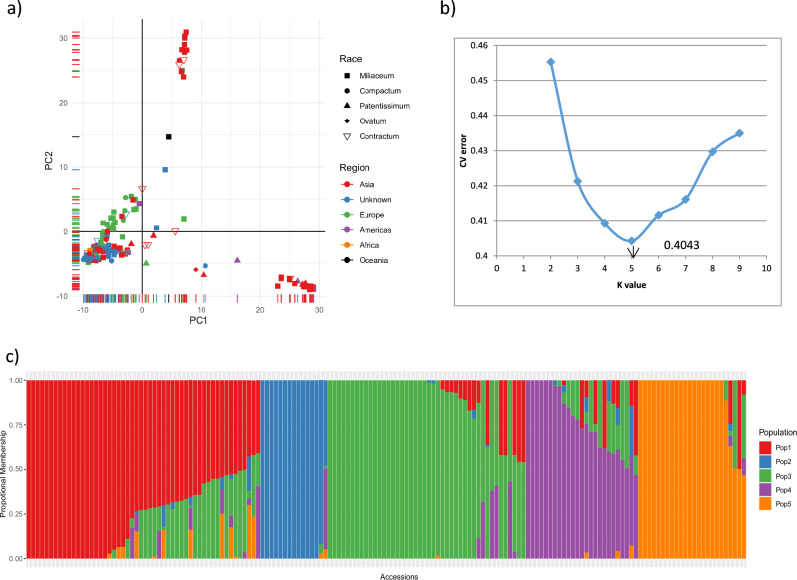
Population structure assessment of proso millet collection: (**a**) PCA biplot of 160 accessions based on SNPs from GBS, (**b**) rate of change in cross-validation (CV) error between successive k-values (k values ranging from 1 to 10), and (**c**) model-based population structure in proso millet collection based on ADMIXTURE with K = 5 populations for the 160 accessions.

Principal component analysis and ADMIXTURE were used to infer the population structure of the collection, and clear subpopulation structures were observed (Fig. 4a). The first two principal components explained 28% of the total genetic variation and aided in visually differentiating the substructures within Asian accessions. PCA also showed the presence of a substructure within the collection, based on the regions and countries within the regions. A grouping of a small clump of 14 Asian accessions in the top center of the PCA biplot showed that these accessions were diverse from the other Asian accessions; on further observation, 12 of these 14 accessions were of Korean origin, while the remaining two accessions were from China and Germany. In addition, from the biplot, it can be seen that a diverse set of 20 Asian accessions clustered at the bottom-right. Further observation of the countries of sample collection found that most of the accessions were of Indian origin (14 accessions from India) and one accession from Sri Lanka, while other accessions were from Mexico, Syria, and other countries.

The hierarchical population structure, using the model-based ADMIXTURE program, was run assuming K = 1 to 10 populations without providing any prior information on population structure. The obtained CV values and the corresponding ΔCV, combined with a line graph using CV errors for each K, showed that the CV error decreased steadily up to K = 5 and increased afterwards. This suggested the presence of five natural subpopulations (K = 5) (Fig. 4b) within our proso millet collection, and a K value of 5 was considered an appropriate population structure. The five populations were named POP1–POP5 (Fig. 4c). The accessions that had population membership of < 0.6 in all five populations were considered admixtures (accessions with genomes of two or more populations).

We assigned individuals to any of the five subpopulations considering the maximum proportion of membership. Accordingly, POP1, POP2, POP3, and POP5 represent most accessions from Asia, whereas POP4 represents most accessions from Europe (18 out of 25 accessions, including two unknown origins, one from America, and four from Asia). Accessions from Korea were grouped as POP2. Accessions of the race *miliaceum* dominated in all the populations, while the majority of accessions belonging to *compactum* were in the POP1, *ovatum* in the POP3, *patentissimum* in the POP 5, *contractum* in the POP 1 and POP 3. These distributions show that the proso millet accessions were not structured as per racial groups, while they were structured according to regions and countries within regions (for example, Korea in POP 2, Russia in POP 4), as observed in PCA. Overall, 93 of the 160 accessions had a population membership of > 0.90, while 31 accessions had a population membership of < 0.60. Approximately 17% of accessions (12 accessions) from Asia were admixture (< 0.60), while 37% of accessions from Europe had admixtures with different populations.

**Fig. 5:**
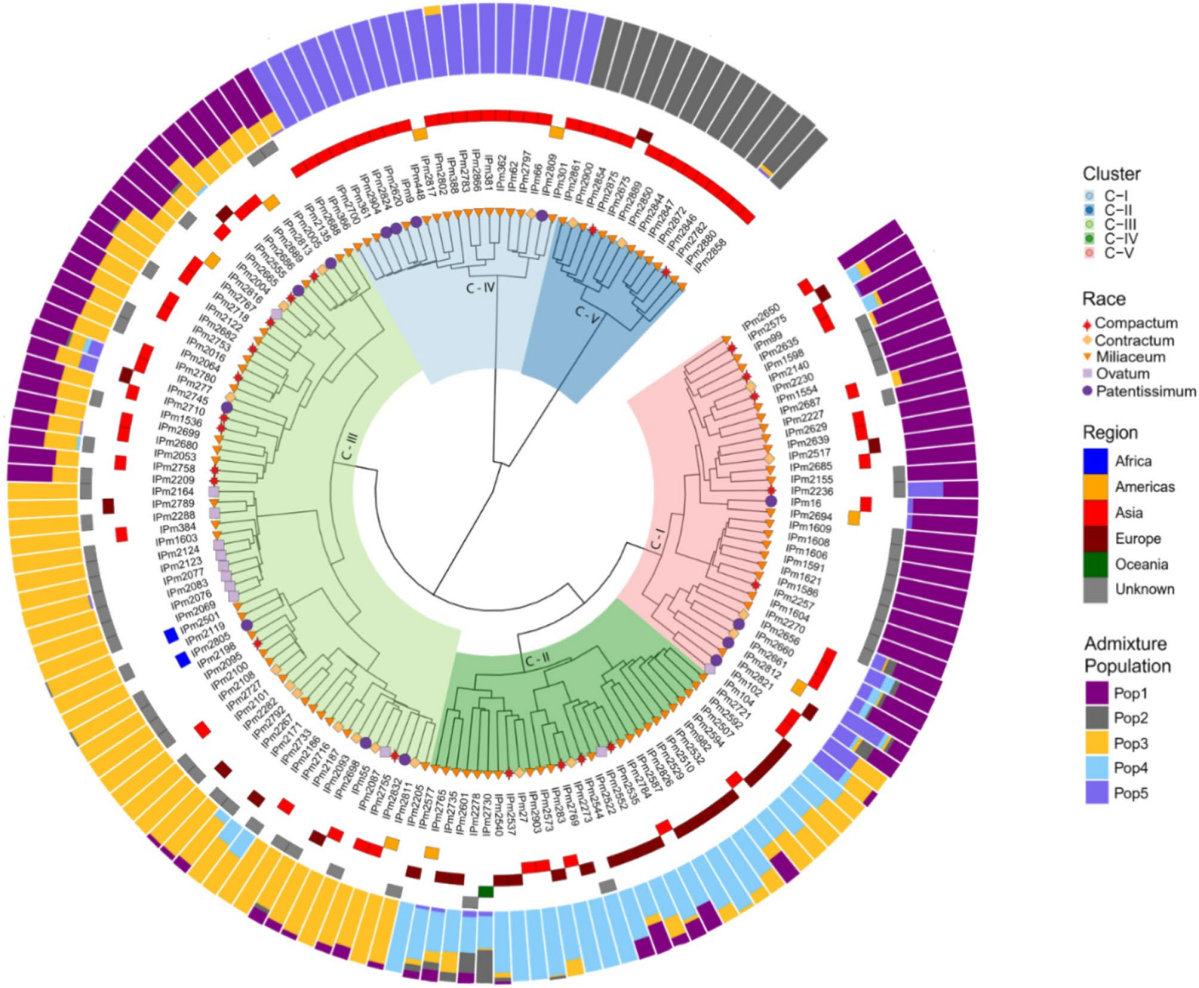
Cluster dendrogram of GBS-based SNPs, based on Modified Roger's Distances (innermost colors on the dendrogram represent clusters, shapes at the nodes of the dendrogram represent races, tiles surrounding the dendrogram represent the region, and colored outermost bars represent the ADMIXTURE proportions-based population structure.

Hierarchical clustering based on the calculated MRD showed the presence of five major clusters (Fig. 5). The cluster dendrogram results also agreed with the admixture-based population structure in terms of both the number of populations and the presence of population structure based on regions. Cluster-5 was dominated by Korean accessions and cluster-4 was dominated by Indian accessions. Combining the cluster dendrogram and ADMIXTURE-based population membership, individuals belonging to clusters 1, 4, and 5 had well-defined allelic or membership proportions with fewer admixtures.

### Linkage disequlibrium (LD) decay

**Fig. 6:**
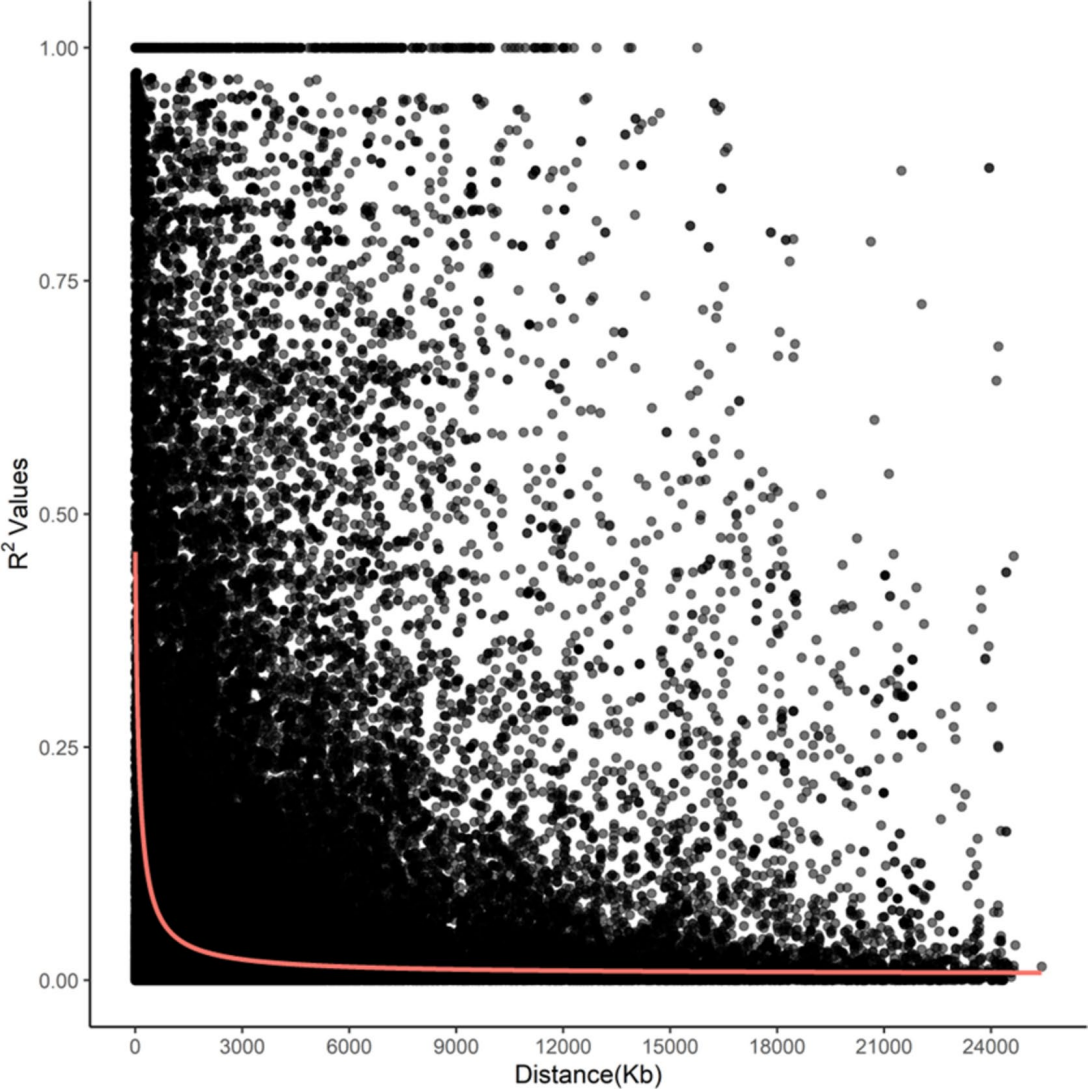
Genome-wide linkage disequilibrium (LD) decay in proso millet using GBS-based SNPs. Black dots represent individual SNP pairs on the same chromosome, while the red line shows the mean value.

The whole-genome average maximum r^2^ value was 0.52 at 5 kpb, which dropped to half that between 50 kbp (0.37) and 75 kbp (0.18), and plateaued after ~ 200 kbp (0.10 at 225 kbp to 0.05 at 30 Mbp) (Fig. 6).

### Genome-wide association study (GWAS) on agronomic and nutrient traits

**Fig. 7:**
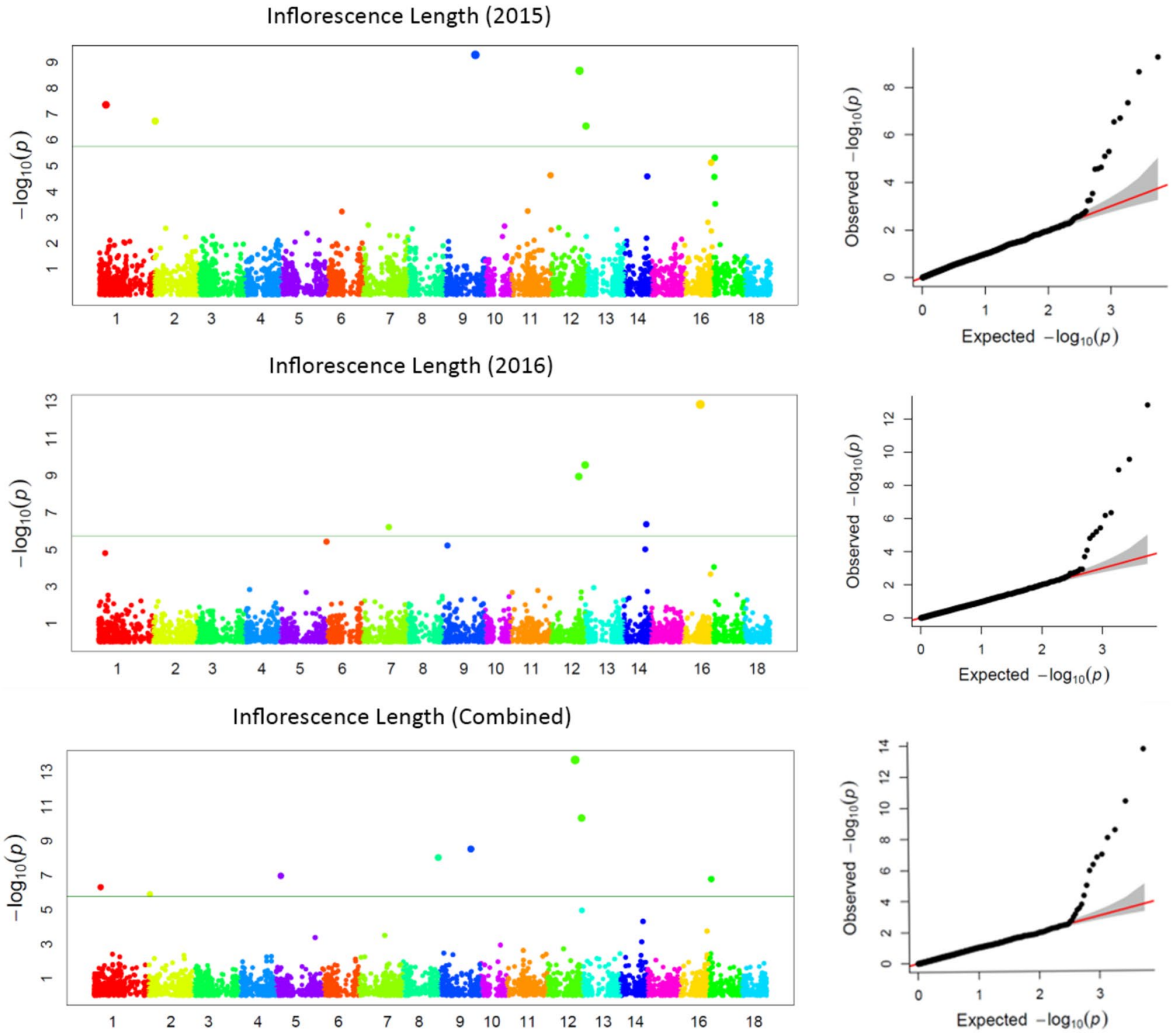
Manhattan plots and quantile–quantile (QQ) plots for inflorescence length (INFL) of proso millet evaluated in 2015 and combined for both years. The chromosomes are marked with different colors along the horizontal axis.

GWAS for agronomic traits identified 121, 95, and 95 marker-trait associations (MTAs) for 2015, 2016, and combined datasets, respectively, using FarmCPU with a p-value cutoff of ≤ 0.0001. Furthermore, when we looked for common MTAs across the three datasets (2015, 2016, and combined), 34 SNPs were found to be significantly associated with agronomic traits in at least two of them. Among these 34 MTAs, four SNPs for inflorescence length (Proso.1_8346815, Proso.14_27820106, Proso.12_34047515, and Proso.12_41890075) (Fig. 7) and one for plant height (Proso.7_1535098) were detected significant across all three datasets. Eighteen of the 34 MTAs were located in genes (Table 3). GWAS on grain nutrient traits identified 24, 37, and 26 MTAs for the 2015, 2016 and combined datasets, but only six were found in at least two of the datasets. Of these, two SNPs (Proso.17_30948407 and Proso.17_5885921, associated with Zn and Fe, respectively) on chromosome 17 were located in genes *PM17G09880* and *TE311547*.

**Table 3:** Significant marker-trait associations identified at least in two of three data set in the proso millet for agronomic and grain nutrients traits.

Trait^#^	SNP	Chromosome	Position	2015		2016		Combined		Gene annotation	Gene location
				effect	p-value	effect	p-value	effect	p-value		
BT	Proso.2_14901071	2	14,901,071	0.29	3.10E-06			0.22	4.10E-05	*PM02G15460*	PmChr02 14,899,246–14,907,188 (1)
BT	Proso.12_28525796	12	28,525,796	0.26	6.80E-06			0.21	4.90E-05	*PM12G15280*	PmChr12 28,524,818–28,527,467 (1)
DF	Proso.1_11746355	1	11,746,355			-4.27	1.70E-08	-4.3	2.10E-10	not on gene	
DF	Proso.14_29825523	14	29,825,523			1.45	1.00E-07	1.14	9.50E-06	not on gene	
PLHT	Proso.7_1535098	7	1,535,098	-17.02	3.60E-11	-18.05	9.00E-17	-14.79	5.50E-12	*TE347748*	PmChr07 1,535,090–1,535,203 (-1)
PLHT	Proso.9_39130372	9	39,130,372			-6.63	5.20E-07	-12	6.20E-13	not on gene	
FLBL	Proso.17_2816771	17	2,816,771	16.48	8.80E-05			18.74	5.50E-06	not on gene	
FLBL	Proso.18_4062363	18	4,062,363	-12.39	7.10E-05	-14.69	6.50E-06			not on gene	
FLBL	Proso.8_9836654	8	9,836,654	25.35	1.70E-08	20.62	2.50E-06			*PM08G11490*	PmChr08 9,836,493–9,842,614 (-1)
FLBL	Proso.10_24191113	10	24,191,113	14.03	3.60E-05			13.93	5.10E-05	*Denovo_TE096034*	PmChr10 24,179,287–24,191,529 (1)
FLBL	Proso.11_27996505	11	27,996,505	-14.74	2.50E-06			-21.27	4.80E-08	*TE013419*	PmChr11 27,996,274–27,996,617 (1)
FLBL	Proso.12_41890075	12	41,890,075	35.9	1.20E-08			37.77	1.90E-09	not on gene	
FLSL	Proso.7_1535098	7	1,535,098	-9.06	1.10E-09			-12.05	1.50E-16	*TE347748*	PmChr07 1,535,090–1,535,203 (-1)
FLSL	Proso.12_34047515	12	34,047,515			-2.28	2.90E-05	-5.45	2.70E-13	*PM12G21200*	PmChr12 34,040,158–34,055,027 (-1)
Nnode	Proso.16_108570	16	108,570			-0.39	2.10E-07	-0.38	1.20E-07	*Denovo_TE035623*	PmChr16 106,056–109,244 (-1)
Nnode	Proso.17_2540828	17	2,540,828	-0.33	2.10E-08			-0.35	3.70E-13	not on gene	
Nnode	Proso.5_6473183	5	6,473,183	0.32	3.20E-07			0.24	1.20E-05	*PM05G07220*	PmChr05 6,472,971–6,476,956 (-1)
PANEx	Proso.11_24126769	11	24,126,769	17.56	5.40E-08	13.34	2.00E-05			*PM11G11600*	PmChr11 24,125,717–24,127,809 (-1)
PEDL	Proso.5_9265830	5	9,265,830	23.72	4.30E-05			24.79	1.10E-05	not on gene	
INF.PBN	Proso.7_1535098	7	1,535,098	-1.92	5.10E-20			-1.97	1.60E-19	*TE347748*	PmChr07 1,535,090–1,535,203 (-1)
INF.PBN	Proso.16_1877646	16	1,877,646	-0.45	3.50E-05			-0.84	1.60E-10	not on gene	
INF.PBN	Proso.5_15388933	5	15,388,933	-0.65	4.90E-08			-0.48	2.40E-05	*Denovo_TE004252*	PmChr05 15,388,724–15,389,433 (1)
INF.PBN	Proso.9_39804416	9	39,804,416			0.93	9.40E-07	0.93	1.60E-06	*Denovo_TE154845*	Chr PmChr09 39,802,033–39,806,828 (-1)
INFL	Proso.2_2092993	2	2,092,993	-22.21	1.90E-07			-17.46	1.20E-06	not on gene	
INFL	Proso.17_3253916	17	3,253,916	15.1	4.90E-06	11.29	8.30E-05			*PM17G03120*	PmChr17 3,252,571–3,255,649 (-1)
INFL	Proso.1_8346815	1	8,346,815	54.57	4.40E-08	32.76	1.50E-05	50.59	5.10E-07	*PM01G10560*	PmChr01 8,343,429–8,346,938 (1)
INFL	Proso.14_27820106	14	27,820,106	18.33	2.70E-05	20.37	4.40E-07	17.72	4.90E-05	*PM14G15020*	PmChr14 27,818,540–27,821,281 (-1)
INFL	Proso.12_34047515	12	34,047,515	-20.85	2.20E-09	-19.12	1.10E-09	-27.48	2.00E-14	*PM12G21200*	PmChr12 34,040,158–34,055,027 (-1)
INFL	Proso.9_39130372	9	39,130,372	-32.21	5.20E-10			-28.84	3.00E-09	not on gene	
INFL	Proso.12_41890075	12	41,890,075	31.95	2.90E-07	38.06	2.70E-10	43.61	4.50E-11	not on gene	
DM	Proso.14_29825523	14	29,825,523			1.26	3.30E-05	1.42	1.80E-06	not on gene	
GYKH	Proso.9_23681963	9	23,681,963	-305.78	2.40E-08			-316.15	3.40E-11	not on gene	
HSW	Proso.12_304735	12	304,735			0.03	2.90E-05	0.04	2.80E-07	not on gene	
HSW	Proso.9_22773800	9	22,773,800			-0.01	3.10E-05	-0.03	8.70E-10	not on gene	
Protein	Proso.10_21439333	10	21,439,333			-0.62	1.90E-05	-0.53	5.80E-06	not on gene	
Protein	Proso.14_26492521	14	26,492,521	-0.4	2.00E-05			-0.49	8.00E-06	not on gene	
Protein	Proso.1_61603598	1	61,603,598			0.42	9.60E-06	0.41	1.30E-06	not on gene	
Zn	Proso.17_30948407	17	30,948,407	1.01	7.10E-05			1.3	2.00E-06	*PM17G09880*	PmChr17 30,907,001–30,949,614 (-1)
Fe	Proso.17_5885921	17	5,885,921			2.49	1.90E-08	1.6	4.80E-05	*TE311547*	PmChr17 5,885,645–5,886,169 (1)
Fe	Proso.9_17145336	9	17,145,336	2.7	3.10E-07			2.02	1.20E-06	not on gene	

^#^ BT = Basal tillers number; DF = Days to 50% flowering; PLHT – Plant height (cm), FLBL = Flag leaf blade length (mm); Nnode = Number of nodes on main tiller; PANEx = Panicle exsertion (mm); INF- PBN = Inflorescene primary branch number; INFL = Inflorescene length (mm); DM = Days to maturity; GYKH = Grain yield (kg/ha); HSW = 100 seed weight (g).

### Comparative genomics

Twenty MTAs that were significantly associated with various traits were located within the genes. Although these specific SNPs are probably not causal polymorphisms for these traits, the rapid LD decay in this population (Fig. 6) implies that the genes have a strong probability of being involved. The sequence information of these genes was retrieved from www.genomeevolution.com and compared with related species to check the similarity and gene function. More than 90% similarity was considered to report the genes and their functions in related species (Supplementary Table 2). For example, the SNP Proso.2_14901071 associated with basal tiller number is located on the gene *PM02G15460*, showing over 90% similarity with genes in the three species, *Panicum hallii, Panicum virgatum, and Setaria italica*, with gene functions of “putative leucine-rich repeat-containing protein, sporulation-specific protein 15-like, and girdin-like”. The SNP Proso.17_3253916 associated with inflorescene length is located on the gene *PM17G03120* showed over 90% similarity with genes in the closely related species namely *Panicum hallii, Panicum virgatum, Sateria italica,* with gene function of “filament-like plant protein”. The SNP Proso.14_27820106, which is associated with inflorescene length, is located in the gene *PM14G15020* and showed over 90% similarity with *Panicum hallii, Panicum virgatum, Sateria viridis, Setaria italica, and Zea mays* genes with the gene function of “dihydrolipoyllysine-residue acetyltransferase component 4 of the pyruvate dehydrogenase complex, chloroplastic-like” (Supplementary Table 2).

## DISCUSSION

The proso millet diversity panel used in this study had an average MRD of 0.268, which varied from 0.126 to 0.341. Among the five races of proso millet, which are primarily classified on the basis of panicle morphology and shape^14^, accessions belonging to *milliaceum* had the highest average distance (0.274), whereas accessions of *ovatum* showed the lowest average distance (0.201). The lowest between-race distance was found between *the ovatum* with *compactum* (0.248) and the *ovatum* with *contractum* (0.250). Similar results were found when the same panel and the entire proso millet collection were assessed for phenotypic diversity^13,26^. The three races, namely *contractum*, *compactum*, and *ovatum,* look similar, except for panicle morphology*:* compact and drooping inflorescence in *contractum*, cylindrical and erect inflorescence in *compactum*, and compact and slightly curved inflorescence in *ovatum*^13,14^. These three races phenotypically differ from the other two races, *milliaceum* and *patentissimum*, which are often difficult to distinguish. Accessions belonging to the race *miliaceum* are characterized by a large open inflorescence with suberect branches that are sparingly subdivided, whereas those belonging to *the patentissimum* are characterized by slender and diffused panicle branches.

Understanding the diversity and population structure of germplasm resources is important for their use in crop improvement programs. Population structure analysis revealed the presence of five populations in the proso millet diversity panel, which did not correspond with these race designations. Instead, the populations corresponded well with geography. Four of the populations consisted almost entirely of Asian accessions, indicating greater genetic diversity, whereas almost all the European accessions clustered into a single population. These results support that Asia is the centre of origin and diversity of proso millet, followed by a spread westward across Europe^3,13,26^. In our previous study on the same subset, diversity and population structure were estimated using morpho-agronomic data, indicating that the accessions of proso millet were structured largely according to geographical region. Accessions originating in Asia and Europe were distinctly grouped, also accessions from Asia showed high diversity (average distance 0.268) relative to those from Europe (average distance 0.225), and high diversity was observed between accessions of Asia and Europe (average distance 0.301)^26^.

**Fig. 8:**
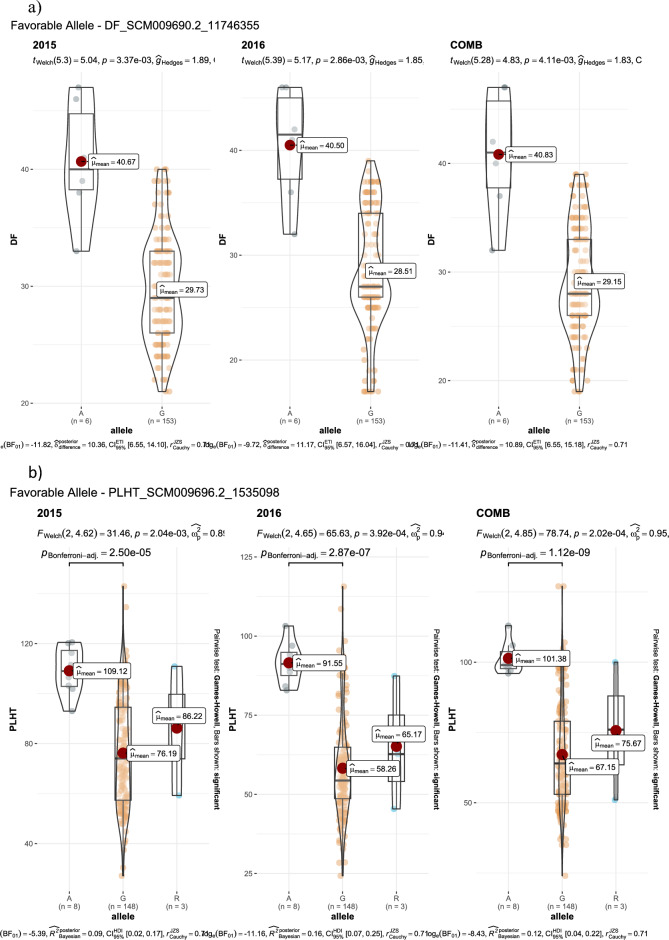

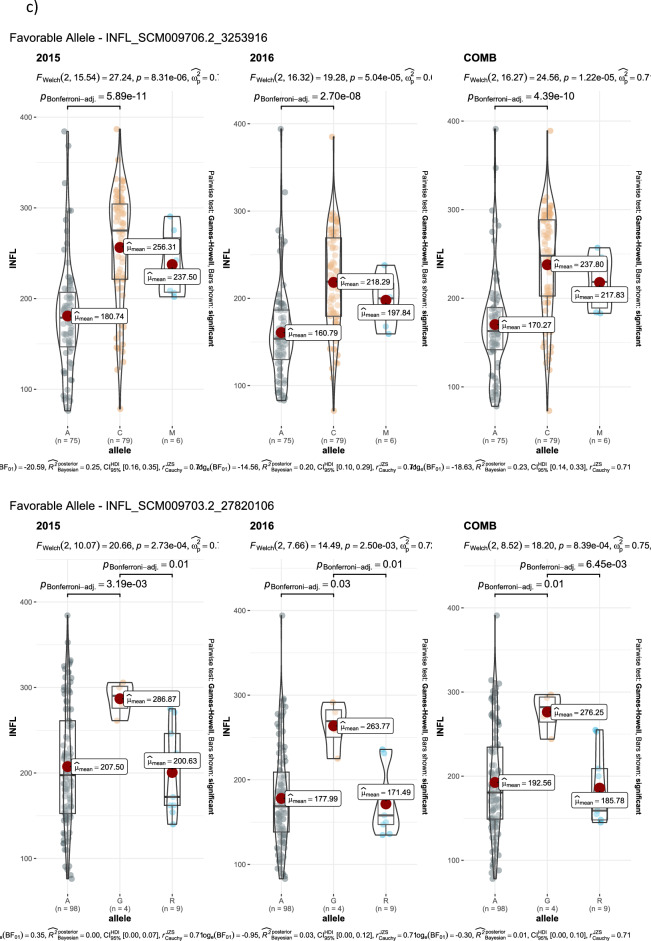

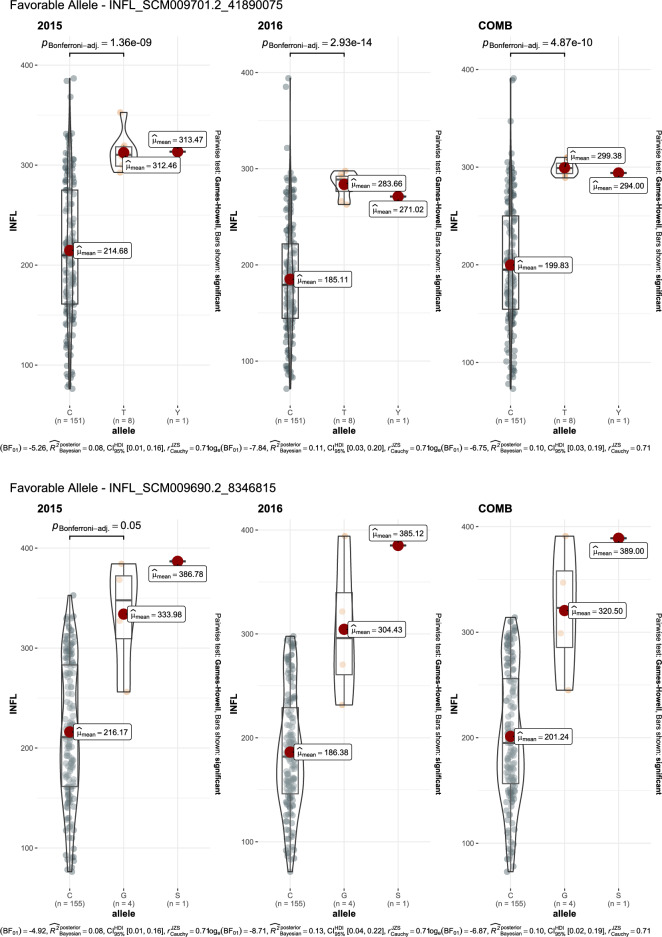

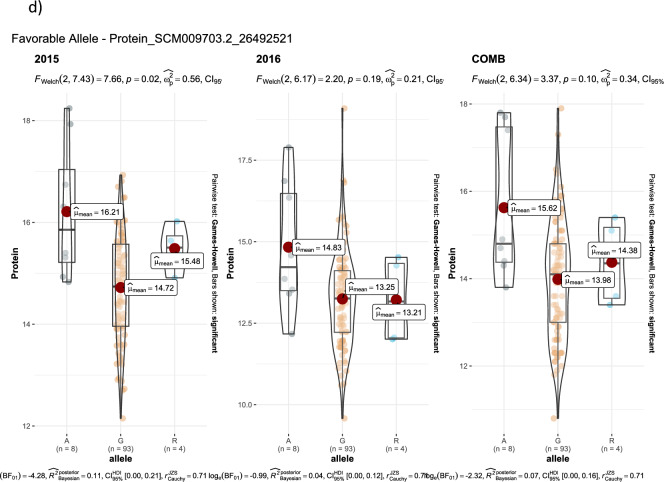
The MTAs with alleles and trait values, for use in genomic assisted improvement of proso millet (Note: SNP name starts with the code DF = Days to 50% flowering; PLHT = Plant height, cm; INFL = Inflorescence length, mm; Protein %).

Advances in NGS technologies and the availability of a draft genome for proso millet can accelerate genomics-assisted crop improvement^16,17^. In proso millet, Rajput et al.^28^ reported QTLs for morpho-agronomic traits using bi-parental mapping, while there are only two reports available on GWAS for agronomic and seed traits^24,25^, and no report on grain nutrients. In this study, a diversity set of proso millets representing five races originating from 30 countries was genotyped using the GBS approach. After filtering, 160 accessions originating from 26 countries and 5,621 quality SNPs were used to perform GWAS on agronomic and grain nutrient traits. A total of 40 MTAs were identified: 34 for agronomic traits and six for grain nutrient traits. Nine MTAs (two for flag leaf blade length, five for inflorescence length, and one each for plant height and paniel exsertion) were identified as linked with the phenotypic trait of variation in both years, and five of them showed significant associations in both the years as well as when the years were combined. Long inflorescences and tall plants are among the important traits that are positively associated with higher grain yield in proso millet^26^. Of the seven SNPs that were associated with inflorescence length, four were associated in both years as well as in the combined data. Among these, four SNPs showed a positive effect (Proso.1_8346815, Proso.12_41890075, Proso.14_27820106, and Proso.17_3253916), whereas Proso.12_34047515 and Proso.9_39130372 had a negative effect on inflorescence length. The four SNPs, Proso.17_3253916, Proso.1_8346815, Proso.14_27820106, and Proso.12_34047515, are located on genes PM17G03120, *PM01G10560*, *PM14G15020*, and *PM12G21200*, respectively, indicating potential candidate genes for yield improvement in proso millet. For plant height, two SNPs were identified, located on chromosomes 7 and 9. The SNP Proso.7_1535098 showed significant association in both years as well as in the combined data, and is located in the gene TE347748 with gene function of “putative pentatricopeptide repeat-containing protein” and “ninja-family protein 8-like” (Supplementary Table 2). Six SNPs were identified for grain nutrient content, of which two were located in the genes. For all the MTAs identified in this study, a box plot showing alleles and their phenotypic values was estimated, which is important for further use of these SNPs in the genomics-assisted improvement of traits (Fig. 8, Supplementary Fig. 2). Sequence similarity of the identified genes was compared with related species, and gene functions were reported, which will help in understanding the genetic basis of phenotypic variation of different traits.

In conclusion, proso millet is a potential crop for food security and nutrition and has various climate resilience and nutritional benefits. However, systematic breeding and genomics-assisted improvements in proso millet are very limited. In this study, the NGS-based genotypic characterization of proso millet revealed a wider diversity within and among races, and proso millet germplasm diversity was structured according to the two geographical regions where proso millet was reported to originate and be domesticated. Genome-wide association mapping identified 40 marker–trait associations for agronomic traits (34) and grain nutrients (6), most of which were located within the genes. The information generated from this study on diversity and marker-trait associations can support the development of allele-specific markers for mining productivity and nutrient traits, and their utilization in genomic-assisted proso millet improvement.

## Materials and methods

### Phenotyping

#### Experimental materials and conditions

The experimental material consisted of 200 accessions which includes the core collection (106 accessions)^29^. Number of accessions in the core collection is less for GWAS therefore we followed same approach by which core collection was established to make a diversity subset of 200 accessions from the entire collection of 849 accessions conserved in the genebank (http://genebank.icrisat.org/). These accessions were planted during the 2015 and 2016 rainy seasons at ICRISAT (Patancheru, Telangana, India; 17° 30′ N latitude, 78° 15′ E longitude and altitude 545 MSL) in an alpha design with two replications on red soil, planted in the third week of July during both years. Sowings were performed on ridges 60 cm apart, and each accession occupied a single row of 4 m in length. Plant-to-plant spacing of approximately 10 cm was maintained by thinning the excess seedlings. Diammonium phosphate was applied at a rate of 100 kg/ha as a basal dose to supply nitrogen and phosphorus. In addition, 100 kg/ha of urea was applied as top dressing. Irrigation and hand-weeding were performed on a need-based basis.

### Data collection

Data on 14 agronomic traits were recorded using the descriptors of *Panicum miliaceum*^30^. The agronomic traits of days to 50% flowering, days to maturity, and grain yields were recorded on a plot basis, while the other agronomic traits (plant height, basal tillers, flag leaf blade length, flag leaf blade width, flag leaf sheath length, peduncle length, panicle extension, Inflorescence length, number of nodes, and inflorescence primary branch number) were recorded on the main culms of the five representative plants in a plot. Bulked seeds of each accession were used to determine the 100-seed weight. The grain yield per plot was converted into grain yield (kg/ha). A random, well-cleaned grain sample (unhusked) from each accession was used to estimate the grain protein, calcium (Ca), iron (Fe), and zinc (Zn) content at the Charles Renard Analytical Laboratory, ICRISAT, Patancheru, India. Grain Ca, Fe, and Zn contents were assessed following the nitric acid–hydrogen peroxide digestion method, and Ca, Fe, and Zn in the digests were analyzed using inductively coupled plasma-optical emission spectrometry (ICP-OES)^31^. Protein content in grain samples was determined using the sulfuric acid–selenium digestion method. Total nitrogen (N) was estimated using a Skalar Autoanalyzer, and protein % was calculated as N% × 6.25 conversion factor^32^.

### Phenotypic data analysis

Data were analyzed for each rainy season separately and pooled following Residual Maximum Likelihood (REML)^33^ in GenStat, 17th edition (http://www.genstat.co.uk) considering genotypes as random and seasons as fixed effects. The significance of seasons was tested using Wald’s statistics^34^. The Best Linear Unbiased Predictors (BLUPs) were obtained for all the traits for each accession for individual seasons, pooled over two rainy seasons, and used for genome-wide association studies.

### Genotyping and SNP calling

DNA extraction and SNP calling from genotyping-by-sequencing (GBS) data have been described in detail in our previous publication^35^. In brief, DNA was extracted from each accession following the modified CTAB method^36^, lyophilized, and shipped to the Genomic Diversity Facility at Cornell University for GBS^37^. GBS library preparation followed the standard method^38^ using a single *Pst*I restriction enzyme. Samples were multiplexed into two lanes of 95 samples plus one blank for sequencing on an Illumina HiSeq 2500 with single-end 100 bp sequencing. The sequences were mapped to proso millet reference genome *Panicum miliaceum* (vPm_0390_v1) (https://andgenomevolution.org/coge/SearchResults.pl?s=52484&p=genome)^17^ using Bowtie v2.2.4^39^. SNPs were called using the GBS v2 pipeline in TASSEL v4.3.6. Raw SNPs were filtered by removing any sites with greater than 20% missing, less than 0.1 proportion heterozygous, and a minor allele frequency of < 0.025. Accessions with more than 20% of their missing sites were filtered out. This resulted in 5621 high-confidence SNPs that were used for GWAS.

### Population structure and genetic distance

AMOVA was computed to determine the presence of significant variation in the collection and assess the contribution of different stratifications to diversity. Principal Component Analysis was used to summarize and obtain preliminary knowledge about the diversity within the collection. The hierarchical population structure was estimated using the ADMIXTURE program, which is a model-based estimation of ancestry in unrelated individuals using the maximum-likelihood method^40^. ADMIXTURE implements a cross-validation (CV) feature that allows, together with the number of iterations to converge, the determination of the number of subpopulations (k values) that best fit the data. After choosing the subpopulation level, individual accessions were assigned to the subpopulation if they had at least 60% membership in that respective population^41^. We calculated the Modified Roger’s Distances (MRD) between samples^42,43^ as,

$$MRD=\sqrt{\frac{1}{2m}\sum_{i=1}^{m}\sum_{j=1}^{{a}_{i}}{({p}_{ij}-{q}_{ij})}^{2}}$$

where ${p}_{ij}$ and ${q}_{ij}$ are the allele frequencies of jth and ith markers in the two samples under consideration, ${a}_{i}$ is the number of alleles in the ith marker and $m$ refers to the number of markers. Clustering of accessions based on the MRD distances was performed using Ward’s D2 hierarchical clustering algorithm^44^.

### Linkage disequilibrium (LD) and genome-wide association mapping

TASSEL 4.0 was used to obtain the LD squared allele frequency correlation (r^2^) estimates for all pairwise comparisons between intra- and whole-genome SNPs, and visualized by plotting r^2^ values against physical distance. A non-linear regression curve was used to estimate LD decay^45^ using R^46^. The LD decay distance was estimated as the physical distance at which r^2^ was reduced to half the maximum LD value.

Genome-wide association analysis was performed using a multi-locus model, FarmCPU^47^. FarmCPU iteratively used the fixed-effect and random-effect models, and significant marker-trait associations (P ≤ 0.0001) were identified. GWAS was performed using the BLUPs of each trait obtained from individual years separately and combined across two years. Markers that showed significant associations with the trait of interest in at least two out of the three datasets (2015, 2016, and combined) were considered for SNP annotation and candidate gene identification using the comparative genome database (https://genomevolution.org/coge/) and the ID52484 and vPm_0390_v1 of *Panicum miliaceum*^17^.

## Supplementary Information

Supplementary Information.

## Data Availability

The raw sequence data is on NCBI’s Sequence Read Archive under accession PRJNA494158. The filtered SNPs and Phenotypic data were deposited to Figshare repository, 10.6084/m9.figshare.26199836.v1. All other supporting data are provided in the Supplementary files.
