# Supplementary material for: Genome-wide assessment of population structure and association mapping for agronomic and grain nutritional traits in proso millet (Panicum miliaceum L.)
Source: Sci Rep. 2024 Sep 19;14:21920. doi: 10.1038/s41598-024-72319-w (PMC11413307; doi:10.1038/s41598-024-72319-w)
Supplement: Supplementary file 1 — Supplementary Information. [file 41598_2024_72319_MOESM1_ESM.pdf]

**Supplementary Table 1.** Modified Roger's Distance based on GBS-based SNP characterization of proso millet germplasm

| | Modified Roger's Distance | | |
|---|---|---|---|
| Race | Mean | Range | SD |
| Entire set | 0.268 | 0.126-0.341 | 0.04 |
| **Within races** | | | |
| *compactum* | 0.254 | 0.157-0.330 | 0.06 |
| *contractum* | 0.264 | 0.181-0.324 | 0.07 |
| *miliaceum* | 0.274 | 0.138-0.341 | 0.05 |
| *ovatum* | 0.201 | 0.126-0.274 | 0.07 |
| *patentissimum* | 0.264 | 0.171-0.308 | 0.08 |
| **Between races** | | | |
| *contractum* and compactum | 0.258 | 0.140-0.330 | 0.04 |
| *miliaceum* and *compactum* | 0.267 | 0.147-0.339 | 0.04 |
| *miliaceum* and *contractum* | 0.269 | 0.136-0.332 | 0.04 |
| *ovatum* and *compactum* | 0.248 | 0.166-0.330 | 0.03 |
| *ovatum* and *contractum* | 0.250 | 0.161-0.322 | 0.04 |
| *ovatum* and *miliaceum* | 0.260 | 0.141-0.336 | 0.04 |
| *patentissimum* and *compactum* | 0.270 | 0.165-0.337 | 0.04 |
| *patentissimum* and *contractum* | 0.270 | 0.166-0.329 | 0.04 |
| *patentissimum* and *miliaceum* | 0.272 | 0.163-0.339 | 0.04 |
| *patentissimum* and *ovatum* | 0.260 | 0.198-0.313 | 0.04 |

**Supplementary Table 2.** Sequence similarity and genome annotation of proso millet genes on which the significant SNPs were located, with the related species

| Proso millet | | | | Gene similarity with related crops | | | | | |
|---|---|---|---|---|---|---|---|---|---|
| **SNP** | **Chromosome** | **Position** | **Gene Id** | **Species** | **Sequence similarity (%)** | **Chromosome** | **Position** | **Gene ID** | **Annotation** |
| **Basal tillers number** | | | | | | | | | |
| Proso.2_14901071 | 2 | 14901071 | *PM02G15460* | *Panicum hallii* | 99.3 | 9 | 19161911..19172814 | LOC112873779 (GeneID: 112873779) | putative leucine-rich repeat-containing protein DDB_G0290503 |
| | | | | *Panicum virgatum* | 99.2 | 9N | 39470356..39481606 | LOC120693262 (GeneID: 120693262) | sporulation-specific protein 15-like |
| | | | | *Panicum virgatum* | 95.6 | 9K | 31198186..31210438 | LOC120651238 (GeneID: 120651238) | girdin-like |
| | | | | *Setaria italica* | 92.5 | 9 | 23394349..23406288 | LOC101774308 (GeneID:101774308) | putative leucine-rich repeat-containing protein DDB_G0290503 |
| | | | | *Setaria italica* | 95.4 | 9 | 23263600..23275652 | LOC117837246 (GeneID:117837246) | putative leucine-rich repeat-containing protein DDB_G0290503 |
| Proso.12_28525796 | 12 | 28525796 | *PM12G15280* | *Panicum hallii* | 99.1 | 1 | 44355473..44359289 | LOC112885898 (GeneID:112885898) | J protein JJJ2-like |
| | | | | *Panicum virgatum* | 95.4 | 1K | 30638656..30642425 | LOC120702446 (GeneID:120702446) | uncharacterized protein |
| | | | | *Panicum virgatum* | 95.5 | 1N | 40405605..40409363 | LOC120655828 (GeneID:120655828) | uncharacterized protein |
| | | | | *Setaria italica* | 94.7 | 1 | 24262288..24266226 | LOC101777479 (GeneID:101777479) | uncharacterized protein |
| | | | | *Setaria italica* | 94.7 | 1 | 24290739..24294663 | LOC117852048 (GeneID:117852048) | uncharacterized protein |
| **Flag leaf blade length (mm)** | | | | | | | | | |
| Proso.8_9836654 | 8 | 9836654 | *PM08G11490* | *Panicum hallii* | 99.3 | 5 | 48755073..48761732 | LOC112895059 (GeneID:112895059) | dual specificity protein phosphatase PHS1-like |
| | | | | *Panicum virgatum* | 96.3 | 5N | 18795912..18802728 | LOC120673779 (GeneID:120673779) | dual specificity protein phosphatase PHS1-like |
| | | | | *Panicum virgatum* | 96.1 | 5K | 17498941..17505901) | LOC120706723 (GeneID:120706723) | dual specificity protein phosphatase PHS1-like |
| | | | | *Setaria viridis* | 94.8 | 5 | 1521475..1528937 | LOC117857623 (GeneID:117857623) | dual specificity protein phosphatase PHS1-like |

| Proso millet | | | | Gene similarity with related crops | | | | | |
|---|---|---|---|---|---|---|---|---|---|
| **SNP** | **Chromosome** | **Position** | **Gene Id** | **Species** | **Sequence similarity (%)** | **Chromosome** | **Position** | **Gene ID** | **Annotation** |
| | | | | *Setaria italica* | 94.8 | 5 | 2339394..2346843 | LOC101781896 (GeneID:101781896) | dual specificity protein phosphatase PHS1 |
| **Flag leaf sheath length (mm)** | | | | | | | | | |
| Proso.7_1535098 | 7 | 1535098 | *TE347748* | *Panicum hallii* | 91.8 | 3 | 5354517..5357511 | LOC112884697 (GeneID:112884697) | putative pentatricopeptide repeat-containing protein |
| Proso.12_34047515 | 12 | 34047515 | *PM12G21200* | *Panicum hallii* | 98.8 | 1 | 50476843..50492028 | LOC112876342 (GeneID:112876342) | uncharacterized protein |
| | | | | *Panicum virgatum* | 96.8 | 1N | 51974275..51989155 | LOC120656545 (GeneID:120656545) | uncharacterized protein |
| | | | | *Panicum virgatum* | 96.7 | 1K | (29612939..29637255) | LOC120702044 (GeneID:120702044) | uncharacterized protein |
| | | | | *Panicum virgatum* | 94.7 | 1N | 37533640..37562497 | LOC120655754 (GeneID:120655754) | uncharacterized protein |
| | | | | *Setaria italica* | 94.6 | 1 | 32177918..32196229 | LOC101760594 (GeneID:101760594) | uncharacterized protein |
| | | | | *Setaria italica* | 94.5 | 1 | 31887757..31905989 | LOC117863376 (GeneID:117863376) | uncharacterized protein |
| **Inflorescene primary branch number** | | | | | | | | | |
| Proso.7_1535098 | 7 | 1535098 | *TE347748* | *Panicum hallii* | 91.8 | 3 | 5354517..5357511 | LOC112884697 (GeneID:112884697) | putative pentatricopeptide repeat-containing protein |
| **Inflorescene length (mm)** | | | | | | | | | |
| Proso.17_3253916 | 17 | 3253916 | *PM17G03120* | *Panicum hallii* | 96.3 | 8 | 3300468..3307921 | LOC112903408 (GeneID:112903408) | filament-like plant protein |
| | | | | *Panicum virgatum* | 95.6 | 8K | 6557826..6565360 | LOC120643737 (GeneID:120643737) | filament-like plant protein 3 |
| | | | | *Panicum virgatum* | 95.6 | 8N | 5078635..5085274 | LOC120684615 (GeneID:120684615) | filament-like plant protein 3 |
| | | | | *Setaria italica* | 92.5 | 8 | 4105301..4118361 | LOC117833294 (GeneID:117833294) | filament-like plant protein 6 |
| Proso.1_8346815 | 1 | 8346815 | *PM01G10560* | *Panicum hallii* | 98.3 | 9 | 65716242..65720579 | LOC112875768 (GeneID:112875768) | uncharacterized protein |
| | | | | *Panicum virgatum* | 97.4 | 9N | 69941043..69945540 | LOC120691663 (GeneID:120691663) | uncharacterized protein |
| | | | | *Panicum virgatum* | 96.6 | 9K | 59670013..59675287 | LOC120652873 (GeneID:120652873) | uncharacterized protein |
| | | | | *Setaria viridis* | 95.1 | 9 | 48223629..48229686 | LOC117839717 (GeneID:117839717) | uncharacterized protein |

| Proso millet | | | | Gene similarity with related crops | | | | | |
|---|---|---|---|---|---|---|---|---|---|
| **SNP** | **Chromosome** | **Position** | **Gene Id** | **Species** | **Sequence similarity (%)** | **Chromosome** | **Position** | **Gene ID** | **Annotation** |
| | | | | *Setaria italica* | 95.0 | 9 | 50617083..50623029 | LOC101767048 (GeneID:101767048) | uncharacterized protein |
| | | | | *Zea mays* | 91.4 | 1 | 39163896..39170574 | LOC103634306 (GeneID:103634306) | fringe-related protein |
| | | | | *Sorghum bicolor* | 89.6 | 1 | 70421838..70427369 | LOC8081520 (GeneID:8081520) | uncharacterized protein |
| Proso.14_27820106 | 14 | 27820106 | *PM14G15020* | *Panicum hallii* | 98.9 | 6 | 37984951..37987670 | LOC112897286 (GeneID:112897286) | dihydrolipoyllysine-residue acetyltransferase component 4 of pyruvate dehydrogenase complex, chloroplastic-like |
| | | | | *Panicum virgatum* | 96.68 | 6N | 38471815..38474555 | LOC120677768 (GeneID:120677768) | dihydrolipoyllysine-residue acetyltransferase component 4 of pyruvate dehydrogenase complex, chloroplastic-like |
| | | | | *Setaria viridis* | 95.15 | 6 | 28164111..28167017 | LOC117861256 (GeneID:117861256) | dihydrolipoyllysine-residue acetyltransferase component 4 of pyruvate dehydrogenase complex, chloroplastic-like |
| | | | | *Setaria italica* | 95.15 | 6 | 27698076..27700994 | LOC101763713 (GeneID:101763713) | dihydrolipoyllysine-residue acetyltransferase component 4 of pyruvate dehydrogenase complex, chloroplastic-like |
| | | | | *Panicum virgatum* | 95.15 | 6K | 36647363..36650123 | LOC120712422 (GeneID:120712422) | dihydrolipoyllysine-residue acetyltransferase component 4 of pyruvate dehydrogenase complex, chloroplastic-like |
| | | | | *Zea mays* | 90.74 | 1 | 219604734..219607859 | LOC100272519 (GeneID:100272519) | uncharacterized protein |
| Proso.12_34047515 | 12 | 34047515 | *PM12G21200* | *Panicum hallii* | 98.84 | 1 | 50476843..50492028 | LOC112876342 (GeneID:112876342) | uncharacterized protein |
| | | | | *Panicum virgatum* | 96.81 | 1N | 51974275..51989155 | LOC120656545 (GeneID:120656545) | uncharacterized protein |
| | | | | *Panicum virgatum* | 96.71 | 1K | 29612939..29637255 | LOC120702044 (GeneID:120702044) | uncharacterized protein |
| | | | | *Setaria italica* | 94.56 | 1 | 32177918..32196229 | LOC101760594 (GeneID:101760594) | uncharacterized protein |
| | | | | *Setaria italica* | 94.52 | 1 | 31887757..31905989 | LOC117863376 (GeneID:117863376) | uncharacterized protein |
| **Number of nodes on main tiller** | | | | | | | | | |
| Proso.5_6473183 | 5 | 6473183 | *PM05G07220* | *Panicum hallii* | 97.46 | 3 | 3192305..3196393 | LOC112885829 | histone deacetylase 14 |

| Proso millet | | | | Gene similarity with related crops | | | | | |
|---|---|---|---|---|---|---|---|---|---|
| SNP | Chromosome | Position | Gene Id | Species | Sequence similarity (%) | Chromosome | Position | Gene ID | Annotation |
| | | | | | | | | (GeneID:112885829) | |
| | | | | *Panicum virgatum* | 96.81 | 3N | 3230956..3234070 | LOC120663756 (GeneID:120663756) | cx9C motif-containing protein 4-like |
| | | | | *Panicum virgatum* | 96.14 | 3K | 3861791..3865983 | LOC120696884 (GeneID:120696884) | histone deacetylase 14-like |
| | | | | *Panicum virgatum* | 94.57 | 3K | 3859215..3861645 | LOC120696883 (GeneID:120696883) | cx9C motif-containing protein 4-like |
| | | | | *Setaria italica* | 92.96 | 7 | 32912808..32916862 | LOC101766996 (GeneID:101766996) | histone deacetylase 14 |
| | | | | *Setaria viridis* | 92.81 | 7 | 32052408..32056485 | LOC117864141 (GeneID:117864141) | histone deacetylase 14-like |
| | | | | *Sorghum bicolor* | 91.7 | 3 | 66687911..66692549 | LOC8085432 (GeneID:8085432) | histone deacetylase 14 |
| | | | | *Panicum hallii* | 95.36 | 3 | 3189129..3192173 | LOC112885830 (GeneID:112885830) | cx9C motif-containing protein 4 |
| **Panicle exsertion (mm)** | | | | | | | | | |
| Proso.11_24126769 | 11 | 24126769 | *PM11G11600* | *Panicum hallii* | 94.01 | 1 | 402096..404605 | LOC112884327 (GeneID:112884327) | RNA-binding protein 48-like |
| | | | | *Panicum virgatum* | 98.01 | 1N | 598369..600982 | LOC120654367 (GeneID:120654367) | RNA-binding protein 48-like |
| | | | | *Panicum virgatum* | 92.64 | 1K | 660614..663105 | LOC120664414 (GeneID:120664414) | RNA-binding protein 48-like |
| | | | | *Setaria italica* | 91.28 | 1 | 269539..271733 | LOC101767223 (Gene ID: 101767223) | RNA-binding protein 48-like |
| | | | | *Setaria viridis* | 91.01 | 1 | 248846..251023 | LOC117849202 (GeneID:117849202) | RNA-binding protein 48 |
| **Plant height (cm)** | | | | | | | | | |
| Proso.7_1535098 | 7 | 1535098 | *TE347748* | *Panicum hallii* | 91.76 | 3 | 5354517..5357511 | LOC112884697 (GeneID:112884697) | putative pentatricopeptide repeat-containing protein At1g53330 |
| | | | | *Panicum virgatum* | 90.77 | 3N | 16593434..16597191 | LOC120664671 (GeneID:120664671) | ninja-family protein 8-like |
| **Zn (mg/kg)** | | | | | | | | | |
| Proso.17_30948407 | 17 | 30948407 | *PM17G09880* | *Panicum virgatum* | 86.69 | 8K | 33289957..33291455 | LOC120645640 (GeneID:120645640) | uncharacterized protein |
| **Fe (mg/kg)** | | | | | | | | | |
| Proso.17_5885921 | 17 | 5885921 | *TE311547* | *Panicum virgatum* | 85.34 | 8N | 45011769..45017354 | LOC120685982 (GeneID:120685982) | uncharacterized protein |

**Supplementary Figure 1.** Frequency distribution of agronomic and grain nutrients traits of proso millet germplasm evaluated in 2015 and 2016, and the combined of both the years, at ICRISAT Patancheru, India

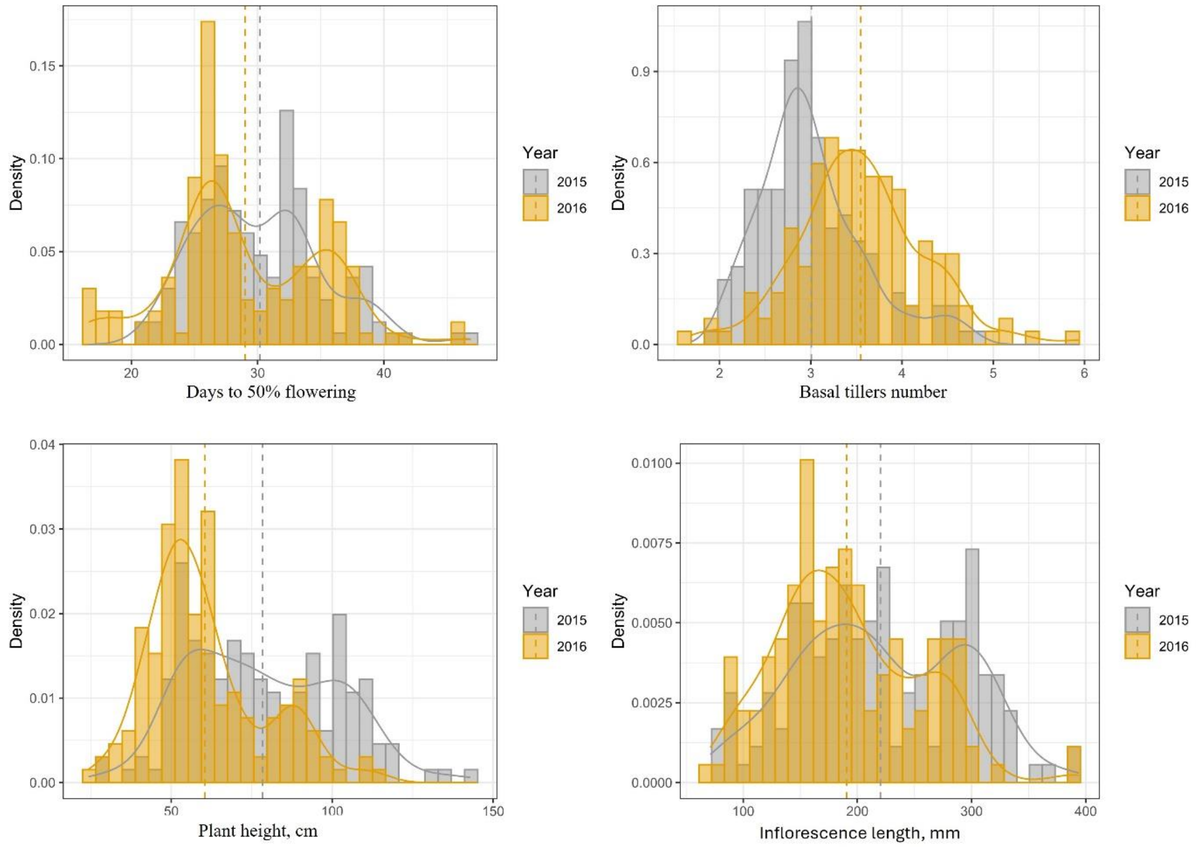

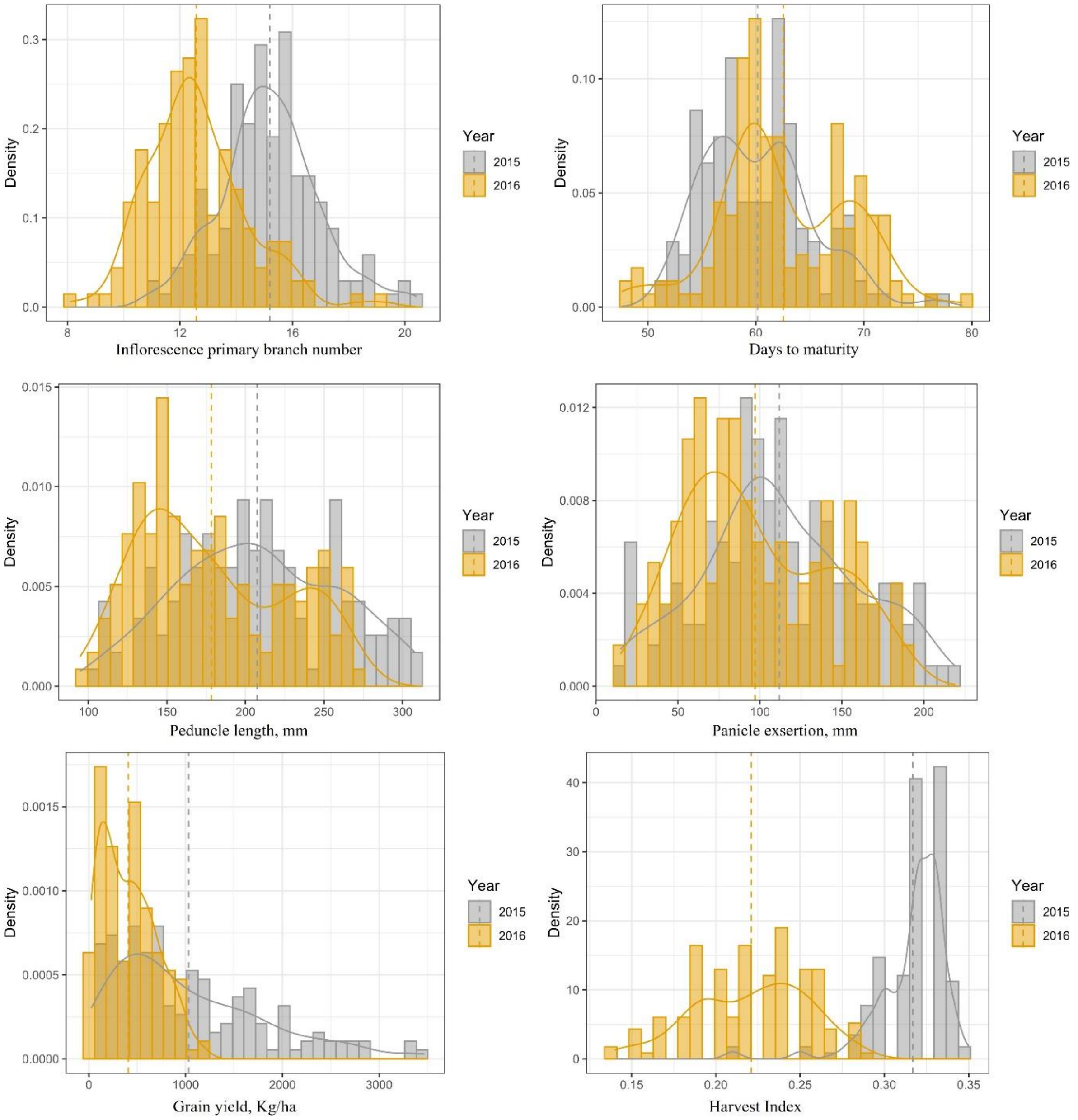
Density
Inflorescence primary branch number
Days to maturity
Peduncle length, mm
Panicle exsertion, mm
Grain yield, Kg/ha
Harvest Index
Year
2015
2016

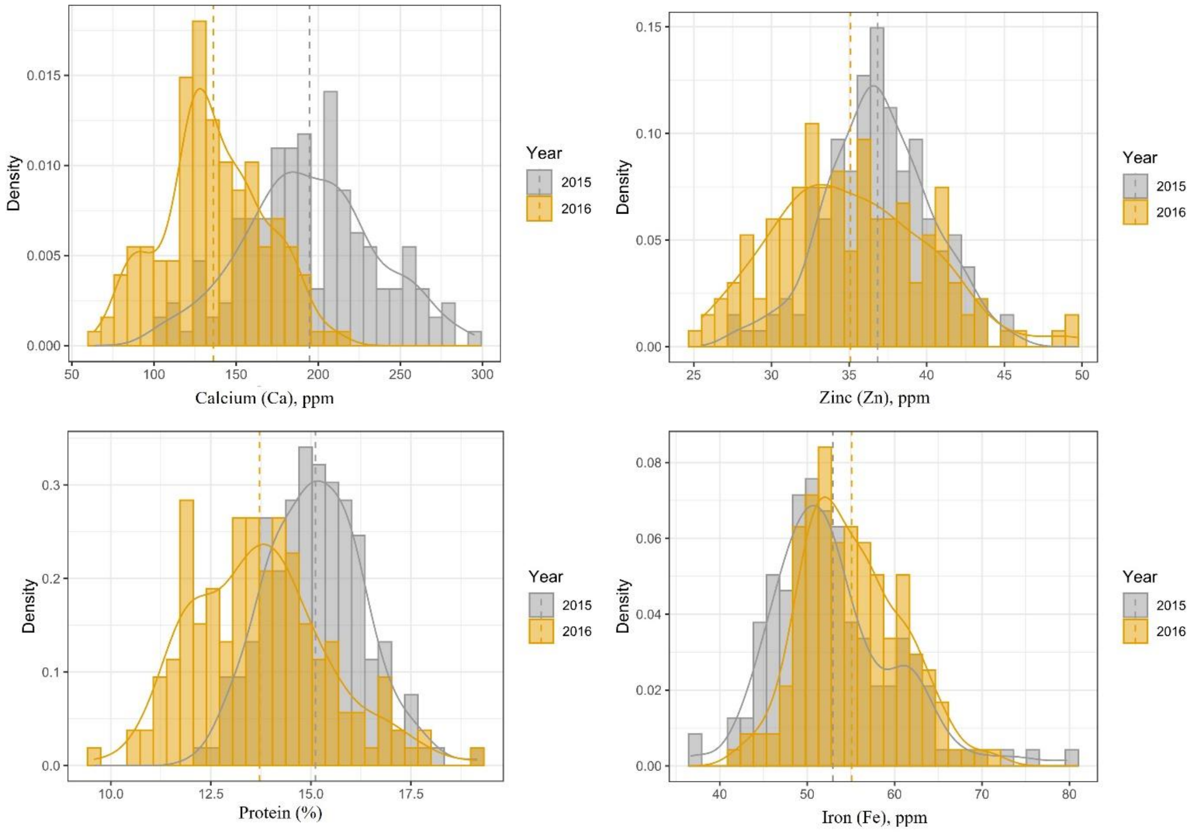
Density
Calcium (Ca), ppm
Year
2015
2016
Zinc (Zn), ppm
Protein (%)
Iron (Fe), ppm

Frequency distribution of agronomic and grain nutrients traits based on the combined data of both the years

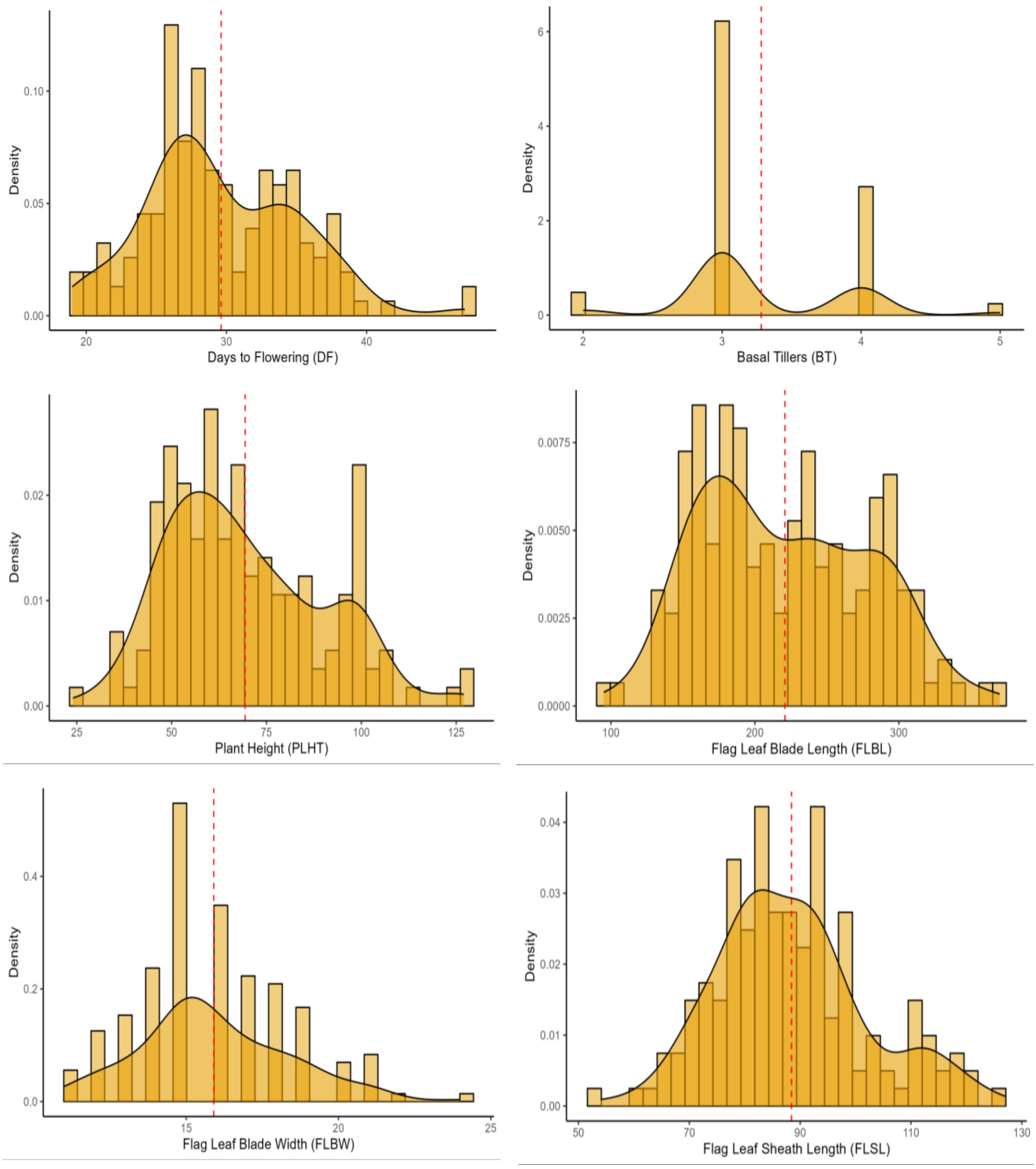

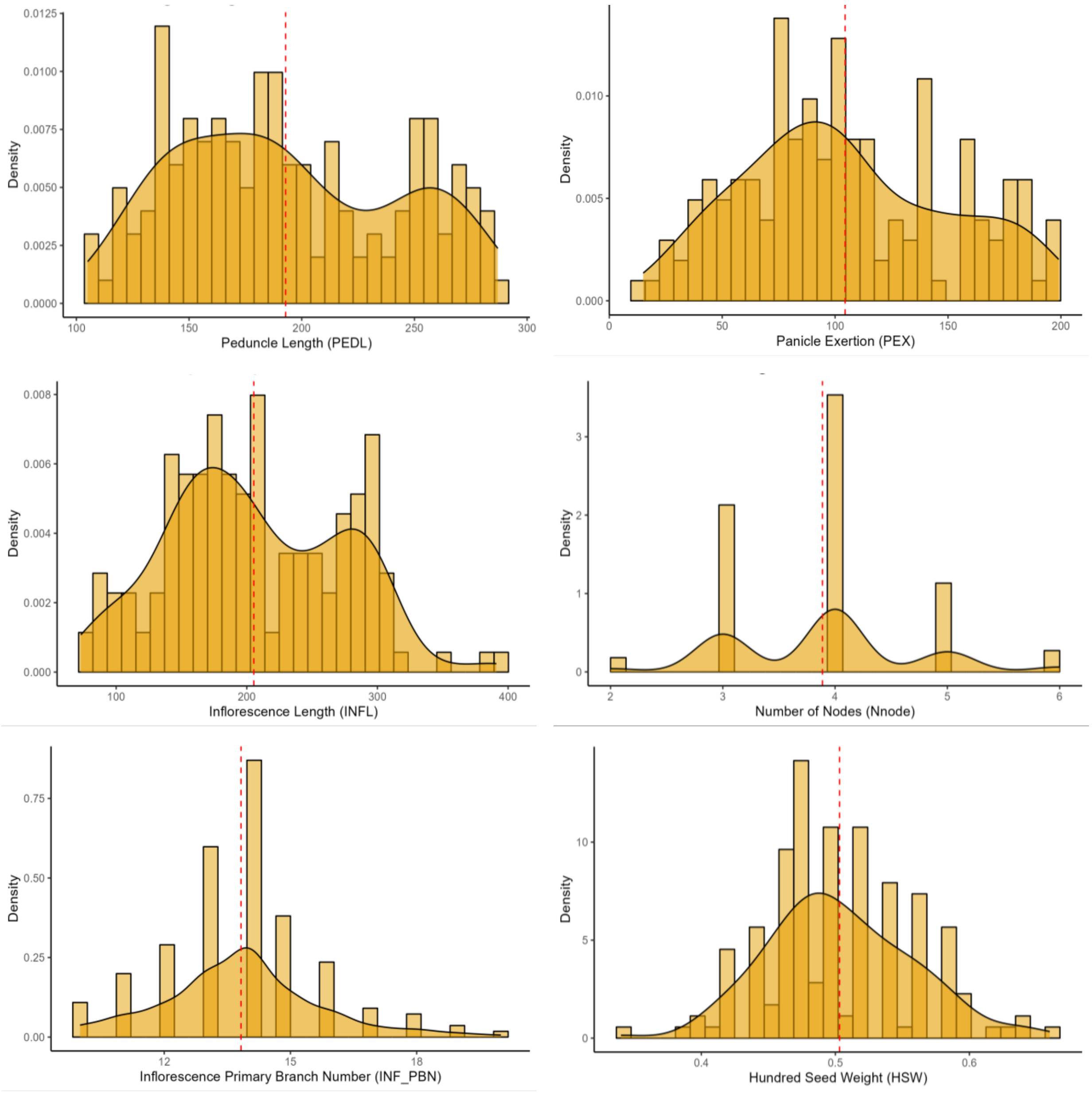
Density
Peduncle Length (PEDL)
Density
Panicle Exertion (PEX)
Density
Inflorescence Length (INFL)
Density
Number of Nodes (Nnode)
Density
Inflorescence Primary Branch Number (INF_PBN)
Density
Hundred Seed Weight (HSW)

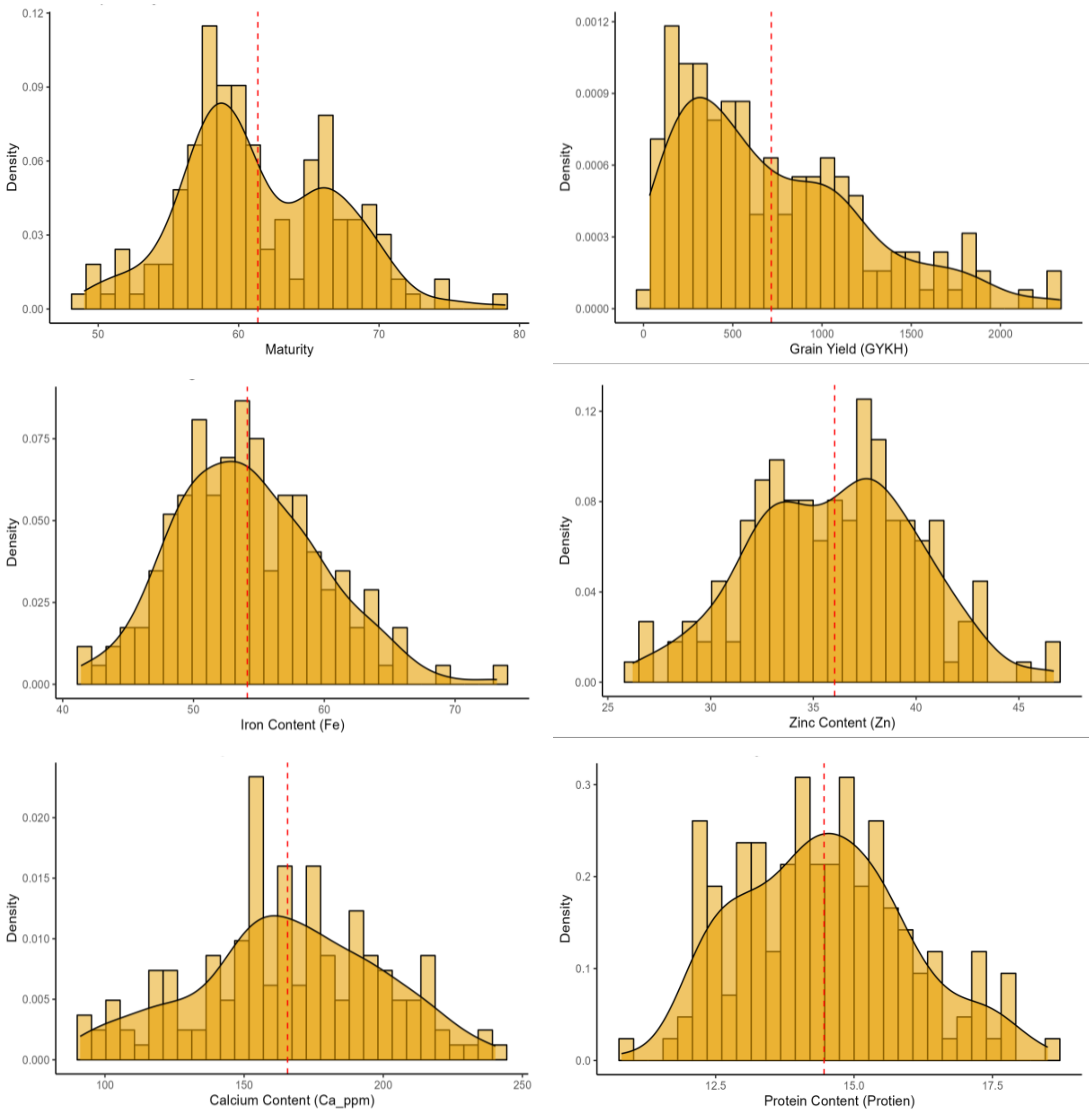
Density
Maturity
Density
Grain Yield (GYKH)
Density
Iron Content (Fe)
Density
Zinc Content (Zn)
Density
Calcium Content (Ca_ppm)
Density
Protein Content (Protien)

**Supplementary Figure 2.** Box plots showing alleles and their phenotypic values were estimated which is important for further use of those SNPs in the genomic-assisted improvement of trait

## DF= Days to 50% flowering

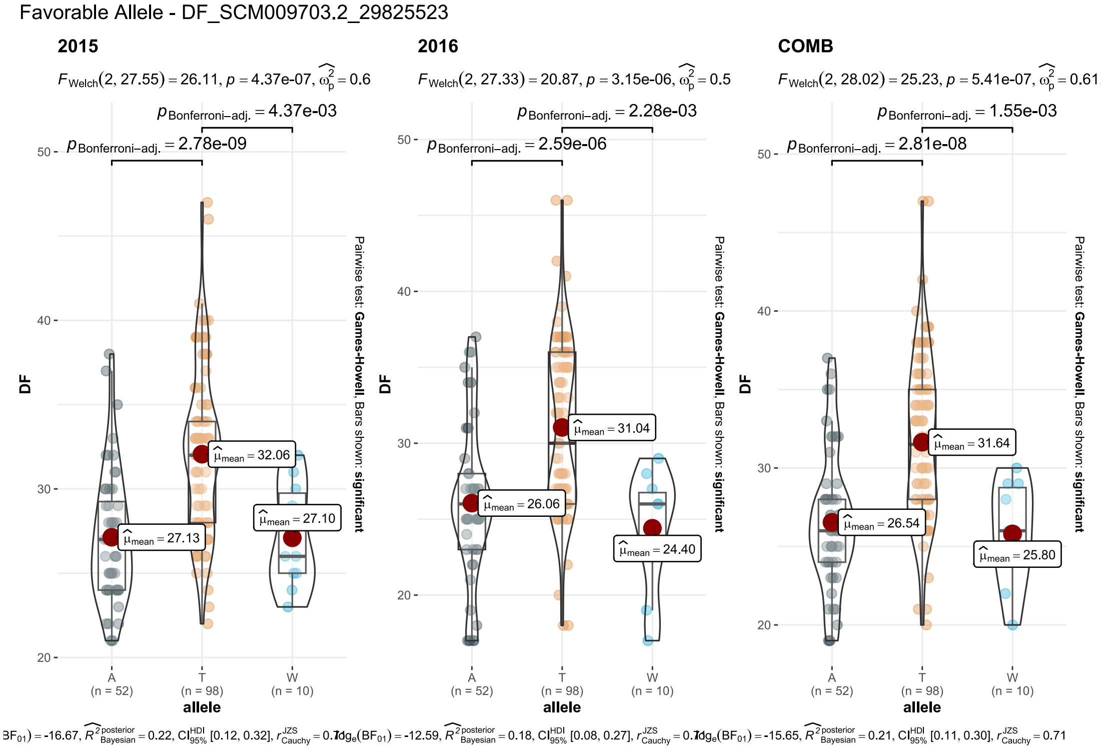


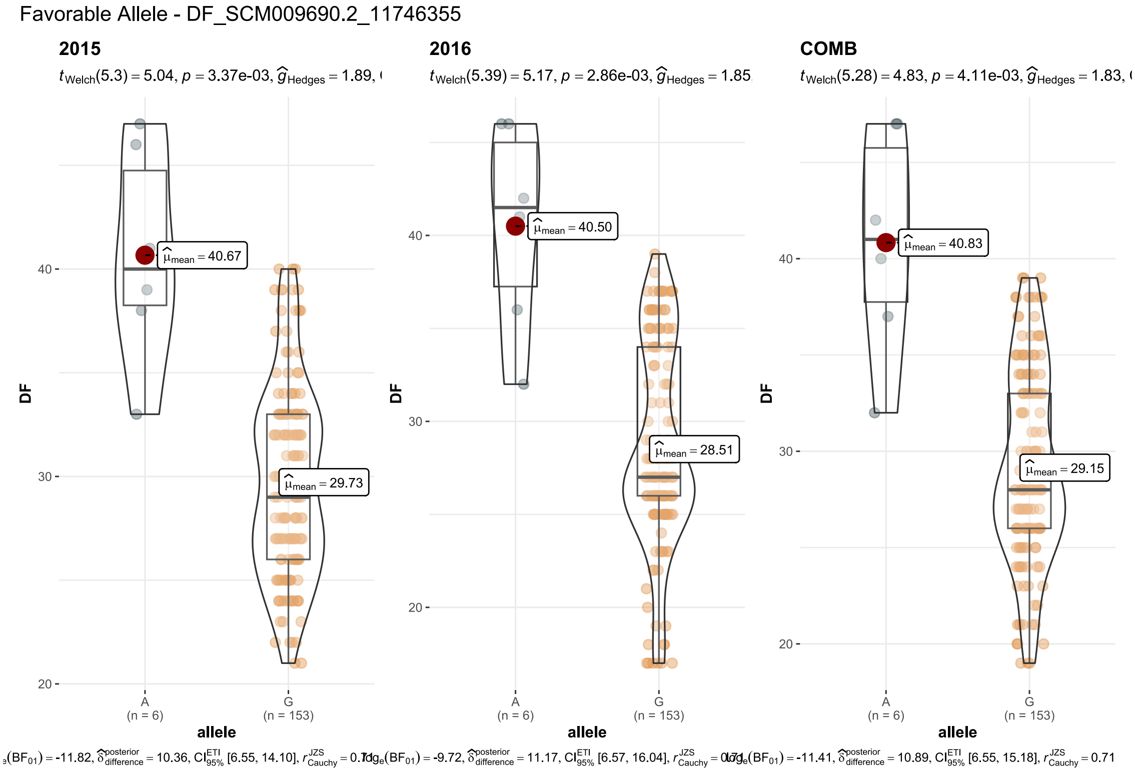

# BT=Basal tillers number

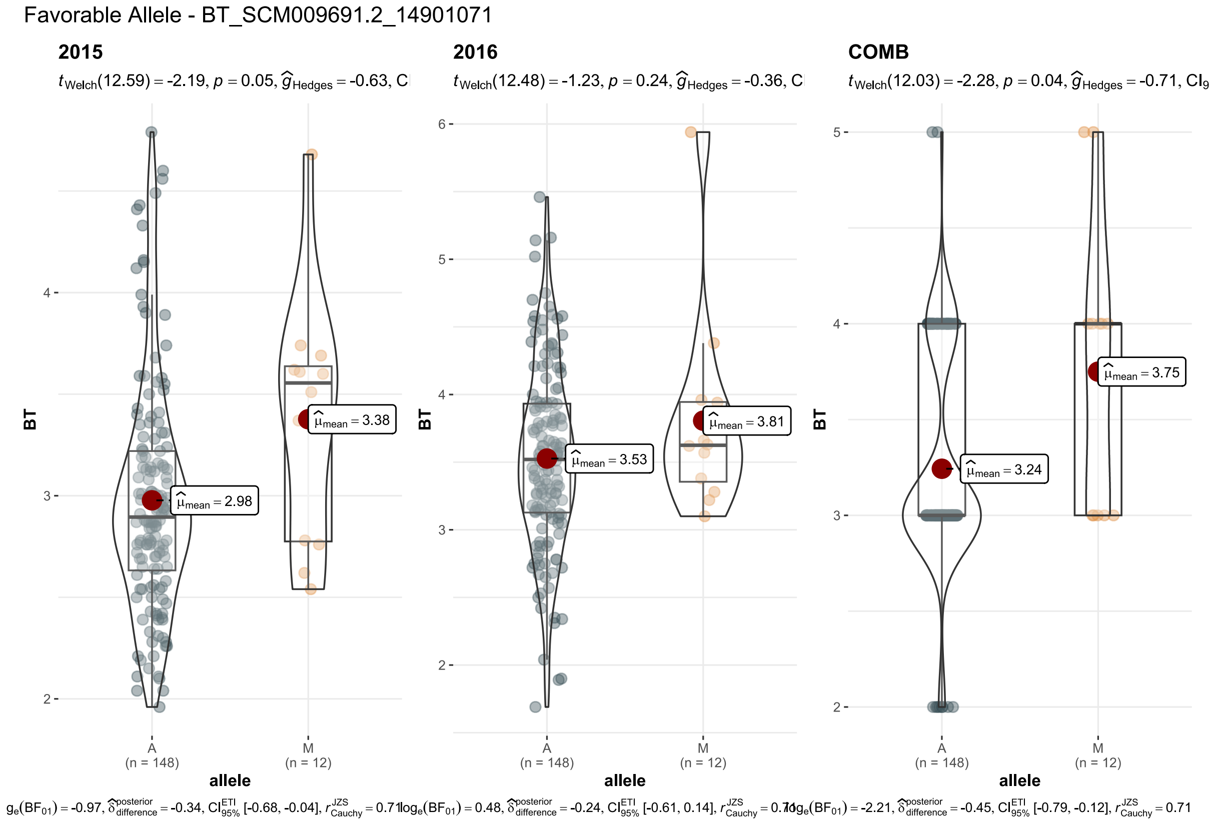

# PLHT = Plant height, cm

Favorable Allele - PLHT_SCM009698.2_39130372

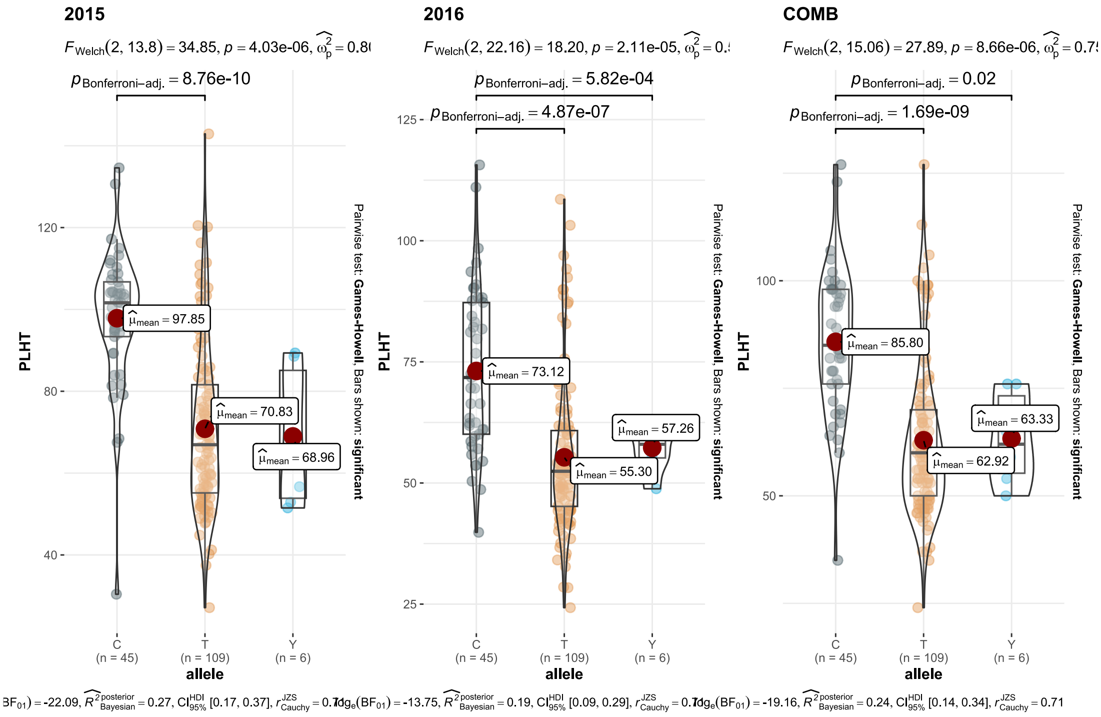


Favorable Allele - PLHT_SCM009696.2_1535098

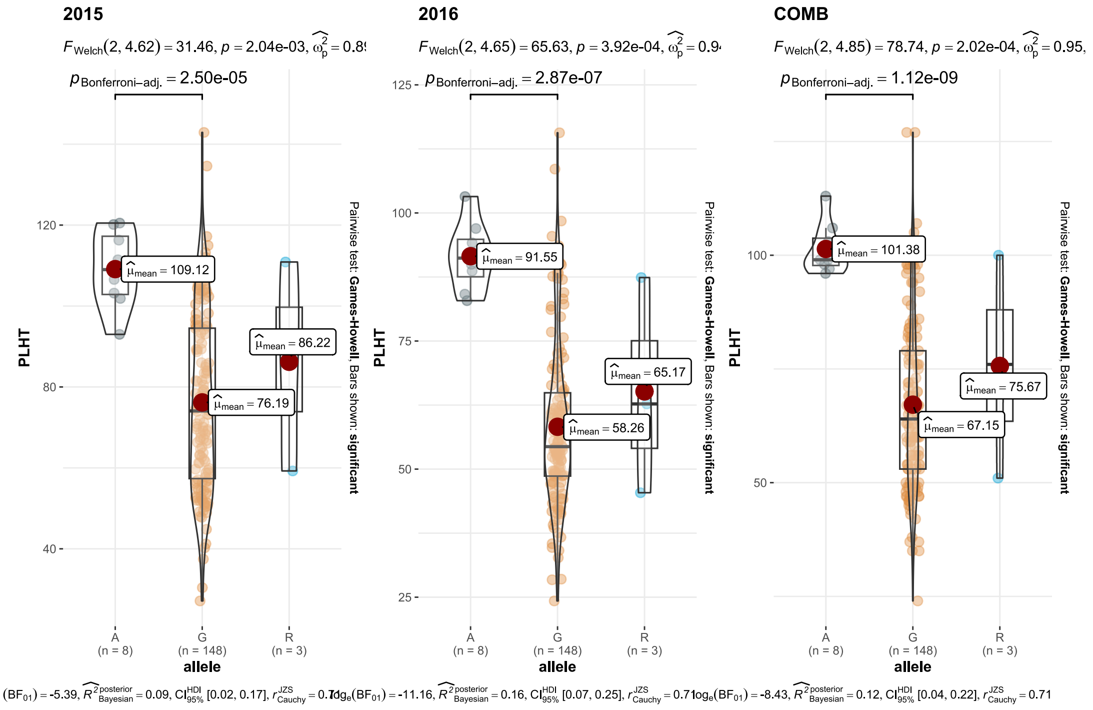

# PEX = Panicle exsertion, mm

Favorable Allele - PEX_SCM009700.2_24126769

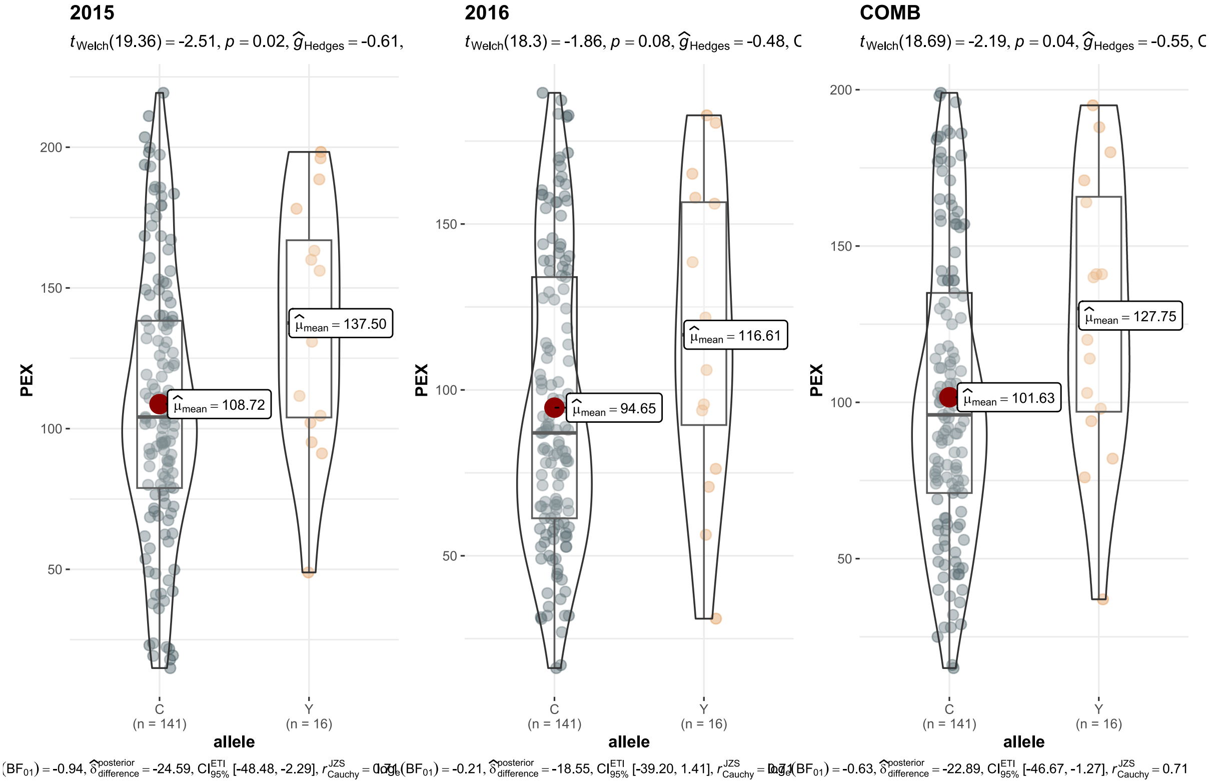

# PEDL = Peduncle length, mm

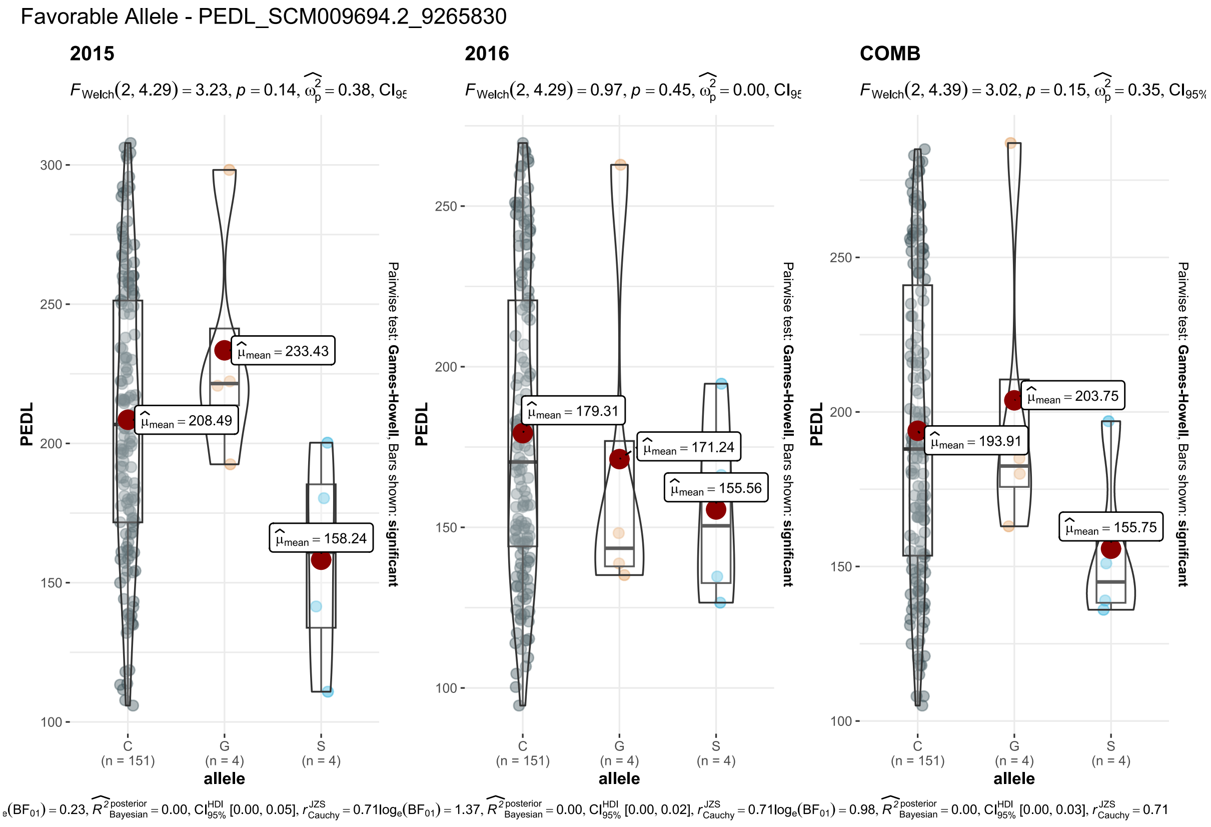

# Nnode=Number of node on main tiller

Favorable Allele - Nnode_SCM009706.2_2540828

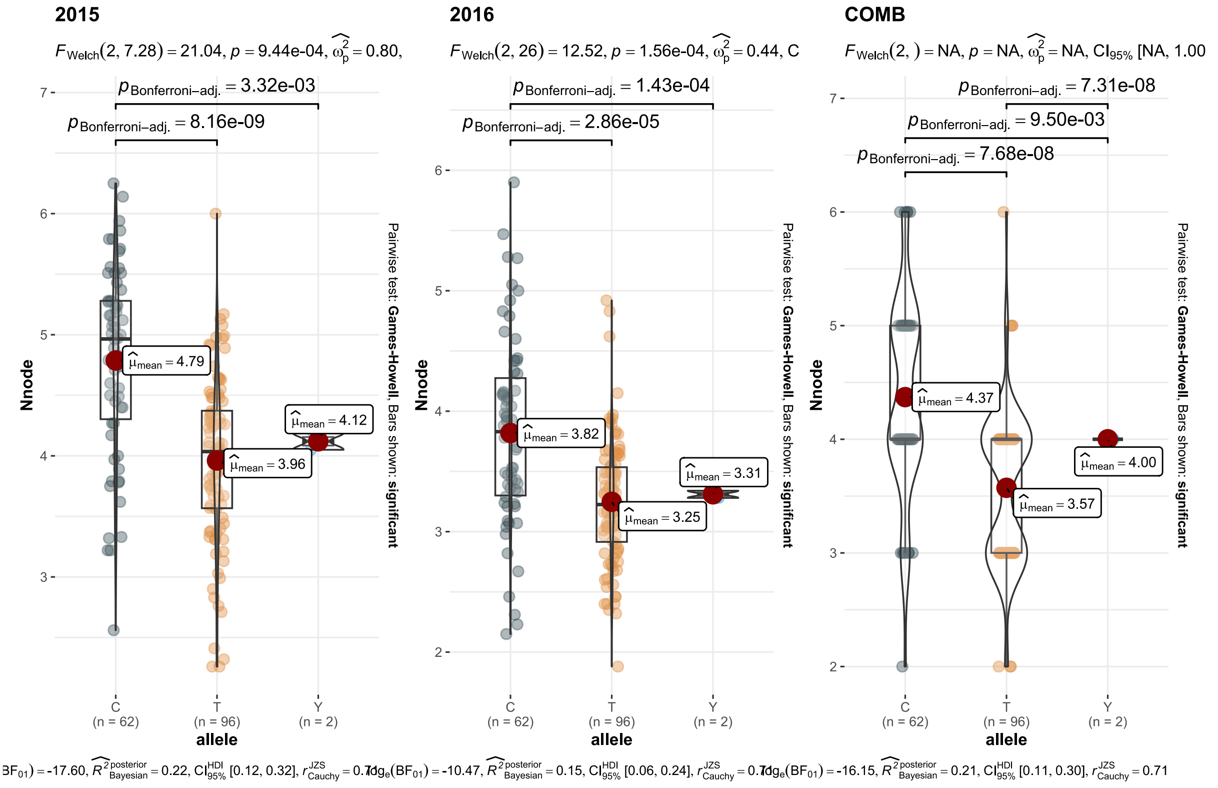


Favorable Allele - Nnode_SCM009705.2_108570

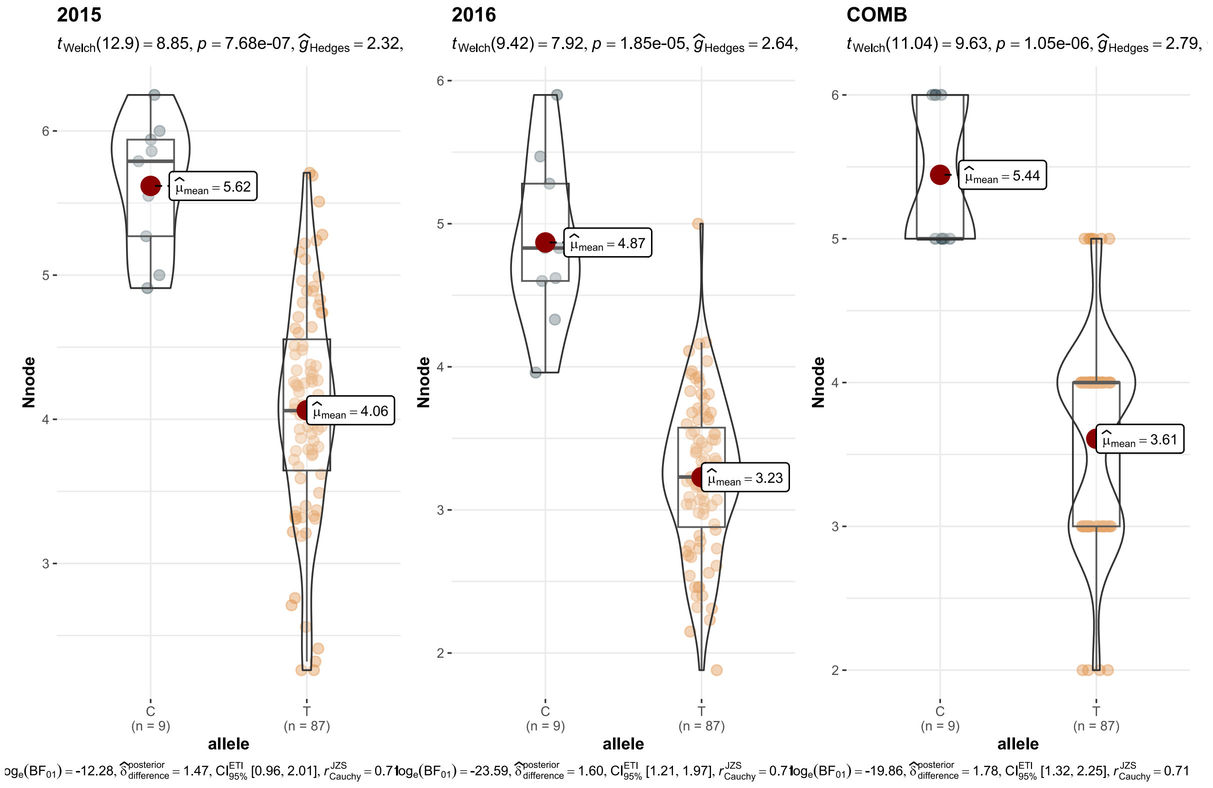

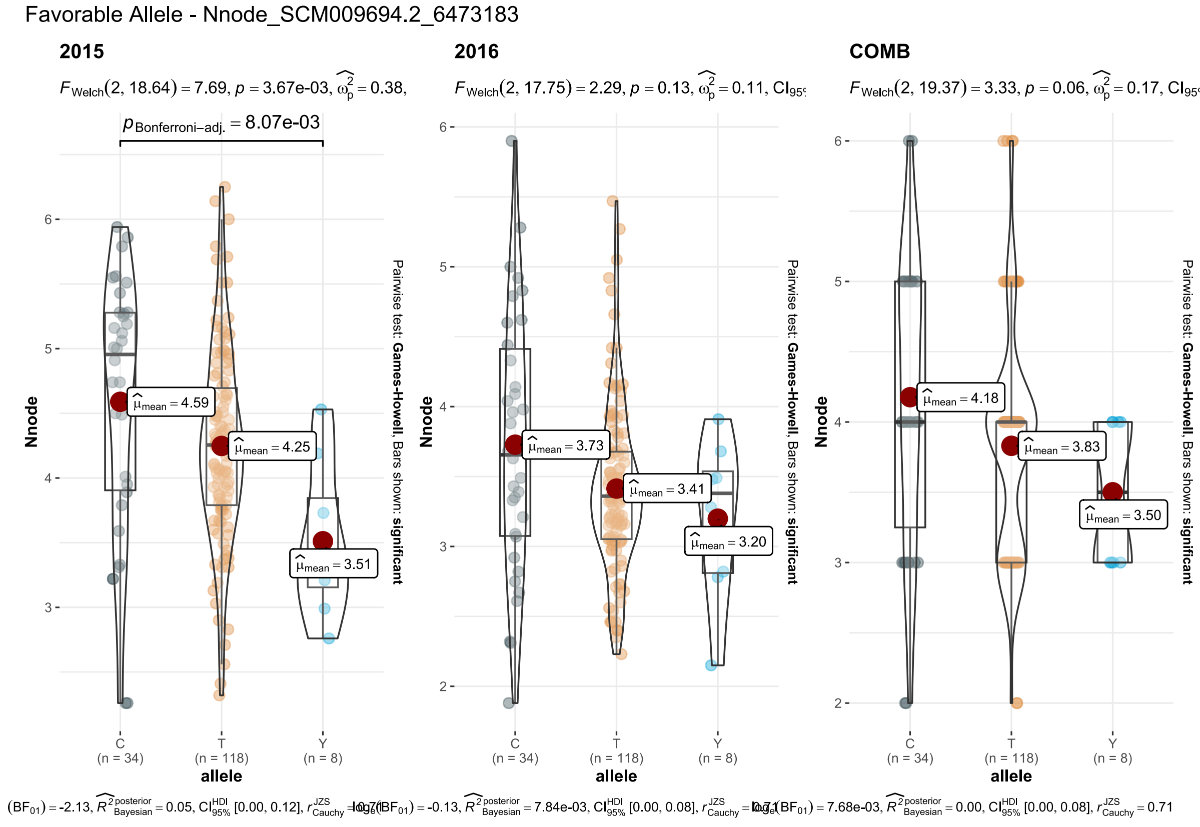


# Maturity = Days to maturity

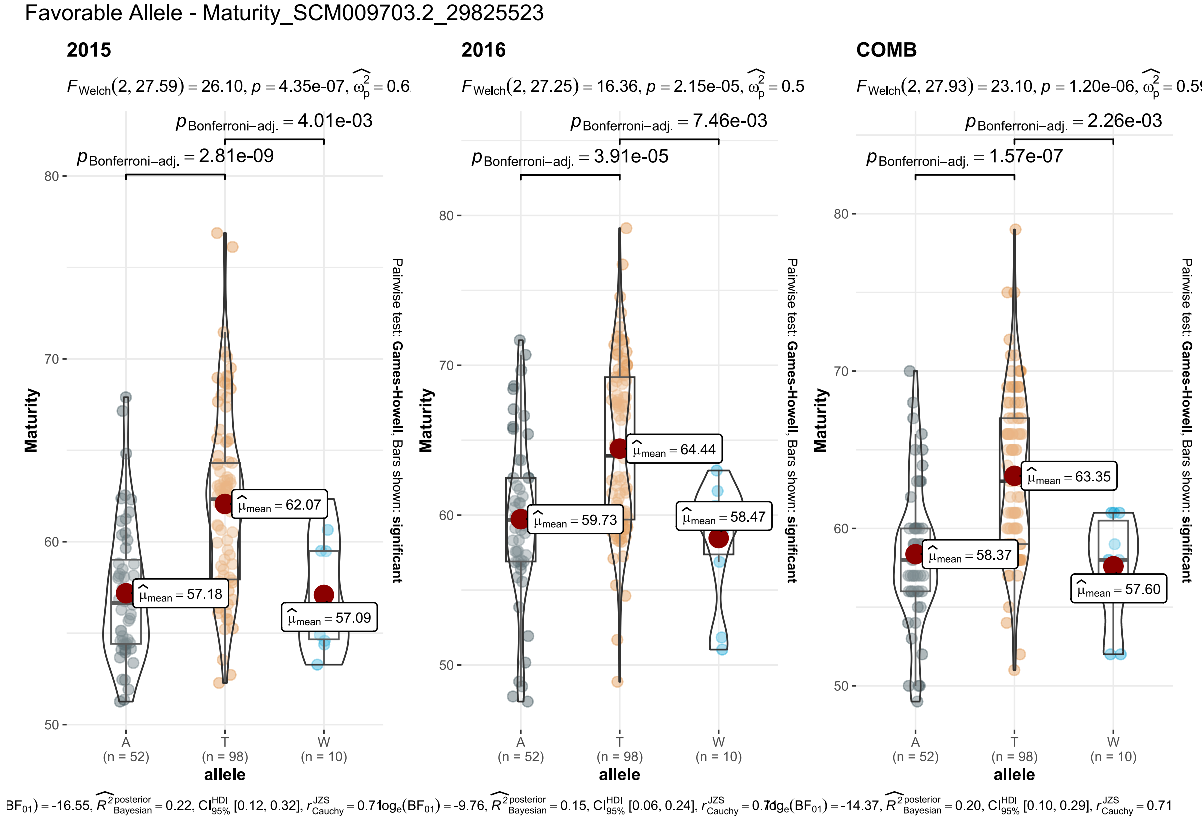

# INFL = Inflorescence length, mm

Favorable Allele - INFL_SCM009703.2_27820106

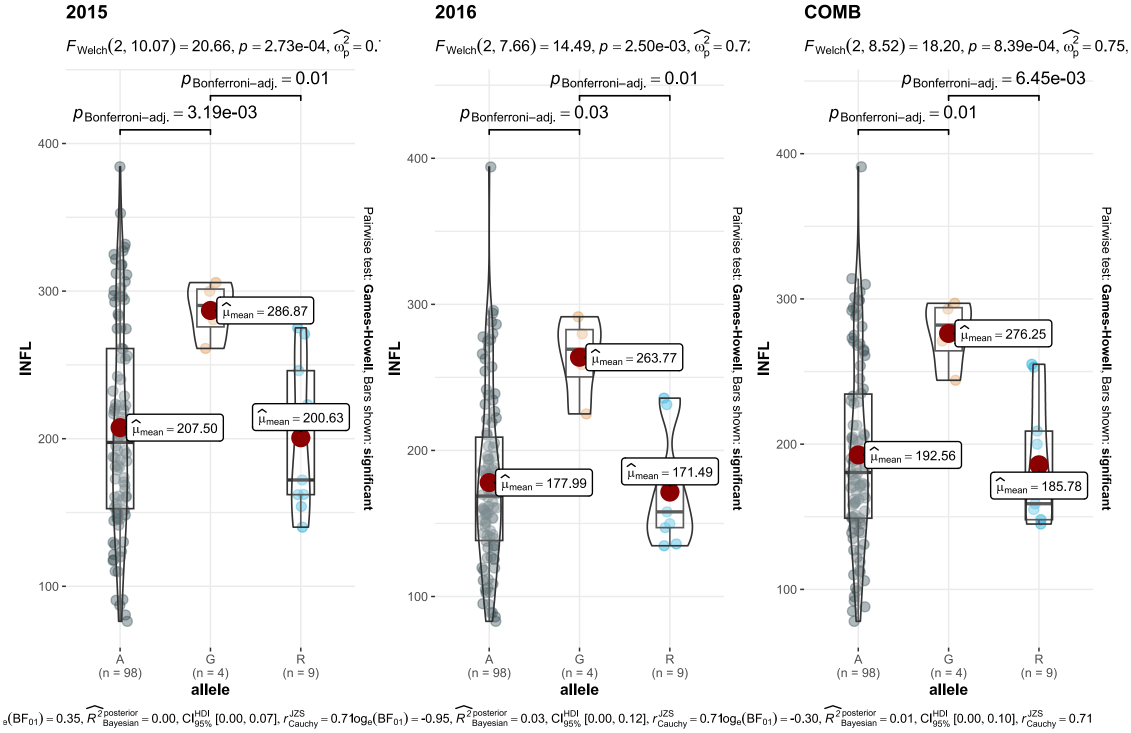


Favorable Allele - INFL_SCM009706.2_3253916

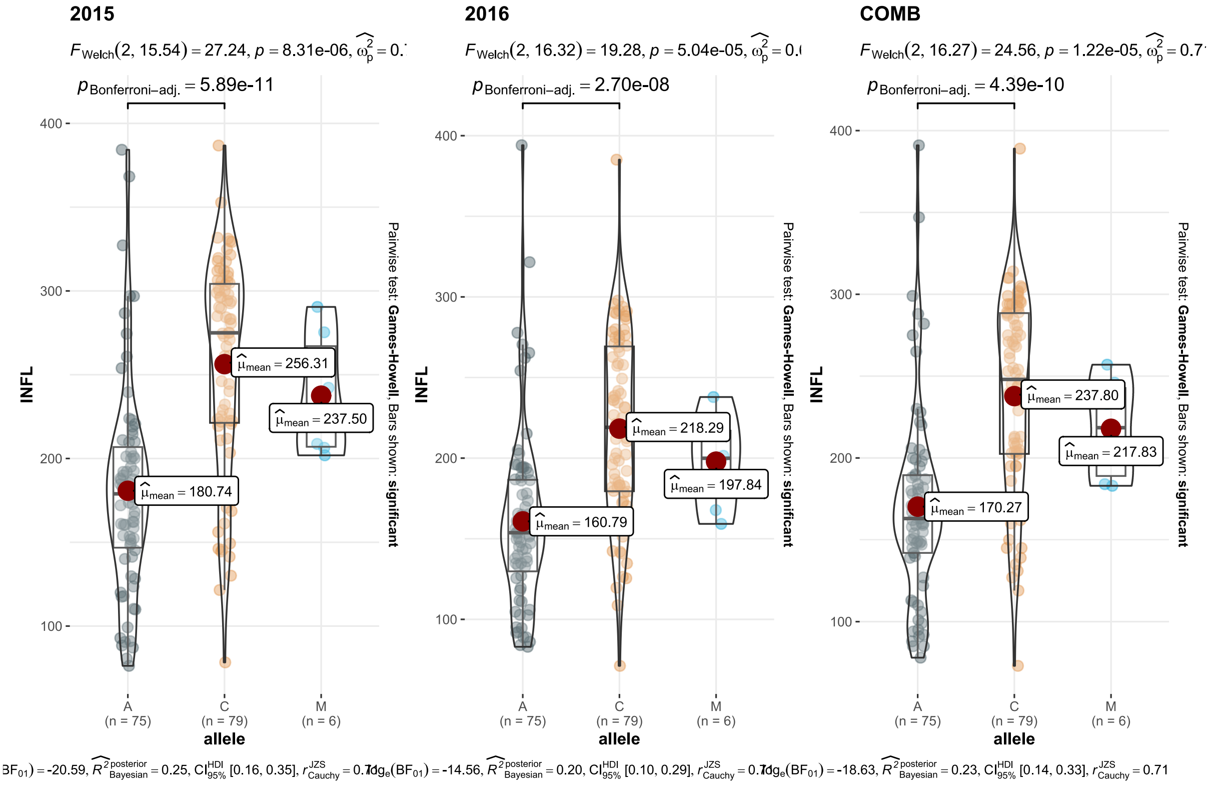

Favorable Allele - INFL_SCM009701.2_34047515

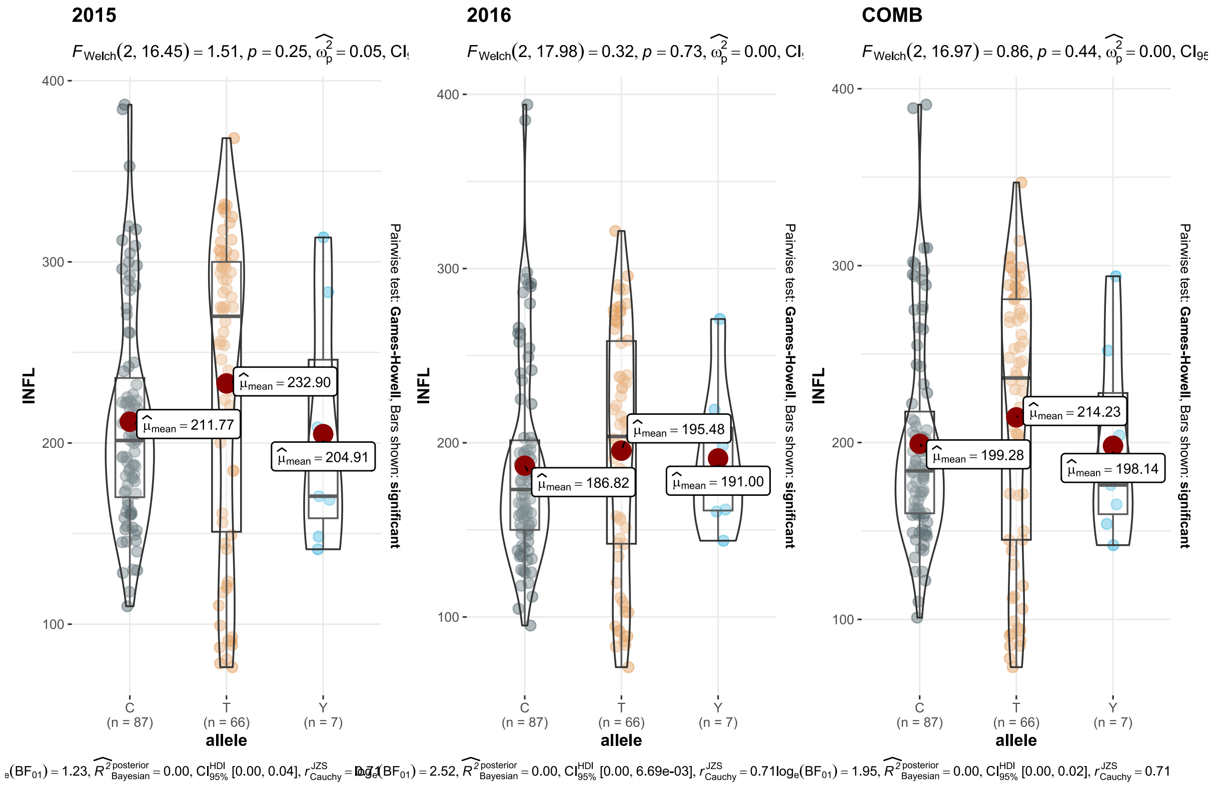


Favorable Allele - INFL_SCM009701.2_41890075

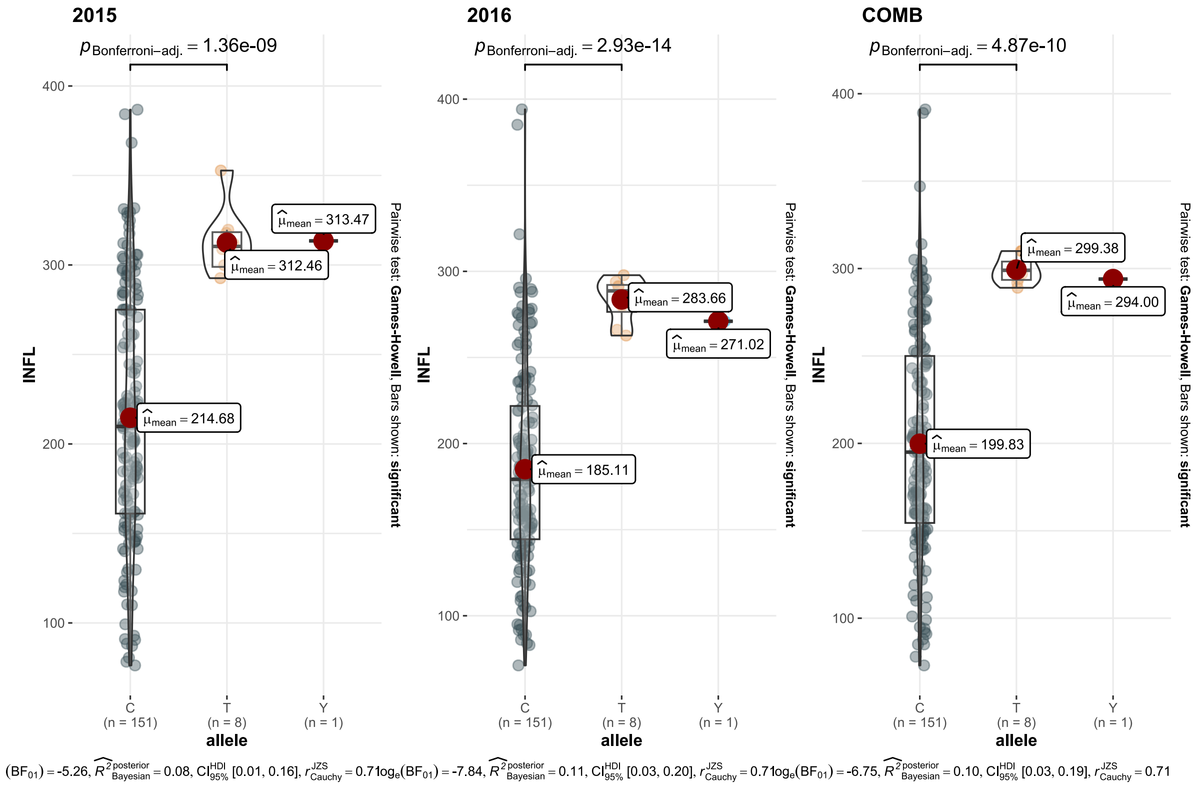

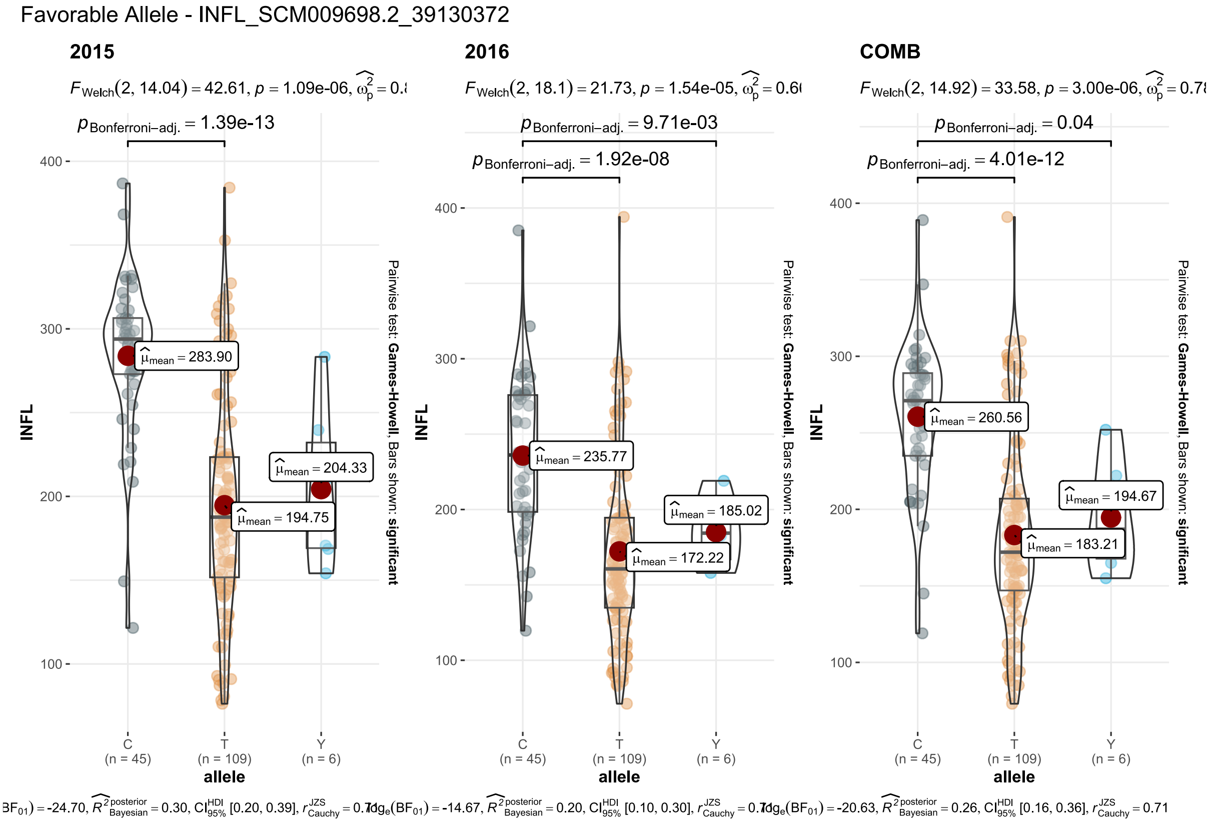
Favorable Allele - INFL_SCM009698.2_39130372
2015
2016
COMB
pBonferroni−adj. = 1.39e-13
pBonferroni−adj. = 9.71e-03
pBonferroni−adj. = 1.92e-08
pBonferroni−adj. = 0.04
pBonferroni−adj. = 4.01e-12
μmean = 283.90
μmean = 204.33
μmean = 194.75
μmean = 235.77
μmean = 185.02
μmean = 172.22
μmean = 260.56
μmean = 194.67
μmean = 183.21
INFL
allele
C (n = 45)
T (n = 109)
Y (n = 6)
Pairwise test: Games-Howell. Bars shown: significant

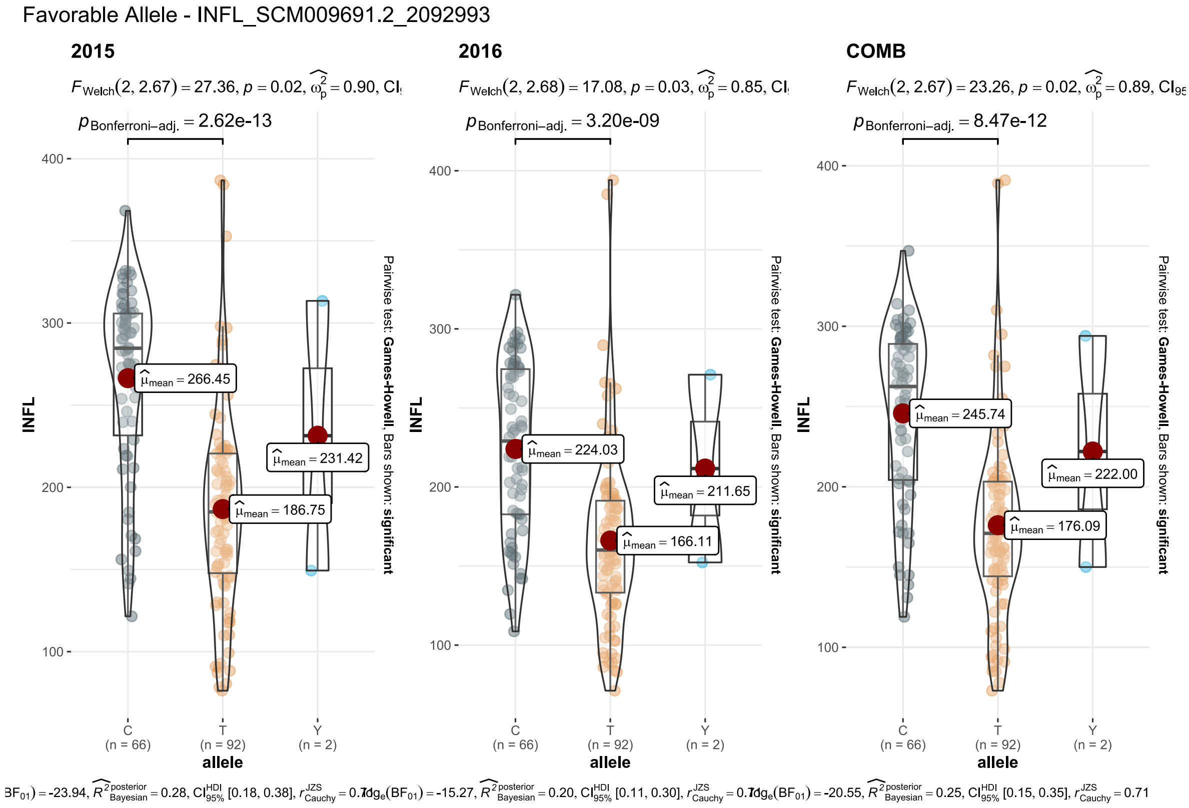
Favorable Allele - INFL_SCM009691.2_2092993
2015
2016
COMB
pBonferroni−adj. = 2.62e-13
pBonferroni−adj. = 3.20e-09
pBonferroni−adj. = 8.47e-12
μmean = 266.45
μmean = 231.42
μmean = 186.75
μmean = 224.03
μmean = 211.65
μmean = 166.11
μmean = 245.74
μmean = 222.00
μmean = 176.09
INFL
allele
C (n = 66)
T (n = 92)
Y (n = 2)
Pairwise test: Games-Howell. Bars shown: significant

Favorable Allele - INFL_SCM009690.2_8346815

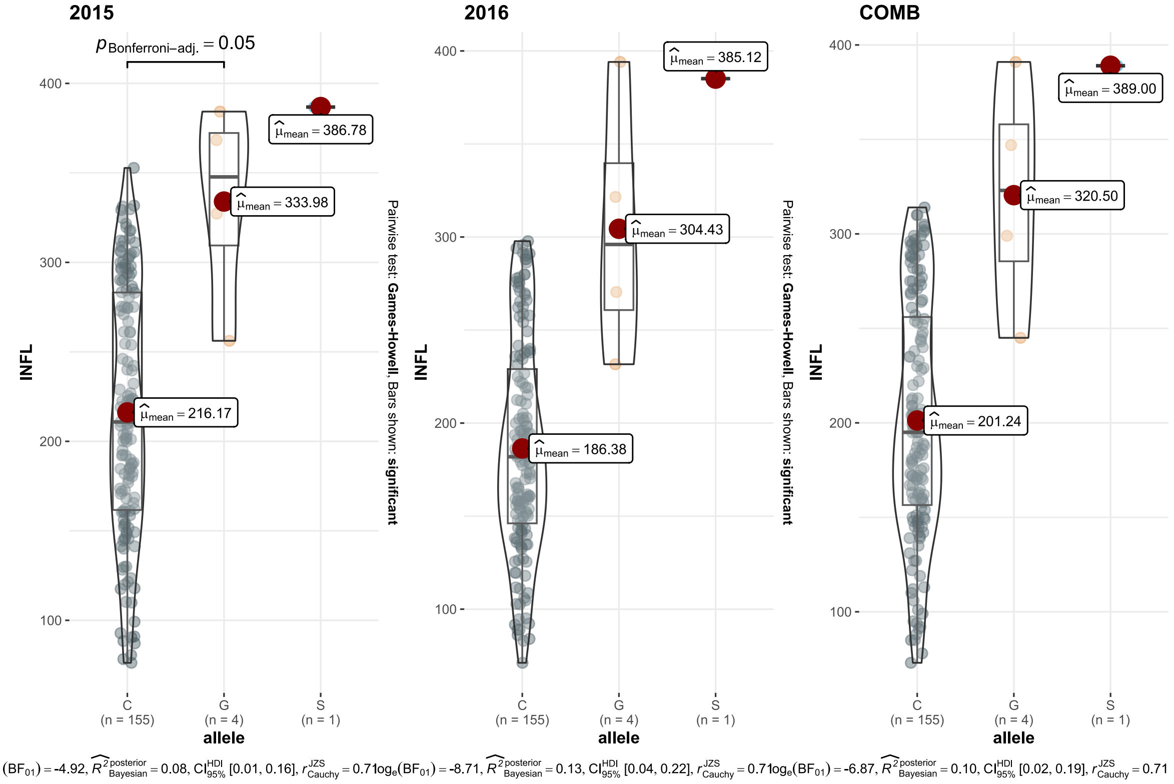

# INF_PBN = Inflorescence primary branch number

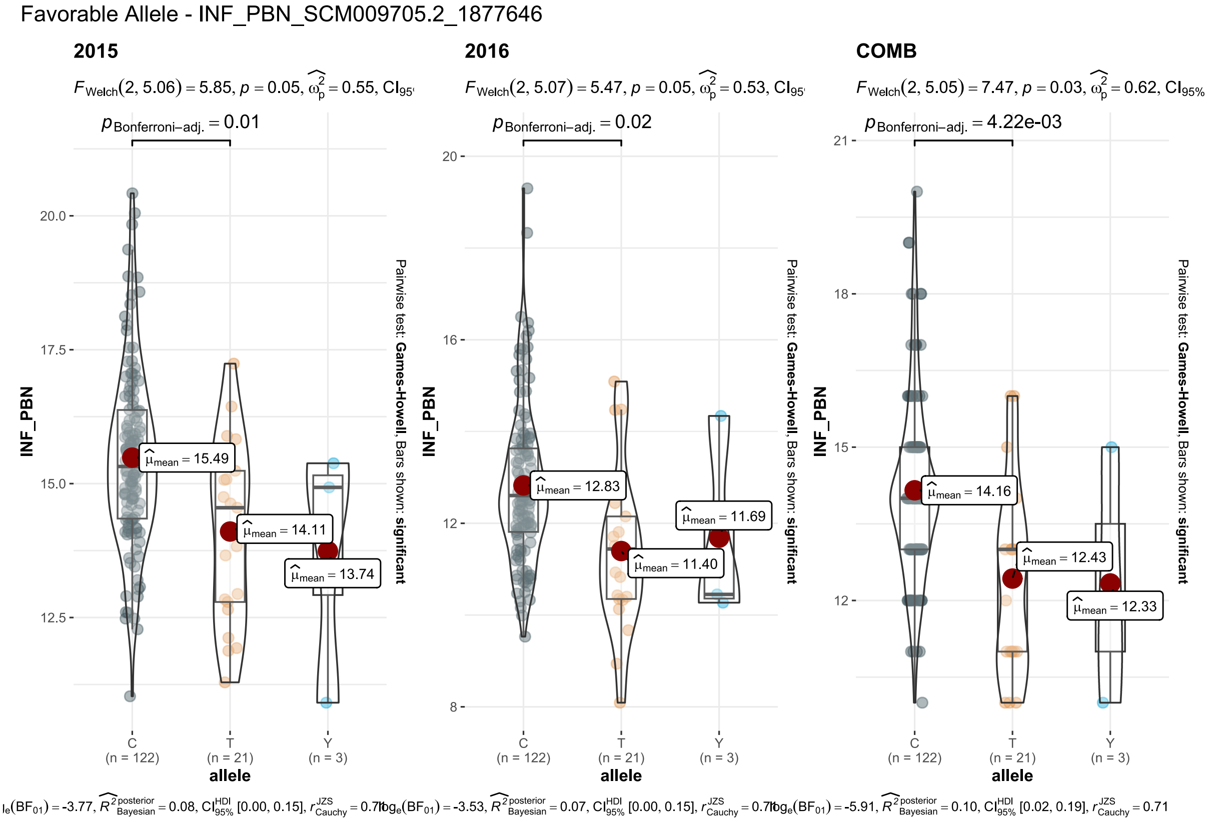


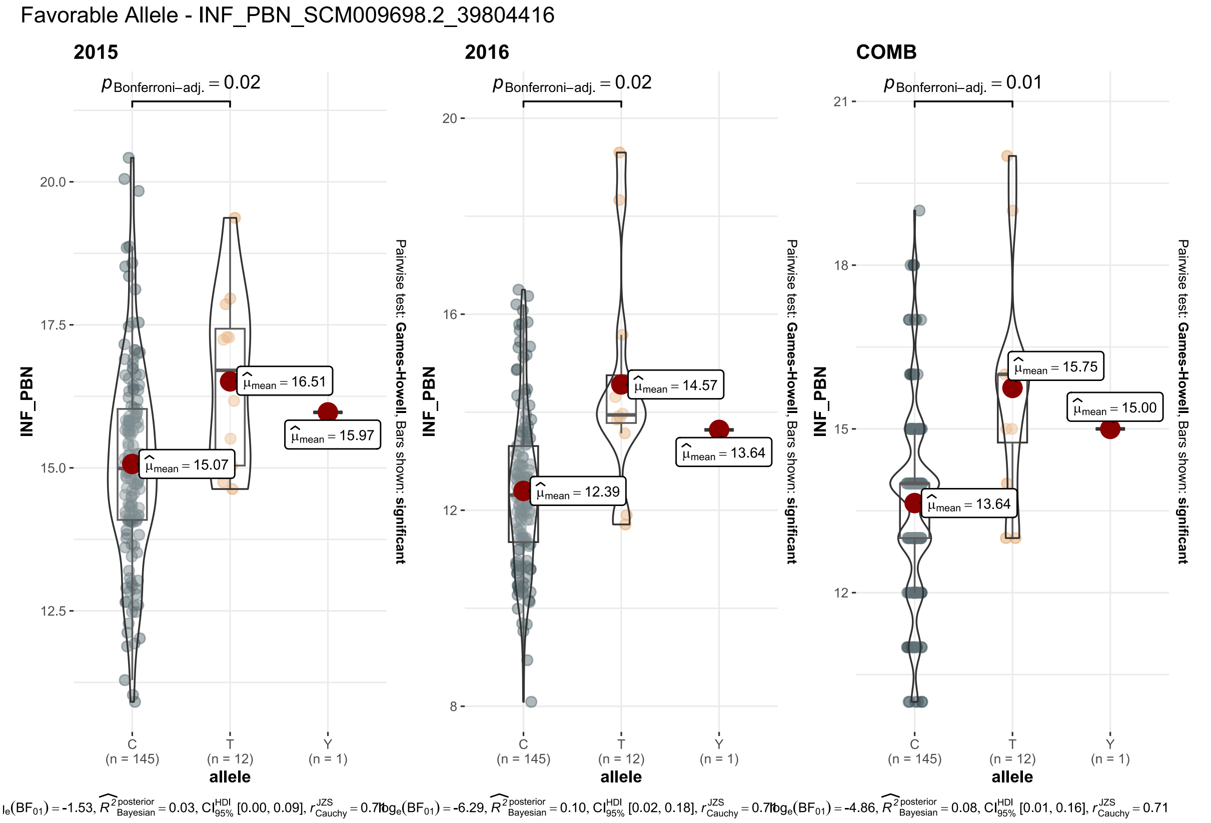

## Favorable Allele - INF_PBN_SCM009696.2_1535098

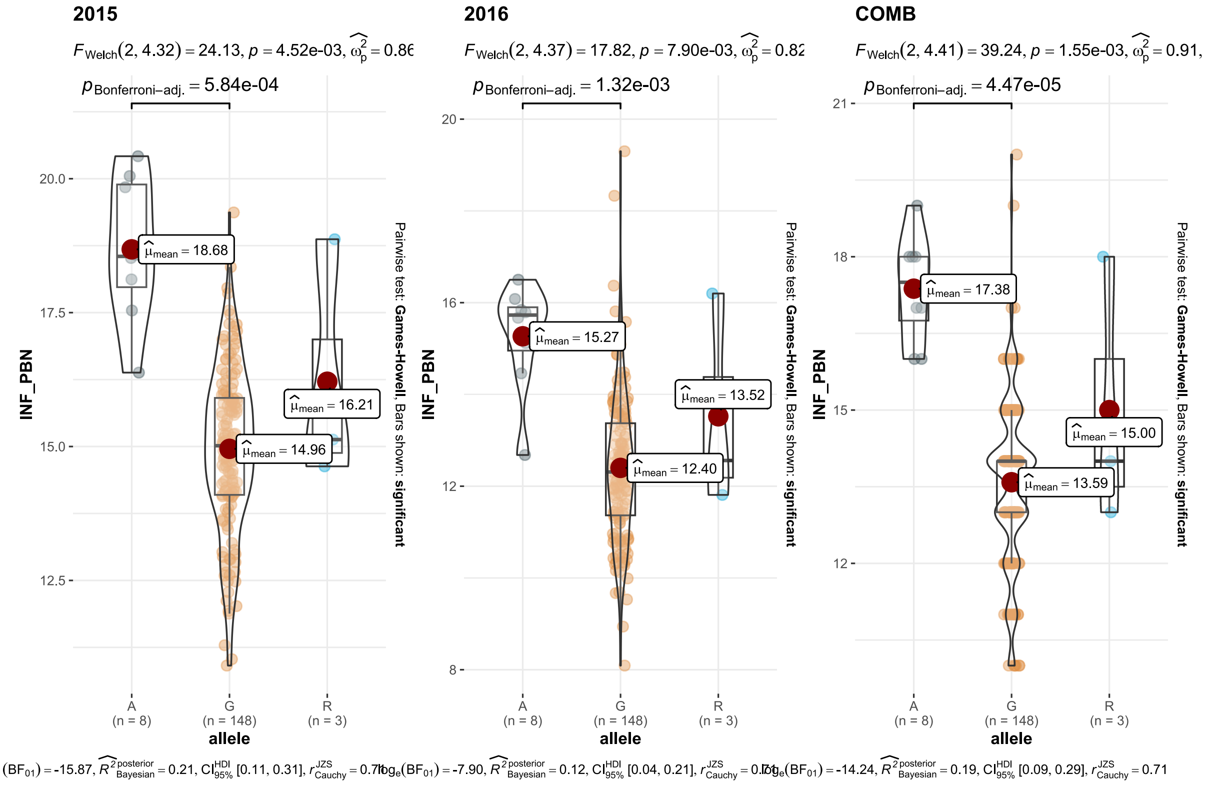


## Favorable Allele - INF_PBN_SCM009694.2_15388933

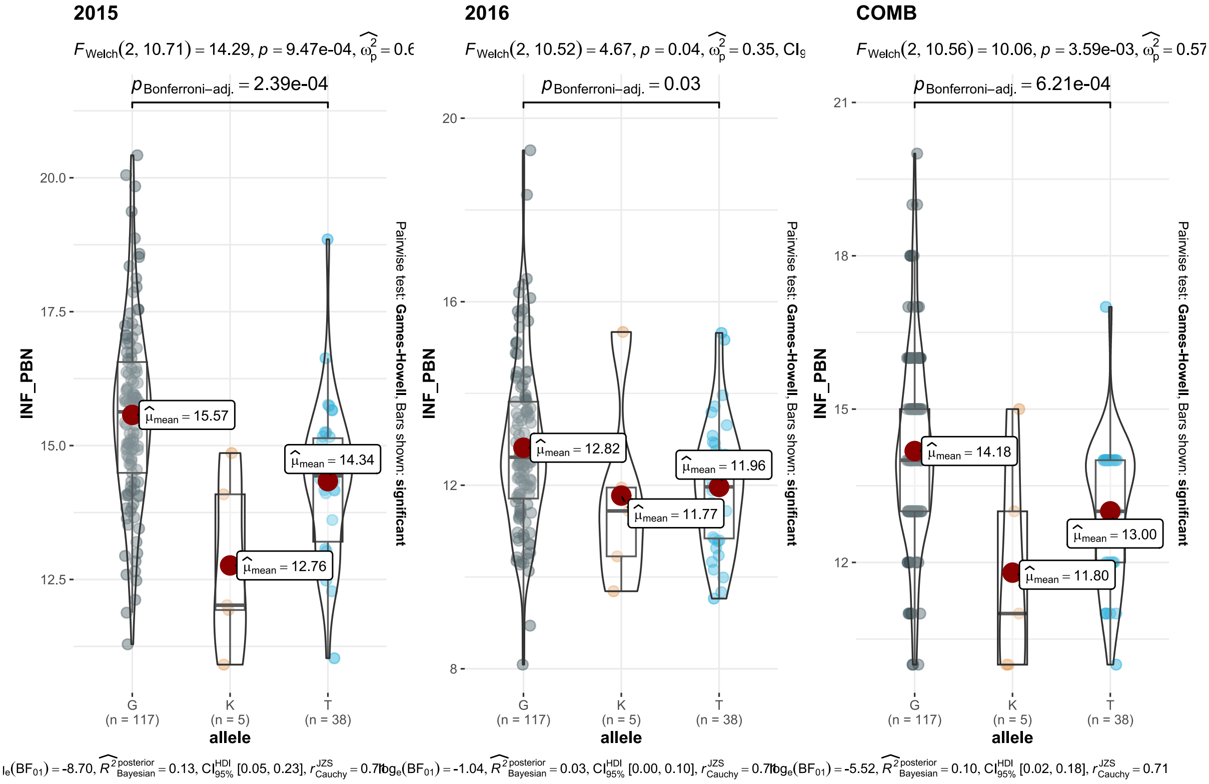

# HSW = 100 seed weight, g

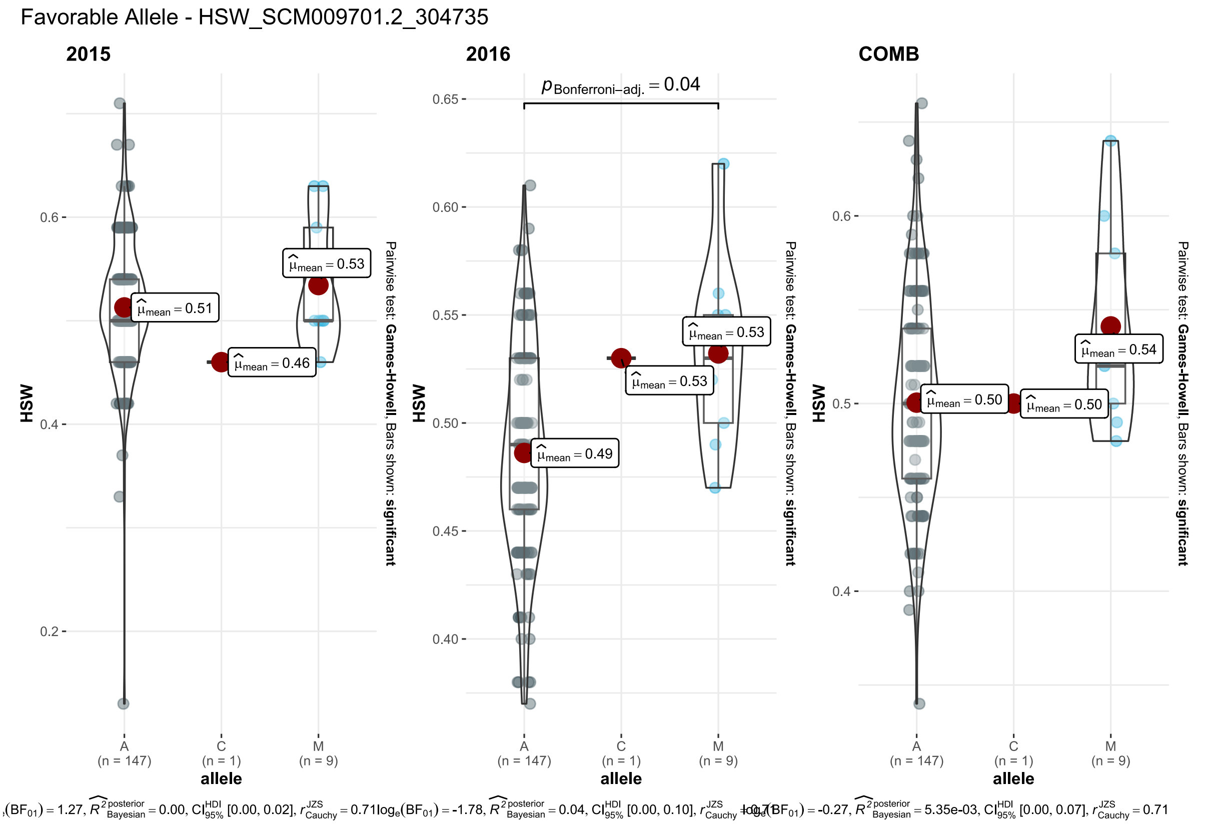


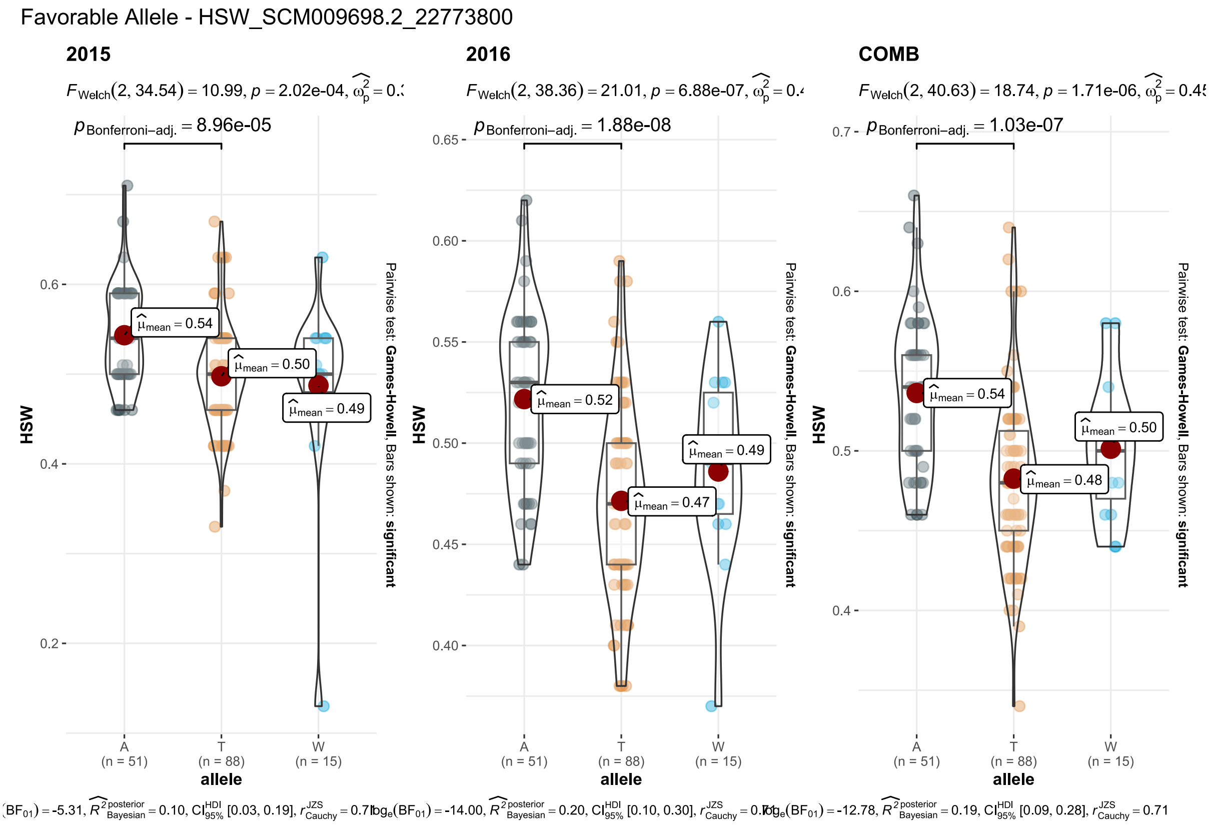

# FLSL = Flag leaf sheath length, mm

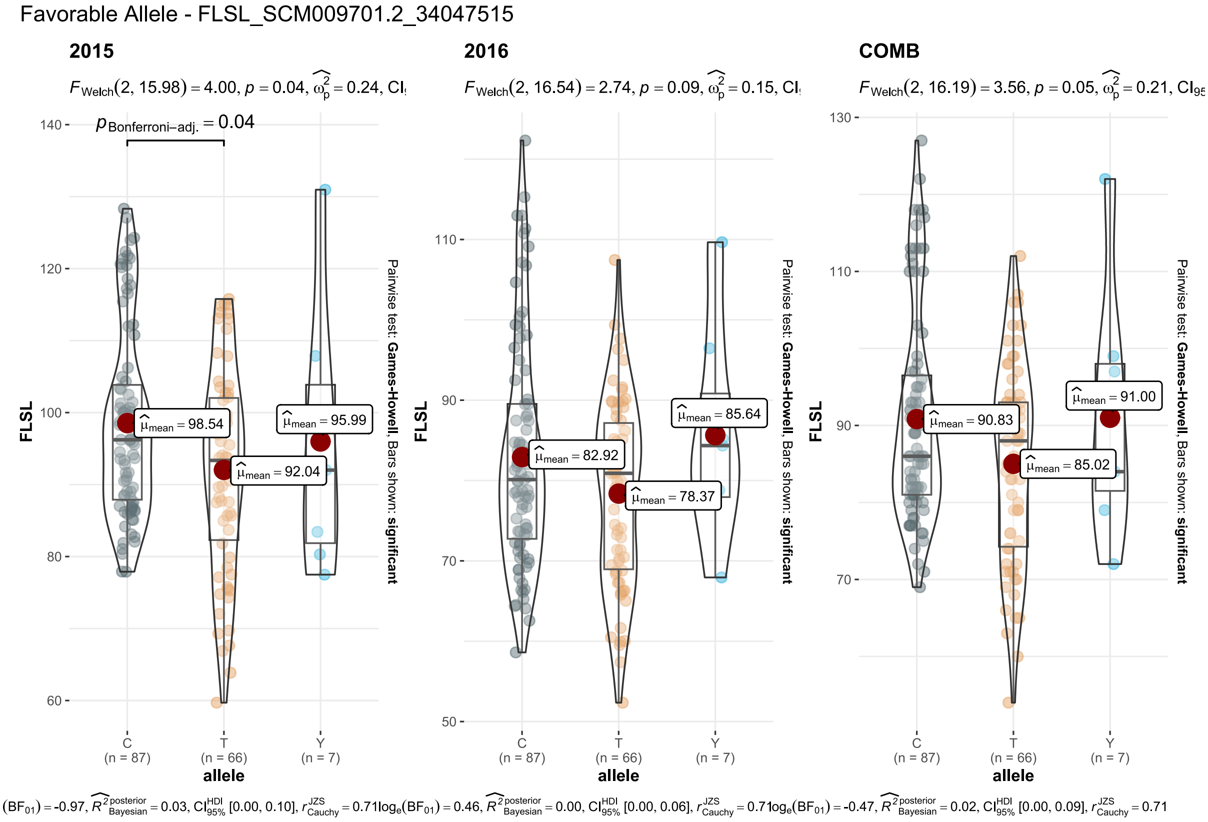


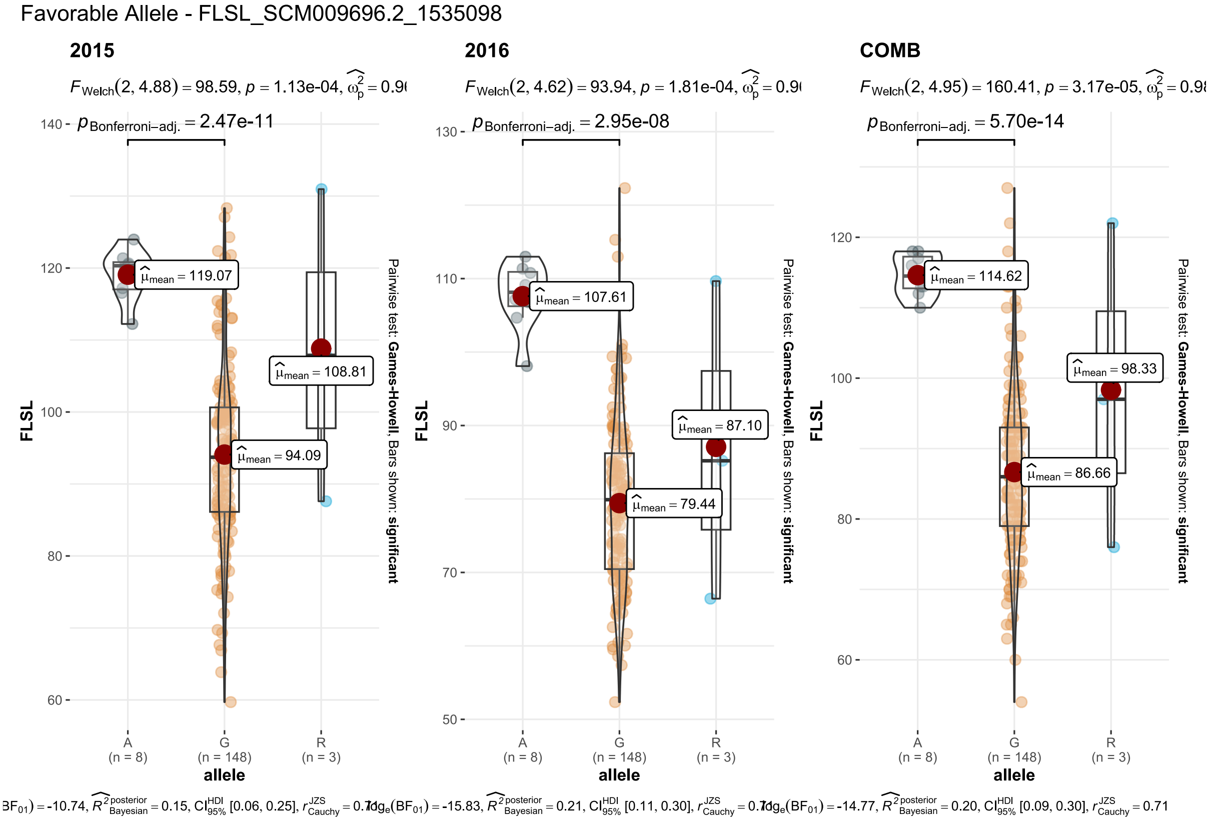

# FLSL = Flag leaf blade length, mm

Favorable Allele - FLBL_SCM009707.2_4062363

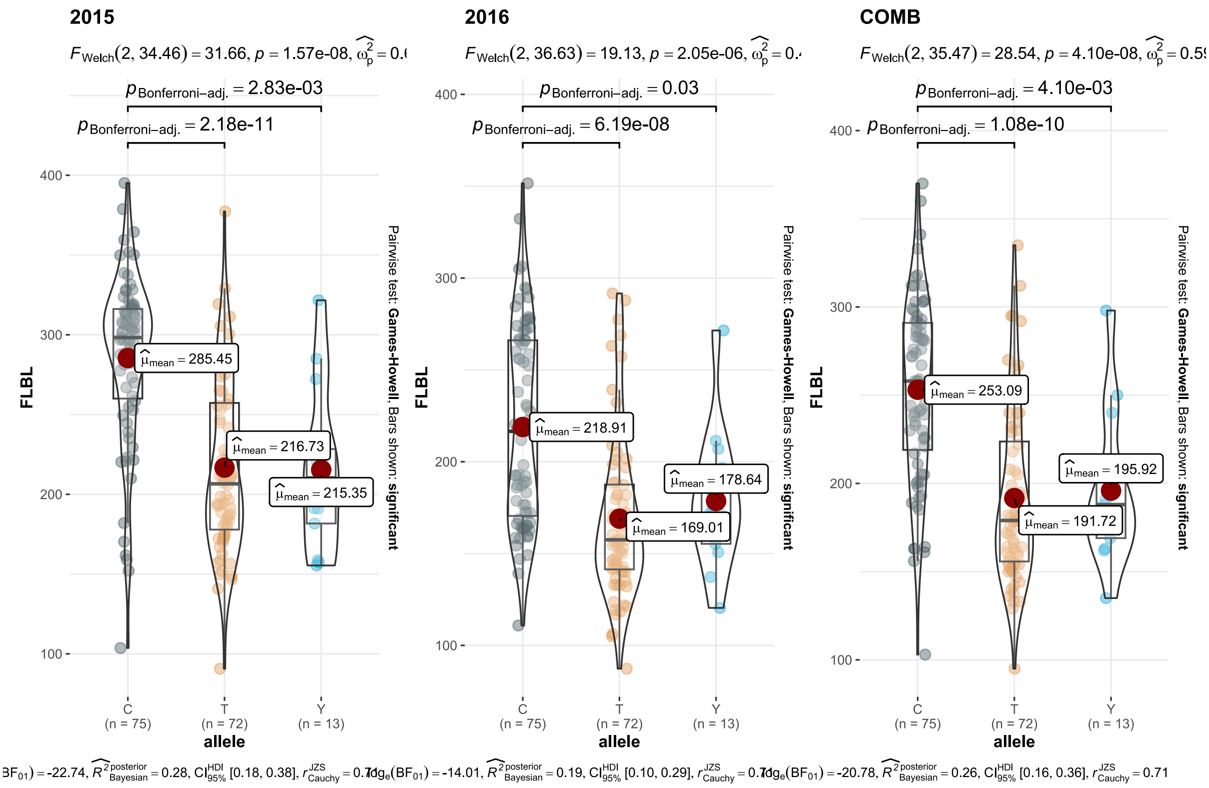


Favorable Allele - FLBL_SCM009706.2_2816771

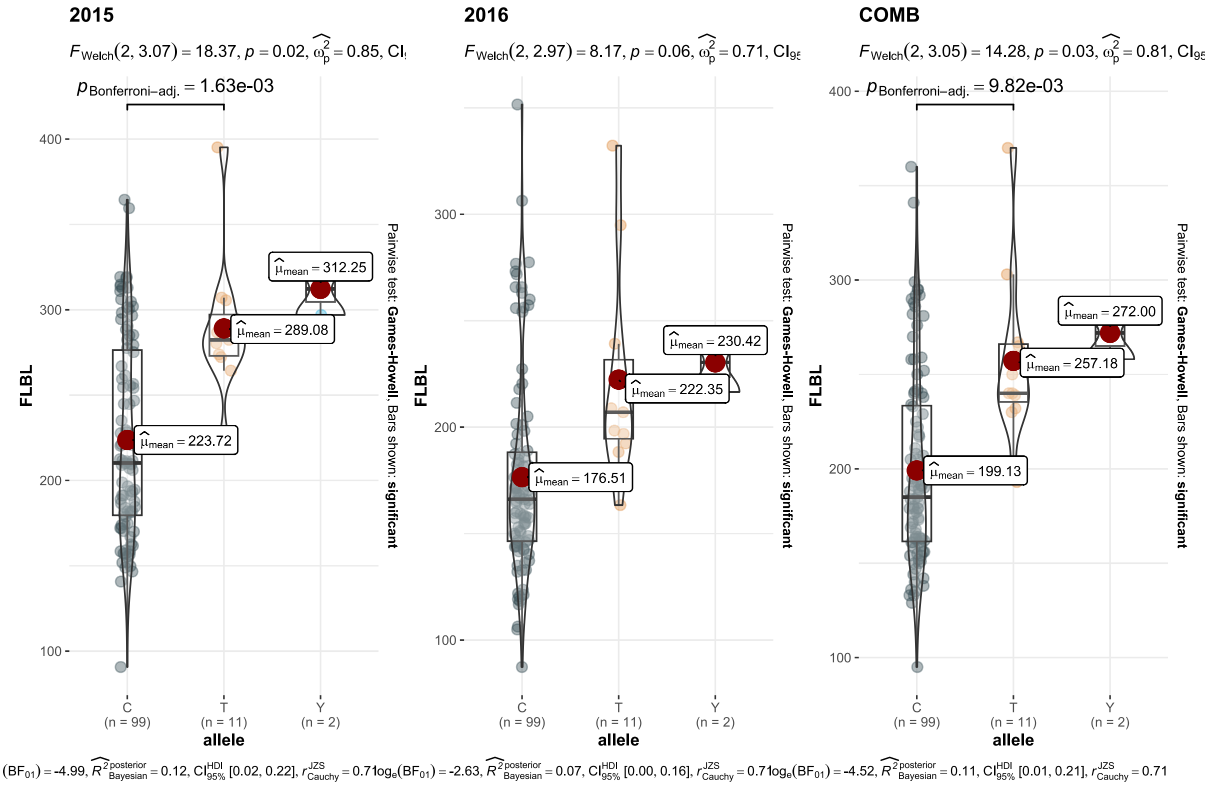

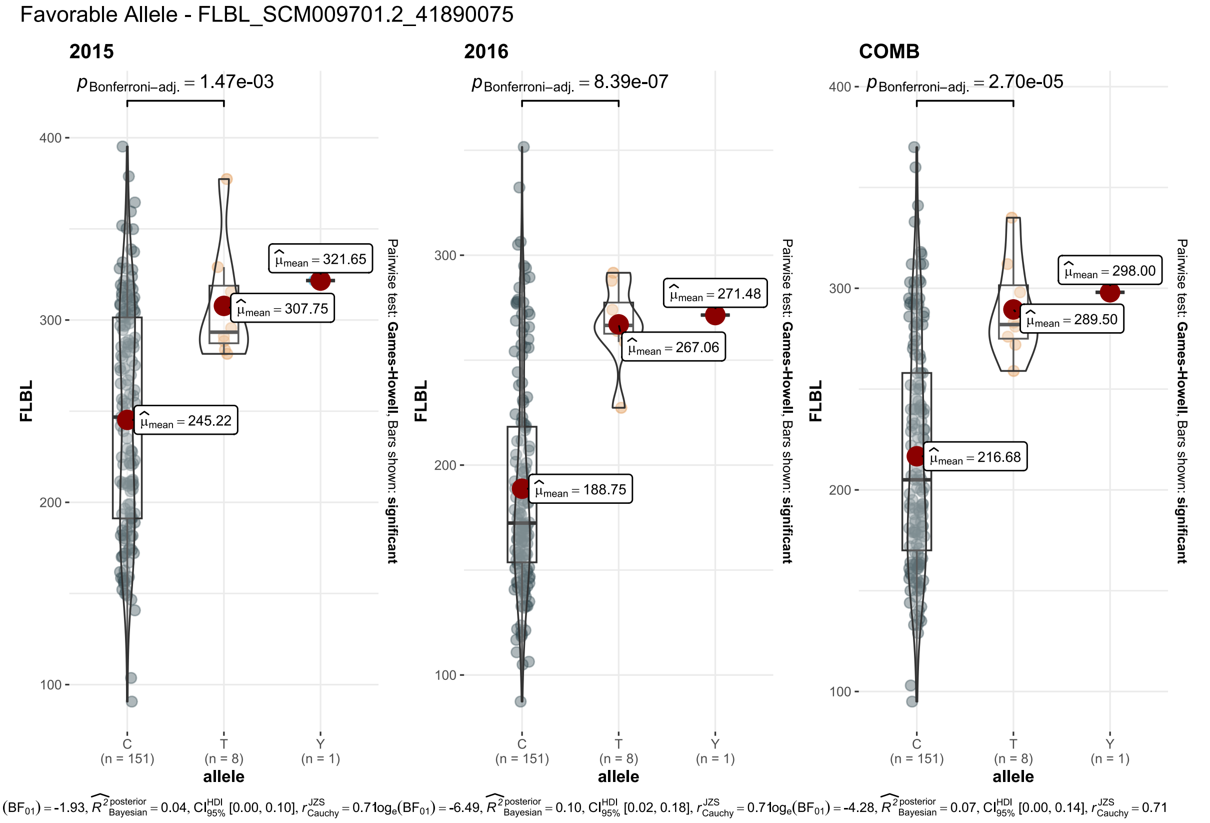
Favorable Allele - FLBL_SCM009701.2_41890075
2015
2016
COMB
pBonferroni−adj. = 1.47e-03
pBonferroni−adj. = 8.39e-07
pBonferroni−adj. = 2.70e-05
μmean = 321.65
μmean = 307.75
μmean = 245.22
μmean = 271.48
μmean = 267.06
μmean = 188.75
μmean = 298.00
μmean = 289.50
μmean = 216.68
FLBL
Pairwise test: Games-Howell, Bars shown: significant
C
T
Y
(n = 151)
(n = 8)
(n = 1)
allele

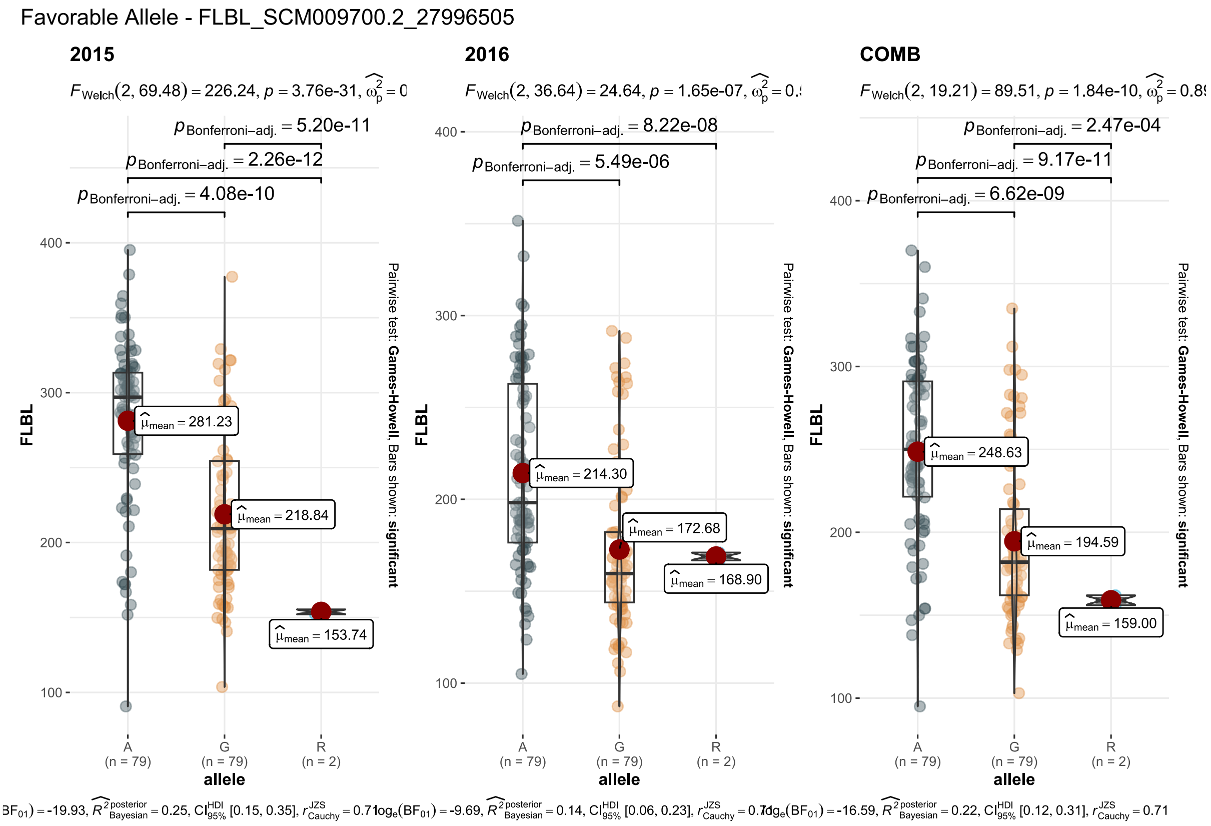
Favorable Allele - FLBL_SCM009700.2_27996505
2015
2016
COMB
FWelch(2, 69.48) = 226.24, p = 3.76e-31
FWelch(2, 36.64) = 24.64, p = 1.65e-07
FWelch(2, 19.21) = 89.51, p = 1.84e-10
pBonferroni−adj. = 5.20e-11
pBonferroni−adj. = 2.26e-12
pBonferroni−adj. = 4.08e-10
pBonferroni−adj. = 8.22e-08
pBonferroni−adj. = 5.49e-06
pBonferroni−adj. = 2.47e-04
pBonferroni−adj. = 9.17e-11
pBonferroni−adj. = 6.62e-09
μmean = 281.23
μmean = 218.84
μmean = 153.74
μmean = 214.30
μmean = 172.68
μmean = 168.90
μmean = 248.63
μmean = 194.59
μmean = 159.00
FLBL
Pairwise test: Games-Howell, Bars shown: significant
A
G
R
(n = 79)
(n = 79)
(n = 2)
allele

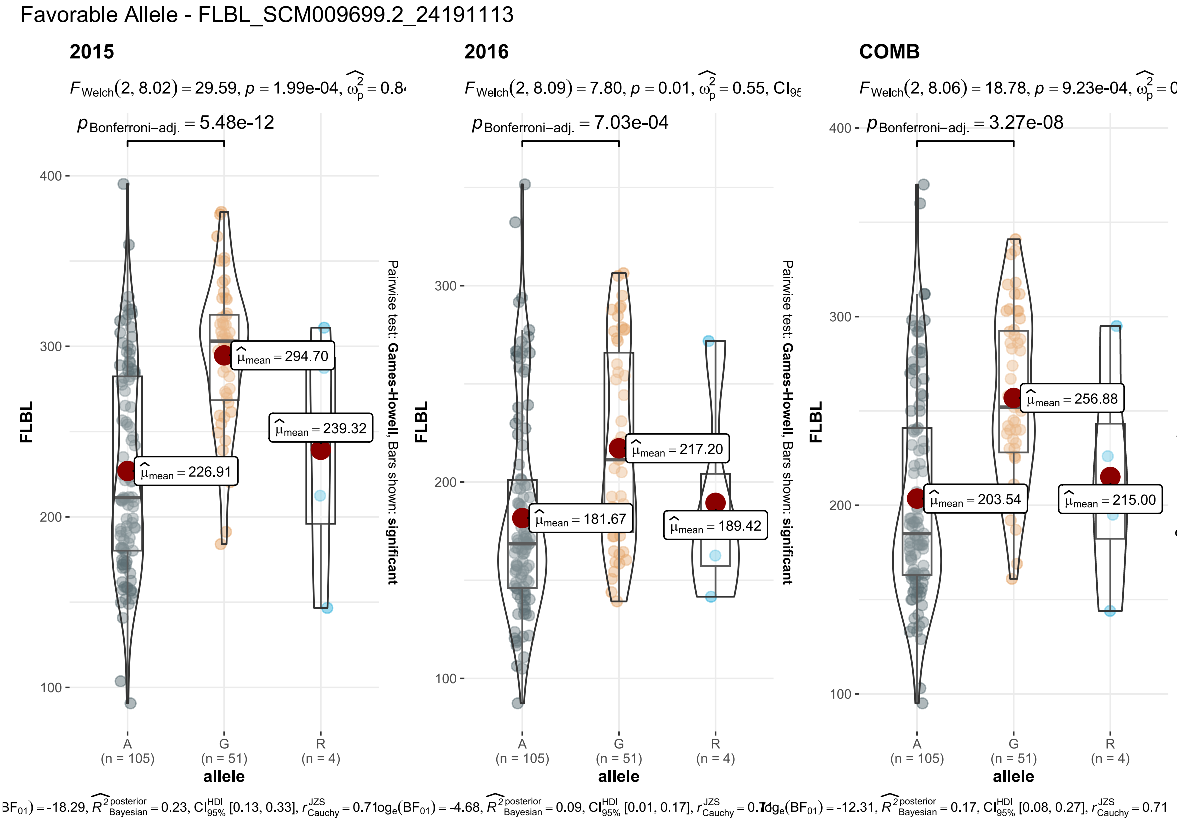
Favorable Allele - FLBL_SCM009699.2_24191113
2015
2016
COMB
$F_{Welch}(2, 8.02) = 29.59$, $p = 1.99e-04$
$F_{Welch}(2, 8.09) = 7.80$, $p = 0.01$, $\widehat{\omega_p^2} = 0.55$
$F_{Welch}(2, 8.06) = 18.78$, $p = 9.23e-04$, $\widehat{\omega_p^2} = 0.76$
$p_{Bonferroni-adj.} = 5.48e-12$
$p_{Bonferroni-adj.} = 7.03e-04$
$p_{Bonferroni-adj.} = 3.27e-08$
$\hat{\mu}_{mean} = 226.91$
$\hat{\mu}_{mean} = 294.70$
$\hat{\mu}_{mean} = 239.32$
$\hat{\mu}_{mean} = 181.67$
$\hat{\mu}_{mean} = 217.20$
$\hat{\mu}_{mean} = 189.42$
$\hat{\mu}_{mean} = 203.54$
$\hat{\mu}_{mean} = 256.88$
$\hat{\mu}_{mean} = 215.00$
FLBL
allele
A (n = 105)
G (n = 51)
R (n = 4)
Pairwise test: Games-Howell, Bars shown: significant
$\log_e(BF_{01}) = -4.68$, $\widehat{R^2}^{posterior}_{Bayesian} = 0.09$, $CI^{HDI}_{95\%}$ [0.01, 0.17], $r^{JZS}_{Cauchy} = 0.71$
$\log_e(BF_{01}) = -12.31$, $\widehat{R^2}^{posterior}_{Bayesian} = 0.17$, $CI^{HDI}_{95\%}$ [0.08, 0.27], $r^{JZS}_{Cauchy} = 0.71$

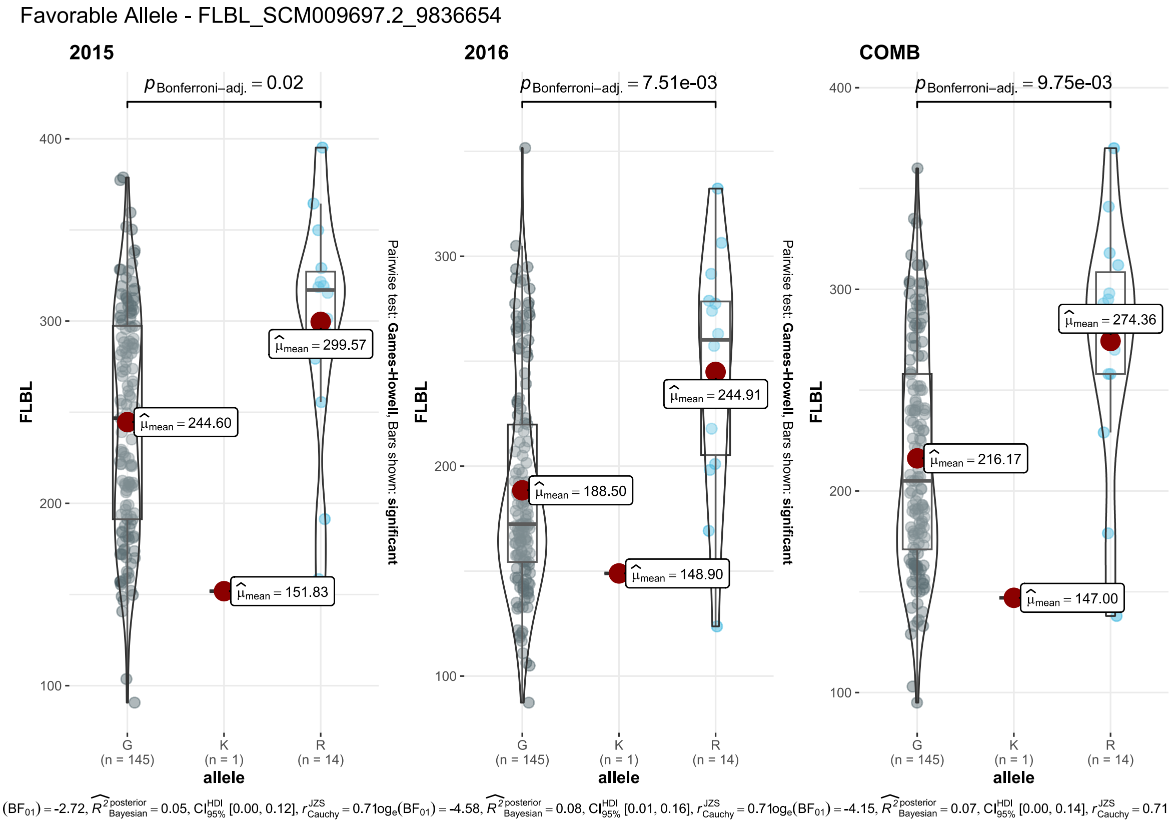
Favorable Allele - FLBL_SCM009697.2_9836654
2015
2016
COMB
$p_{Bonferroni-adj.} = 0.02$
$p_{Bonferroni-adj.} = 7.51e-03$
$p_{Bonferroni-adj.} = 9.75e-03$
$\hat{\mu}_{mean} = 244.60$
$\hat{\mu}_{mean} = 151.83$
$\hat{\mu}_{mean} = 299.57$
$\hat{\mu}_{mean} = 188.50$
$\hat{\mu}_{mean} = 148.90$
$\hat{\mu}_{mean} = 244.91$
$\hat{\mu}_{mean} = 216.17$
$\hat{\mu}_{mean} = 147.00$
$\hat{\mu}_{mean} = 274.36$
FLBL
allele
G (n = 145)
K (n = 1)
R (n = 14)
Pairwise test: Games-Howell, Bars shown: significant
$(BF_{01}) = -2.72$, $\widehat{R^2}^{posterior}_{Bayesian} = 0.05$, $CI^{HDI}_{95\%}$ [0.00, 0.12], $r^{JZS}_{Cauchy} = 0.71$
$\log_e(BF_{01}) = -4.58$, $\widehat{R^2}^{posterior}_{Bayesian} = 0.08$, $CI^{HDI}_{95\%}$ [0.01, 0.16], $r^{JZS}_{Cauchy} = 0.71$
$\log_e(BF_{01}) = -4.15$, $\widehat{R^2}^{posterior}_{Bayesian} = 0.07$, $CI^{HDI}_{95\%}$ [0.00, 0.14], $r^{JZS}_{Cauchy} = 0.71$

# GYKH = Grain yield, Kg/ha

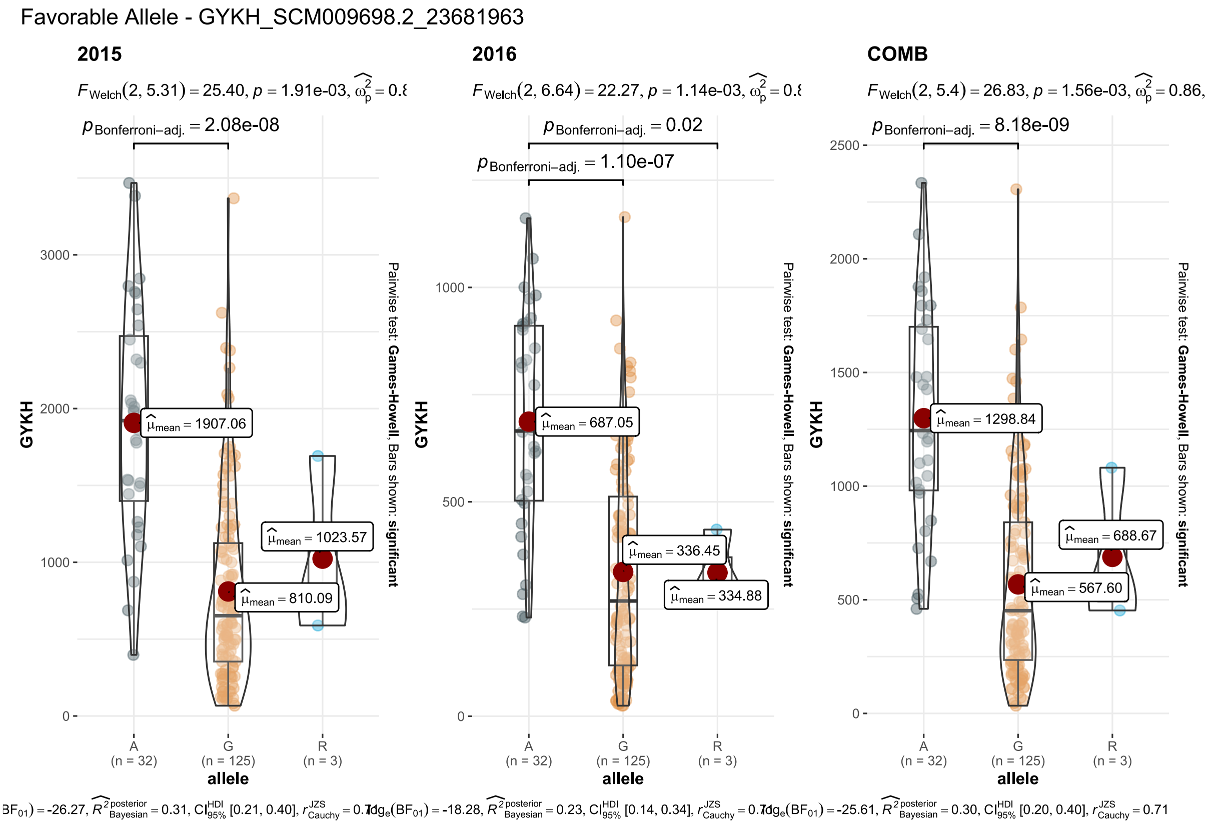

## Fe=Iron (mg/kg)

Favorable Allele - Fe_SCM009706.2_5885921

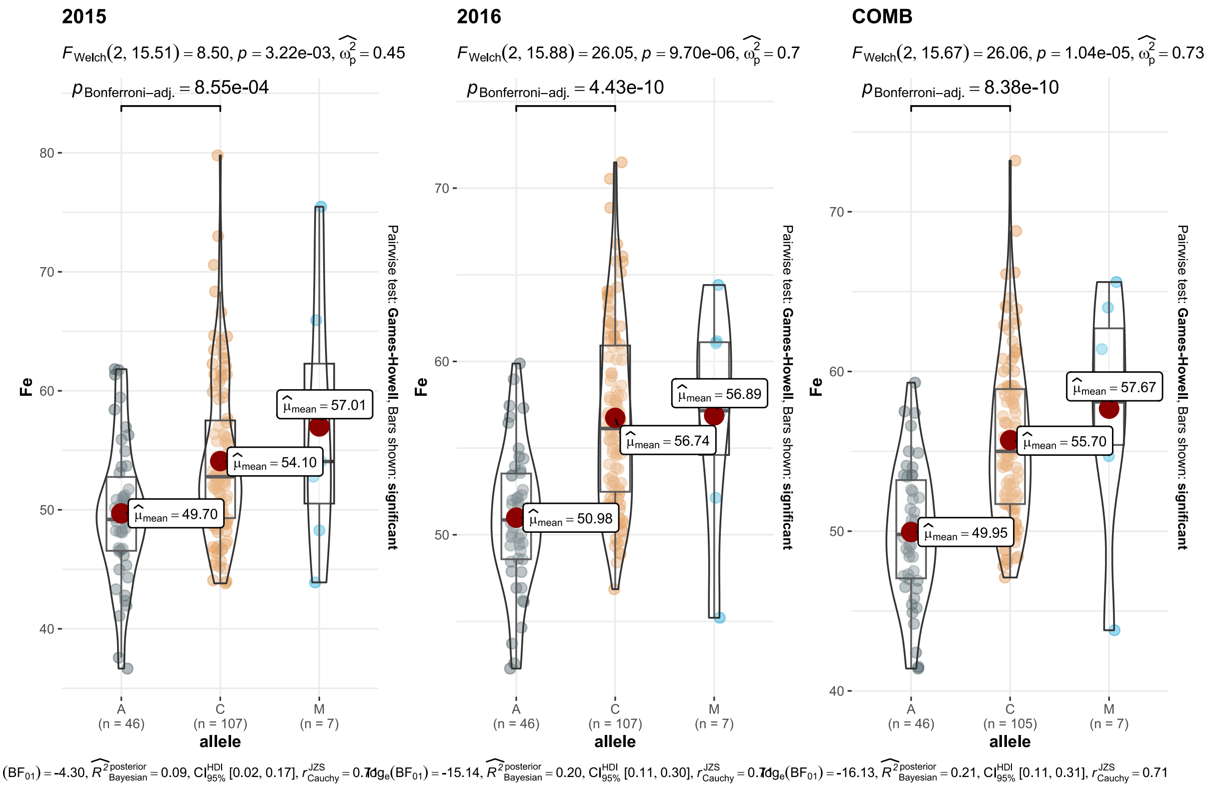


Favorable Allele - Fe_SCM009698.2_17145336

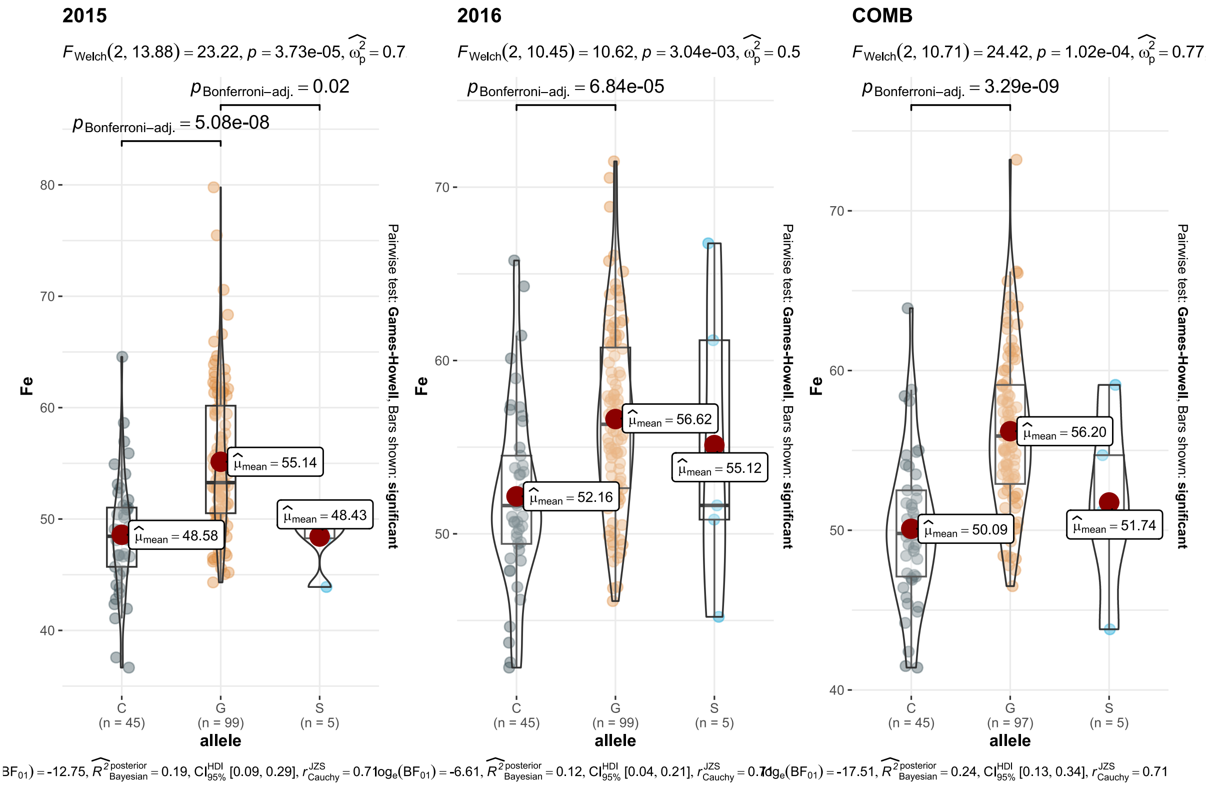

# Protein %

Favorable Allele - Protein_SCM009703.2_26492521

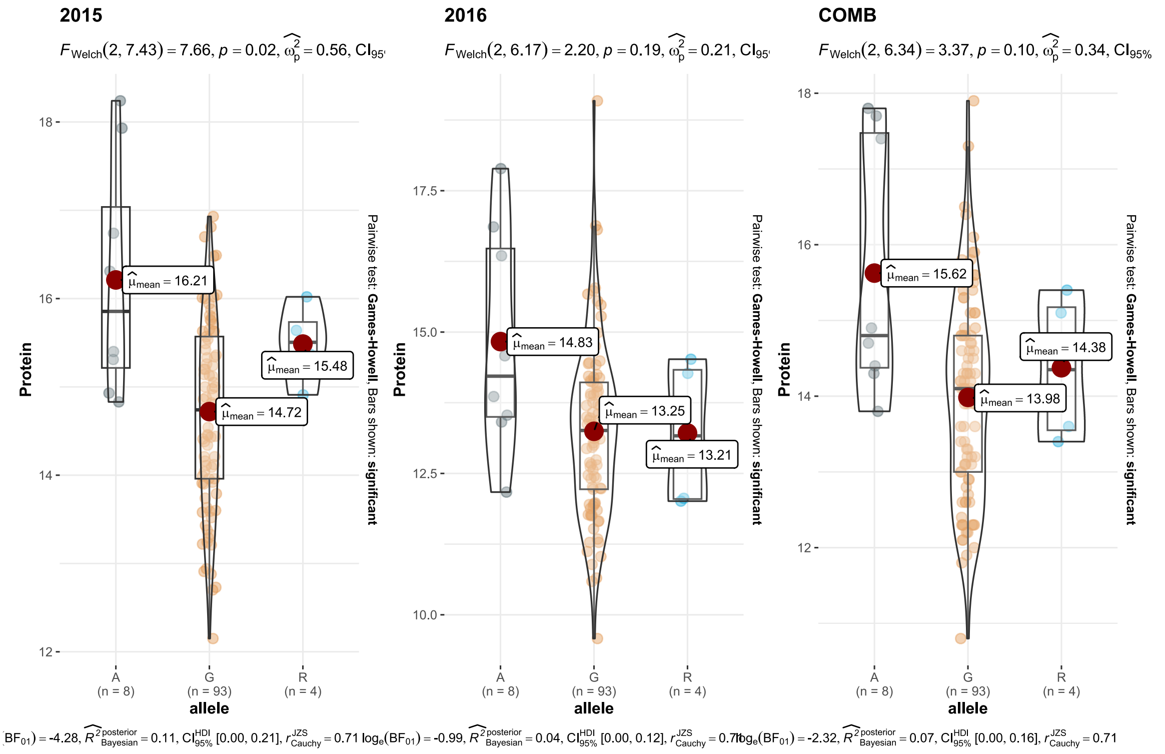


Favorable Allele - Protein_SCM009699.2_21439333

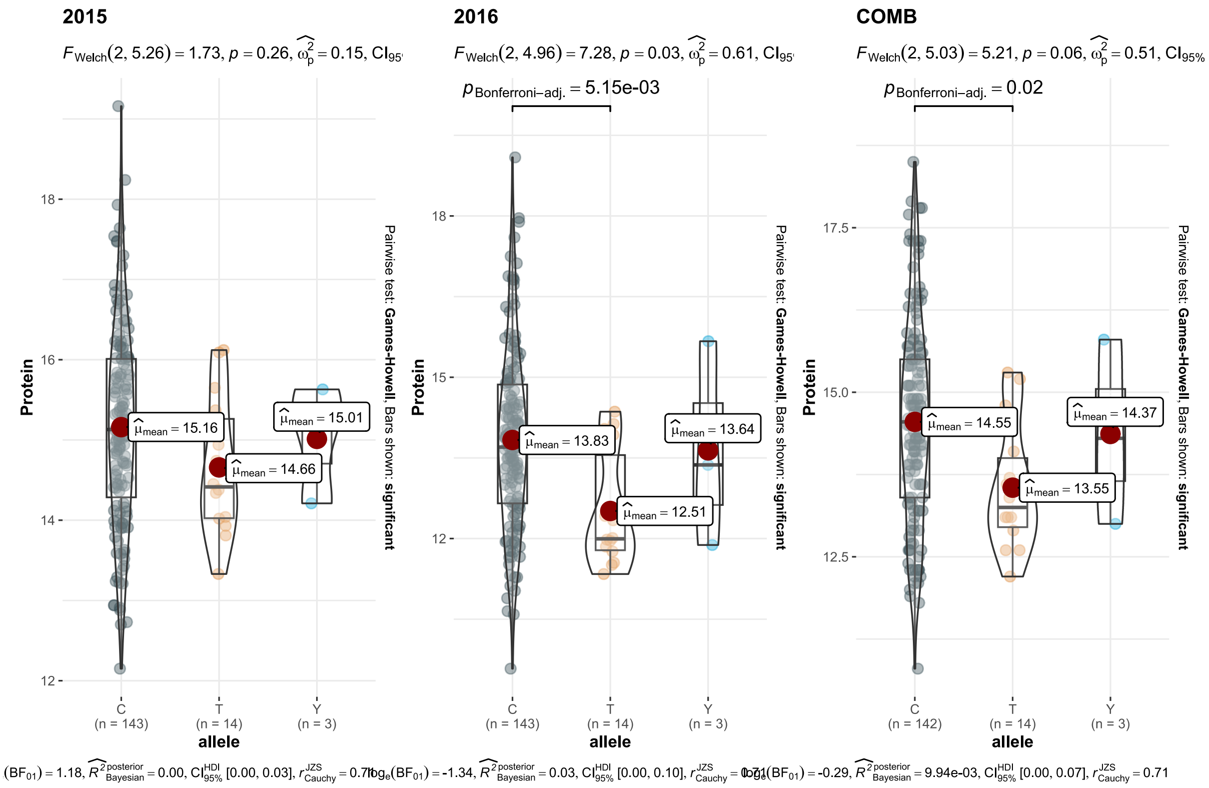

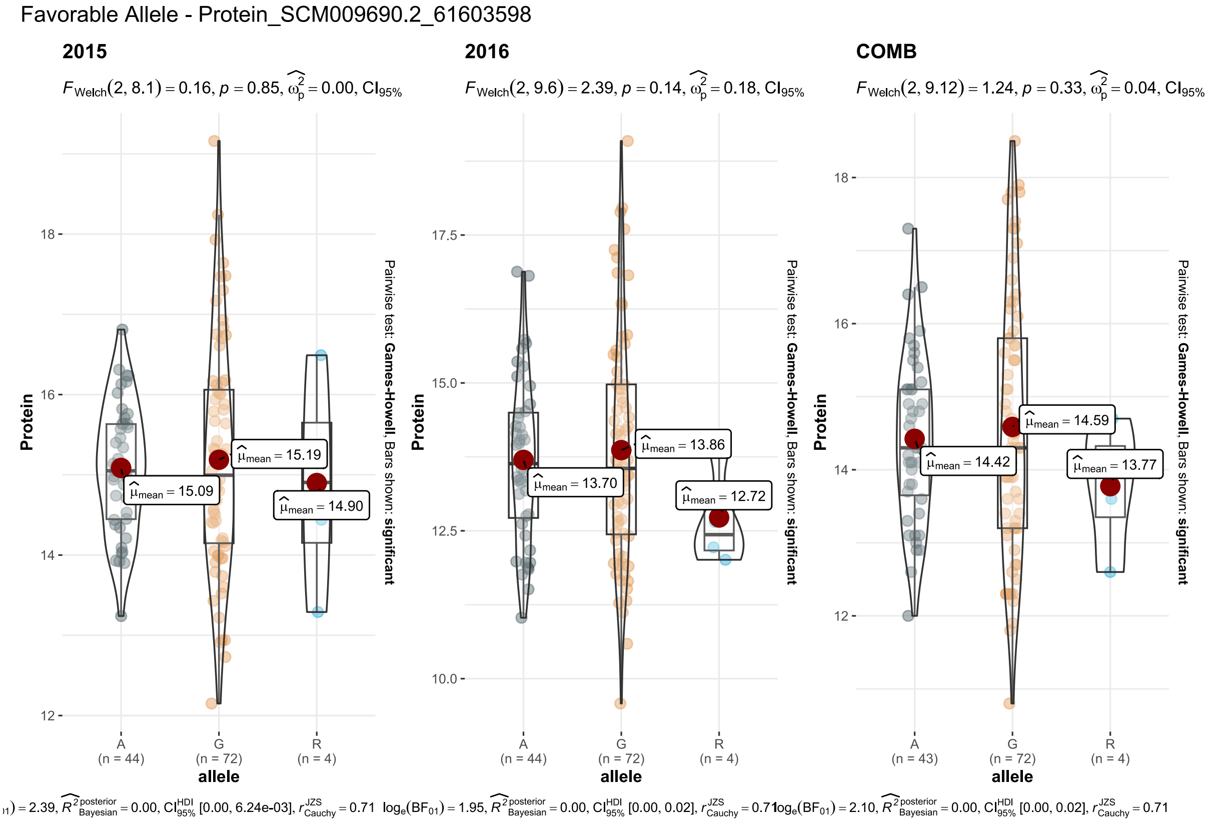
Favorable Allele - Protein_SCM009690.2_61603598
2015
2016
COMB
Protein
allele
Pairwise test: Games-Howell, Bars shown: significant
A (n = 44)
G (n = 72)
R (n = 4)
A (n = 43)
μmean = 15.09
μmean = 15.19
μmean = 14.90
μmean = 13.70
μmean = 13.86
μmean = 12.72
μmean = 14.42
μmean = 14.59
μmean = 13.77

# Zn = Zinc (mg/kg)

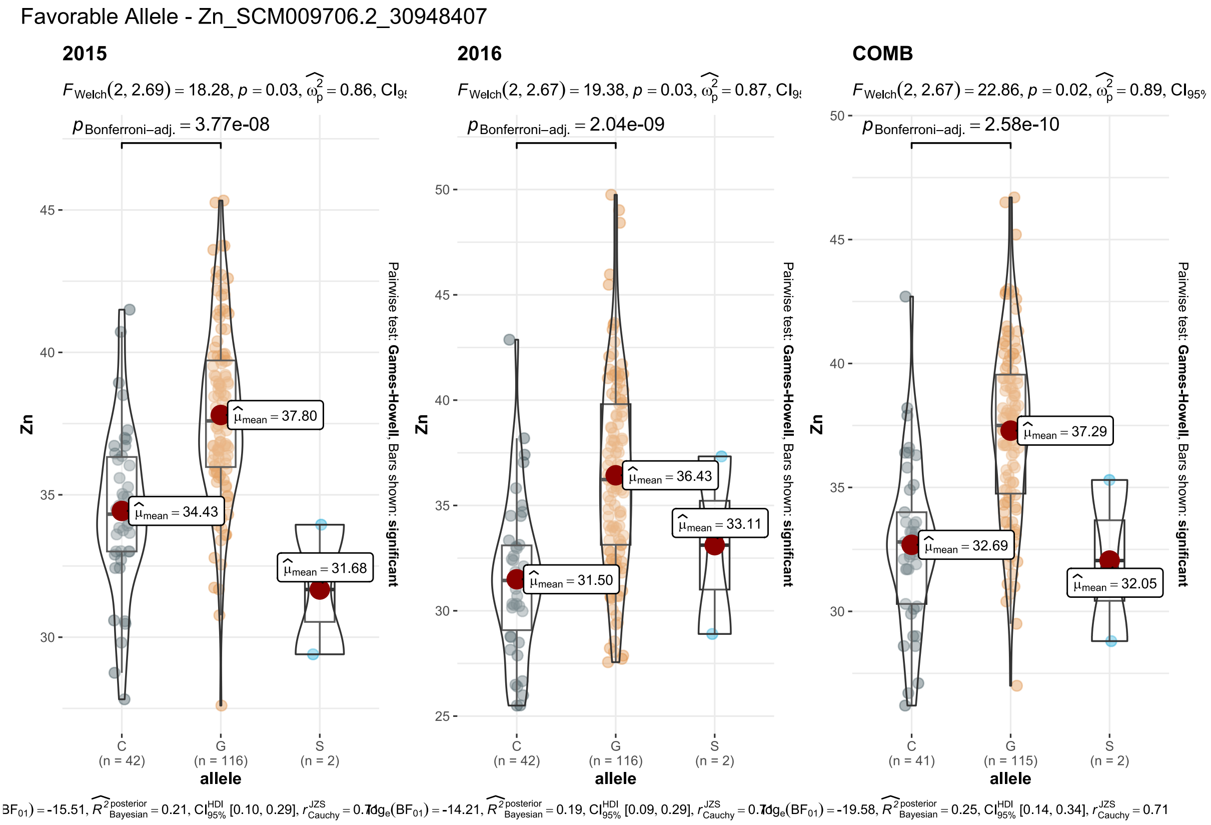

Favorable Allele - Zn_SCM009706.2_30948407
2015
2016
COMB
$F_{Welch}(2, 2.69) = 18.28$, $p = 0.03$, $\widehat{\omega_p^2} = 0.86$, CI
$F_{Welch}(2, 2.67) = 19.38$, $p = 0.03$, $\widehat{\omega_p^2} = 0.87$, CI
$F_{Welch}(2, 2.67) = 22.86$, $p = 0.02$, $\widehat{\omega_p^2} = 0.89$, $CI_{95\%}$
$p_{Bonferroni-adj.} = 3.77e-08$
$p_{Bonferroni-adj.} = 2.04e-09$
$p_{Bonferroni-adj.} = 2.58e-10$
$\hat{\mu}_{mean} = 37.80$
$\hat{\mu}_{mean} = 34.43$
$\hat{\mu}_{mean} = 31.68$
$\hat{\mu}_{mean} = 36.43$
$\hat{\mu}_{mean} = 33.11$
$\hat{\mu}_{mean} = 31.50$
$\hat{\mu}_{mean} = 37.29$
$\hat{\mu}_{mean} = 32.69$
$\hat{\mu}_{mean} = 32.05$
Zn
allele
C (n = 42)
G (n = 116)
S (n = 2)
C (n = 41)
G (n = 115)
S (n = 2)
Pairwise test: Games-Howell, Bars shown: significant
$(BF_{01}) = -15.51$, $\widehat{R^2}^{posterior}_{Bayesian} = 0.21$, $CI^{HDI}_{95\%}$ [0.10, 0.29], $r^{JZS}_{Cauchy} = 0.71$
$\log_e(BF_{01}) = -14.21$, $\widehat{R^2}^{posterior}_{Bayesian} = 0.19$, $CI^{HDI}_{95\%}$ [0.09, 0.29], $r^{JZS}_{Cauchy} = 0.71$
$\log_e(BF_{01}) = -19.58$, $\widehat{R^2}^{posterior}_{Bayesian} = 0.25$, $CI^{HDI}_{95\%}$ [0.14, 0.34], $r^{JZS}_{Cauchy} = 0.71$
